# HAMSTER: visualizing microarray experiments as a set of minimum spanning trees

**DOI:** 10.1186/1751-0473-4-8

**Published:** 2009-11-20

**Authors:** Raymond Wan, Larisa Kiseleva, Hajime Harada, Hiroshi Mamitsuka, Paul Horton

**Affiliations:** 1 Bioinformatics Center, Institute for Chemical Research, Kyoto University, Gokasho, Uji, 611-0011, Japan; 2 Computational Biology Research Center, AIST, 2-4-7 Aomi, Koto-ku, Tokyo, 135-0064, Japan

## Abstract

**Background:**

Visualization tools allow researchers to obtain a global view of the interrelationships between the probes or experiments of a gene expression (*e.g. microarray*) data set. Some existing methods include hierarchical clustering and k-means. In recent years, others have proposed applying minimum spanning trees (MST) for microarray clustering. Although MST-based clustering is formally equivalent to the dendrograms produced by hierarchical clustering under certain conditions; visually they can be quite different.

**Methods:**

HAMSTER (Helpful Abstraction using Minimum Spanning Trees for Expression Relations) is an open source system for generating a **set **of MSTs from the experiments of a microarray data set. While previous works have generated a single MST from a data set for data clustering, we recursively merge experiments and repeat this process to obtain a set of MSTs for data visualization. Depending on the parameters chosen, each tree is analogous to a snapshot of one step of the hierarchical clustering process. We scored and ranked these trees using one of three proposed schemes. HAMSTER is implemented in C++ and makes use of Graphviz for laying out each MST.

**Results:**

We report on the running time of HAMSTER and demonstrate using data sets from the NCBI Gene Expression Omnibus (GEO) that the images created by HAMSTER offer insights that differ from the dendrograms of hierarchical clustering. In addition to the C++ program which is available as open source, we also provided a web-based version (HAMSTER^+^) which allows users to apply our system through a web browser without any computer programming knowledge.

**Conclusion:**

Researchers may find it helpful to include HAMSTER in their microarray analysis workflow as it can offer insights that differ from hierarchical clustering. We believe that HAMSTER would be useful for certain types of gradient data sets (e.g time-series data) and data that indicate relationships between cells/tissues. Both the source and the web server variant of HAMSTER are available from http://hamster.cbrc.jp/.

## Background

The high dimensionality and exploratory nature of microarray data analysis has led to the application of several unsupervised data clustering techniques to aid in the visualization of gene expression data. Three popular methods are hierarchical clustering (HC) [1], k-means [2], and self-organizing maps (SOM) [3] (others have previously compared these systems [4]). Implementations of these methods can be found in TreeView [1], Cluster [5], and GENECLUSTER [3]; in more general statistical tools such as R [6]; or on-line, as part of public microarray repositories such as NCBI's Gene Expression Omnibus (GEO) [7]. Among these, the most popular is hierarchical clustering (HC), which builds a tree by recursively combining the two most similar objects.

**Figure 1:**
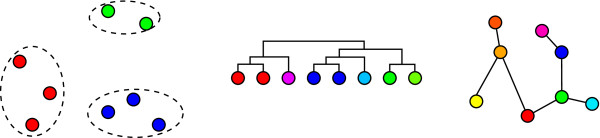
**Types of clustering and visualization methods for microarray data**. The three possible clustering and visualization techniques for microarray data (from left to right): as independent clusters, as a dendrogram, and as a minimum spanning tree (MST).

In this work, we describe a scheme primarily for visualization based on minimum spanning trees. Unlike the clustering methods described above, a minimum spanning tree (MST) is more naturally defined as a graph structure. As motivation for our work, we generalize the types of microarray clustering and visualization tools available to researchers in Figure 1. We classify the methods into three broad categories. Clustering schemes such as k-means and self-organizing maps separate the objects under consideration (i.e., experiments or probes) into independent clusters (Figure 1 (left)). An example of this would be a data set of tumor and normal tissue samples from patients. Hierarchical clustering produces a binary tree called a dendrogram, as shown in Figure 1 (center). This technique is useful if the relationship between samples resemble a hierarchical structure. The last example in the figure presents a minimum spanning tree. Unlike the dendrogram, the graph is unrooted and hierarchical relationships are not necessarily implied. While others have used MSTs for clustering (as we outline below), we believe that such a method would also be ideal for visualizing data with a gradient structure (such as time-series data). Experiments based on cell differentiation would also benefit since MSTs permit direct associations between samples from mixed levels of differentiation without forcing every sample to be at the same level (as hierarchical clustering would). This paper describes our techniques for achieving this, as well as a system called "HAMSTER" (Helpful Abstraction using Minimum Spanning Trees for Expression Relations) which embodies our ideas. Our main aim is to depict a microarray data set as a *set *of MSTs, instead of a single MST. These MSTs are individually scored and ranked to aid users. HAMSTER is released as open source under the GNU General Public License (GPL). In addition, we also provide a web server version called HAMSTER^+ ^[8] that encapsulates the local version within a set of tools for selecting parts of the data set. This web server allows users to evaluate HAMSTER without performing any local software installation. Both systems are covered in this paper, but our focus is on the local version, which we describe by first comparing and contrasting MSTs with a related technique, hierarchical clustering.

### Hierarchical Clustering (HC)

A microarray data set can be represented as a two-dimensional data table of *n *experiments and *m *probes. Even though we focus on visualizing experiments, the methods we describe are equally applicable to probes. Of course, issues such as computation time may be significantly different since the number of probes is usually many times larger than the number of available experiments.

Hierarchical clustering (HC) forms a tree called a dendrogram, in either a bottom-up (agglomerative) or top-down (divisive) fashion. In bottom-up construction, each experiment is initialized as being in its own cluster and these clusters form the leaves of the dendrogram. Recursive pairing of clusters grows the dendrogram upwards, until only a single cluster remains. Each merge step adds an internal node to the dendrogram.

**Figure 2:**
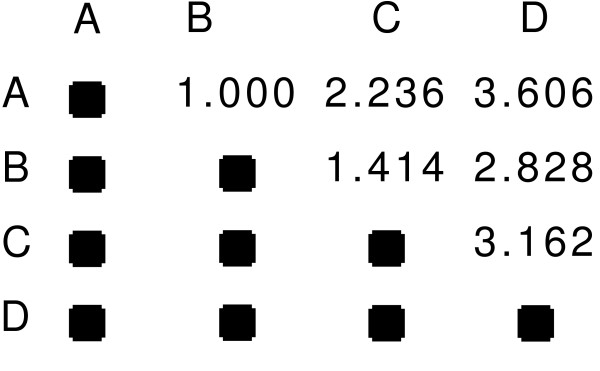
**Sample distance matrix**. The distance matrix *d *for the sample microarray of Table 1. Distances are calculated using Euclidean distance.

The calculations performed by HC requires two steps. The first is to calculate the distance matrix *d *which assigns the level of dissimilarity between every pair of experiments. Some common metrics that are used include Euclidean distance and Pearson correlation coefficient (converted to a distance by subtracting from 1). We illustrate this with a sample data set in Table 1 and the corresponding distance matrix *d *as Figure 2 using Euclidean distance. The distance matrix is calculated once and thereafter never updated. In the second step, as clusters are formed, the dissimilarity between two clusters of experiments is calculated by the linkage type chosen by the user. Three common linkages are single, average, and complete, which represent the shortest distance, average distance, and longest distance between pairs of experiments (from different clusters).

**Table 1:** Sample microarray.

	Probe 1	Probe 2
A	2	1
B	2	2
C	1	3
D	4	4

Sample microarray of four experiments (A, B, C, and D) and two probes.

**Figure 3:**
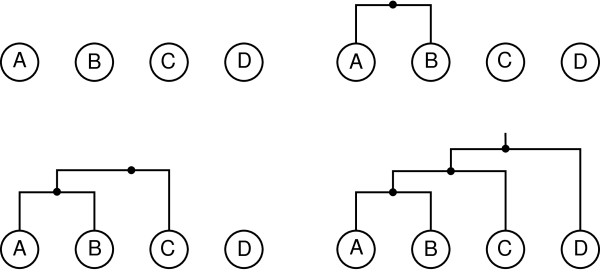
**Sample hierarchical clustering**. The hierarchical clustering process, including the final dendrogram, for the sample data set of Table 1 using Euclidean distance and single linkage.

Applying HC to the data set of Table 1 using single linkage is shown in Figure 3, including the final dendrogram (bottom-right). In every case, the four experiments are shown along the bottom and internal nodes are indicated as smaller circles. In a dendrogram, every experiment is connected to exactly one internal node.

One important property of dendrograms which we will return to later is that the two branches of each internal node can be flipped without any lost in the definition of hierarchical clustering. This gives flexibility in the order in which experiments are shown, but it also can cause problems since a dendrogram could imply an order which is not present. In our example, the positions of nodes A and B can be inverted and the dendrogram would still represent the data of Table 1.

### Minimum Spanning Trees (MSTs)

A graph is a concept in computer science which represents information as a set of nodes and edges, such that each edge indicates some relationship between its two associated nodes (for our purposes, we disallow edges which connect a node to itself). An undirected graph *G*(*V, E*) is composed of a set of vertices *V *and a set of edges *E*, with no direction on any of the edges. A *spanning tree *for *G *is a connected graph with the same vertices but only |*V*| - 1 edges and no cycles. In a *connected *graph, each node can be reached from any other node, by following a series of edges. If the edges in the original graph are weighted, a spanning tree whose edges have the minimal total weight is called a *minimum spanning tree *(MST). We denote a minimum spanning tree of *G *as *G*_*M *_(*V*, *E*_*M*_), such that *E*_*M *_⊆ *E*.

Several algorithms exist for calculating MSTs, including Prim's algorithm [9] and Kruskal's algorithm [10]. Prim's algorithm starts from an arbitrary node and extends the MST a node at a time in a greedy manner by selecting neighboring edges with the lowest weight. Instead of adding nodes, Kruskal's algorithm adds edges. It organizes the dissimilarity matrix *d *as a sorted list and adds edges, starting from the one with the lowest edge weight, if they connect two previously disconnected components. If all edge weights in *G *are unique, then the MST produced is unique, regardless of the algorithm employed. These algorithms and a description of MSTs are described in books on graph theory [11,12].

MST construction refers to the selection of nodes or edges. If this procedure is coupled with the Euclidean distance measure for determining edge weights, then some authors have referred to the MSTs as EMSTs (Euclidean Minimum Spanning Trees) [13]. We do not make such a restriction since several other dissimilarity metrics are equally important to microarray data.

In this work, we have selected Kruskal's algorithm, which we illustrate by extending our earlier example.

**Figure 4:**
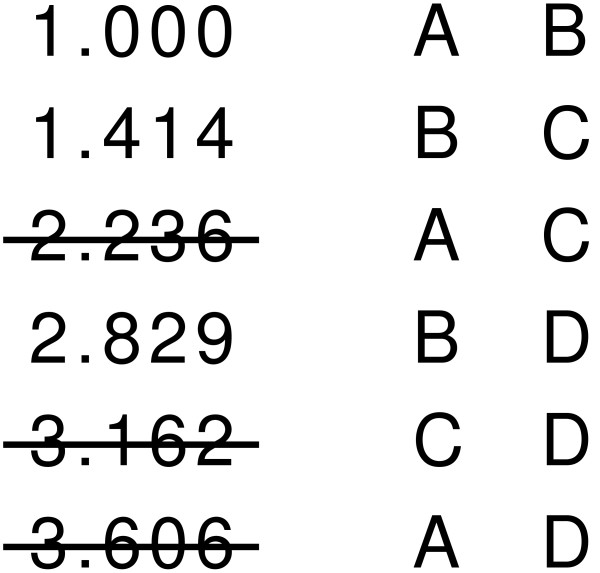
**Sample sorted list of distances for Kruskal's algorithm**. The sorted list of distances from *d *of Figure 2. Edges which are not used for MST construction are crossed out.

**Figure 5:**
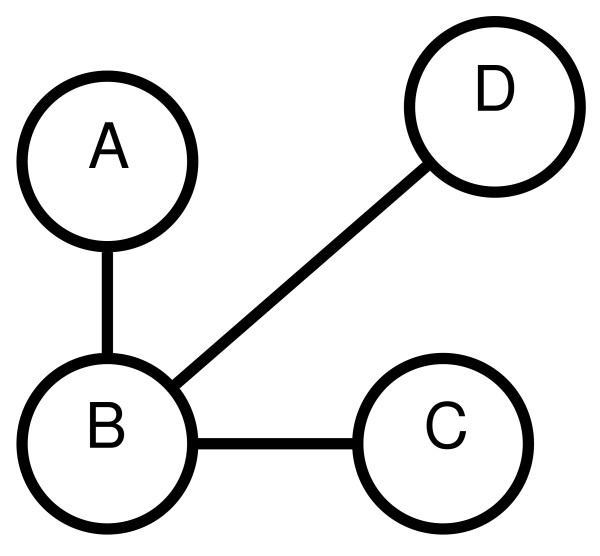
**Sample MST**. The MST corresponding to the sample data set of Table 1. Edge lengths indicate the dissimilarity between experiments.

In Figure 4, we show the distances of Figure 2 in sorted order. The resulting MST is shown in Figure 5 such that each node is an experiment and the lengths of edges indicate the level of dissimilarity between experiments.

### Dendrograms and MSTs

In our example, the dendrogram produced from HC appears similar in structure to the MST. As observed by others [14], this property can be summarized as follows:

**Property 1 ***Bottom-up hierarchical clustering using single linkage generates a dendrogram which is equivalent in structure to a minimum spanning tree generated using Kruskal's algorithm*.

The reason for Property 1 is straightforward. Hierarchical clustering using single linkage recursively chooses the smallest dissimilarity between two clusters. On the other hand, Kruskal's algorithm sorts the edge weights in decreasing order and adds edges to *G *only if they connect two separated clusters. So, if the dendrogram from hierarchical clustering is treated as a more general graph, the connections added to both graph representations are the same. If there were identical dissimilarities (edge weights), additional tie-breaking rules (based, for example, on node labels) would be required.

Basically, Property 1 implies that the same groups of nodes are connected, whether they are graph components in an MST or sub-trees in a dendrogram. Despite Property 1, dendrograms are *visually *very different from MSTs and it is this difference that HAMSTER aims to leverage.

Some of the differences between dendrograms and MSTs are as follows. A dendrogram has an orientation, so that users can examine it from the root node down to the leaves. MSTs have no obvious starting point and need to be examined as a whole. The most important difference is that dendrograms introduce internal nodes to connect clusters together while MSTs do not. In an MST, experiments are connected directly to each other, allowing users to examine the MST for hubs and neighborhoods. For example, in Figure 5, it can be seen that experiment B is a central node, in the sense that it is the nearest neighbor of A, C, and D. This point cannot be easily seen from the corresponding dendrogram.

### Previous Applications of MSTs

In bioinformatics, MSTs have been used in areas ranging from depicting the sequence repetitions in part of the *C. elegans *genome [15] to showing the shapes of disease clusters (e.g., cases of the West Nile virus) on a map [13]. In the latter case, the authors showed that the disease clusters are allowed to form arbitrary shapes, as determined by distances between objects, instead of circles as implied by Euclidean distance-based methods.

As for microarray data, our application of MSTs is most related to the work on data clustering by researchers at the Oak Ridge National Laboratory/University of Georgia. Initially, they proved that MSTs have the desirable property that all "good" clusters must consist of nodes that form a connected subgraph of the MST [16]. The sufficient condition of "good" proposed is that if a cluster of nodes from a graph *G *is split into two non-empty halves *C*_1 _and *C*_2_, the nearest neighbor of any node in *G*\*C*_1 _is in *C*_2_. Their main idea is to construct an MST from the genes in a microarray data set and then apply clustering algorithms on the MST instead of the original data. In essence, the problem of clustering the original data is converted to a simpler tree-partitioning problem. They incorporated their ideas into a system dubbed EXCAVATOR (EXpression data Clustering Analysis and VisualizATiOn Resource) [17]. Since then, they have also built CUBIC for clustering regulatory binding sites using MSTs where the vertices are *k*-mers and the dissimilarity between *k*-mers is calculated using the Hamming distance [18,19]. More recently they investigated parallelizing MST construction where the number of vertices is as high as one million [20]. Varma and Simon [2004] have used MSTs for feature (gene) selection for two-class microarray clustering [21]. In many microarray-based studies, the number of genes that are up/down-regulated are typically very small. Their aim is to use MSTs to select the genes which are most pertinent to the study. Instead of examining all combinations of genes, they only evaluated the subset of genes obtained by removing a single edge at a time from the MST. Afterwards, the experiments are clustered using hierarchical clustering and this gene subset.

Magwene et al. [2003] presented a method for identifying the time-index of biological samples by combining an MST with a data structure called a PQ-tree [22]. Assuming that changes in the transcriptome are "smooth and continuous", the samples should form a single path. Deviations from the path (branches) are checked recursively using the PQ-tree.

## Results

Previous works used MSTs for data clustering since they afford more efficient clustering. Our primary aim, however, is to use MSTs for microarray *visualization*. Rather than breaking MSTs into components to form clusters, all of our MSTs remain connected.

Based on this underlying premise, there are three main results in this paper. Instead of a single MST, we build *sets *of MSTs for a single microarray data set and show how they are similar to dendrogram *construction*. This observation is an extension to our earlier discussion where we showed how dendrograms are similar MSTs, with the internal nodes of dendrograms being the most notable difference. We then propose three schemes which score and then rank the MSTs in a set to help users select the important MSTs. Finally, we describe a publicly available system called HAMSTER that embodies these ideas and which we also demonstrate with real data. These three results are covered in the sections below.

### Set of MSTs

A set of MSTs is constructed using HAMSTER by recursively merging the most similar experiments in the original microarray data set of *n *experiments and *m *genes. To facilitate the discussion below we distinguish between the microarray and the MST views used by HAMSTER. Clusters of experiments are formed from the microarray with dissimilarities (or distances) and linkages between clusters, similar to hierarchical clustering. The abstract MST-view of these clusters has nodes and edge weights between them. This separation emphasizes that experiment merging is done on the microarray data set and one possible interpretation of the set of MSTs is that it allows users to interpret the relationships between clusters as a connected graph.

**Figure 6:**
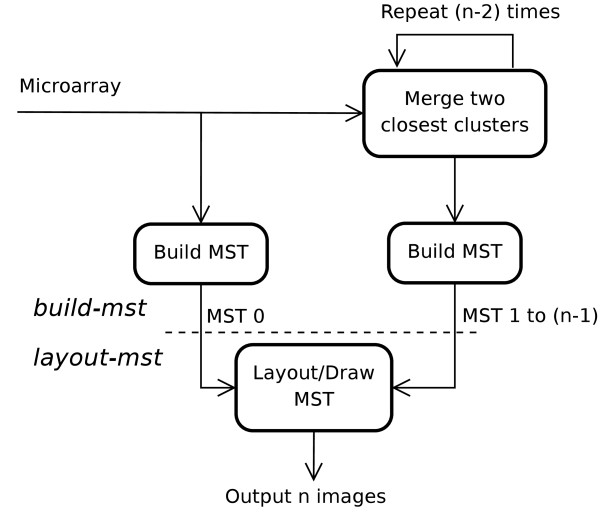
**Work flow of HAMSTER**. HAMSTER receives a microarray data set as input and, through experiment merging, produces a set of *n *images (where *n *is the number of initial experiments in the data set). There are two phases to HAMSTER, as separated by the dashed line: build-mst and layout-mst.

Figure 6 outlines the procedure used by HAMSTER. There are two phases, which we call build-mst and layout-mst.

The MSTs are constructed by build-mst as follows. Initially, each of the *n *experiments in the data set is itself a cluster. The first MST (MST 0) is obtained by directly applying Kruskal's algorithm. Then, the two most similar clusters in the microarray data set are combined and an MST of *n*-1 nodes is created from it (again, using Kruskal's algorithm) and designated as MST 1. This process continues until MST *n*-1 is formed (*n *MSTs in total), which would have a single node that encompasses all of the experiments and no edges. As with hierarchical clustering, edge weights between composite clusters are calculated through the user's selection of linkages.

The main difference between our work and what others have done previously with MSTs is the application of Kruskal's algorithm *n *times. While each merge changes the number of clusters, Kruskal's algorithm does not make use of information such as the number of experiments within each cluster.

We describe build-mst in further detail using Algorithm 1. The build-mst system constructs a priority queue of potential clusters in order to efficiently locate the next merge. When a cluster is formed, its dissimilarity with all other clusters must be calculated and inserted into the queue. The priority queue of dissimilarities is implemented as a heap and Kruskal's algorithm sorts these dissimilarities to build the MSTs [11].

**Algorithm 1**: Pseudocode depicting the merging scheme of build-mst. Translating the description of the *n *MSTs into images is performed by layout-mst (not shown here).

**Data**: Microarray data set X (*n *experiments × *m *probes)

**Result**: *n *MSTs ()

**1 **C ← initializeClusters(X)

**2 **D ← calculateDistances(X)

**3 **PQ ← buildPQueue(D)

**4 ** ← buildMST(C, PQ)

**5 for ***i *← 1 **to ***n*-1 **do**

**6 **   C ← mergeClusters(C)

**7 **   PQ ← updatePQueue(PQ)

**8 **    ← buildMST(C, PQ)

9 end

The output of build-mst is a set of MSTs such that each edge in the MST also has an edge weight and every node has an attribute (color and shape). Edge weights are normalized out of 1.0 for each MST and are used to indicate the relative distance between nodes when the MST is drawn. As for attributes, a user has the option of assigning them to the experiments of the data set, which are then passed along as merging progresses. If two clusters with the same attributes are merged, then the associated MST node obtains the same attributes; otherwise, it is assigned default attributes. Creating a graphical MST from this information is done by the layout-mst phase, which lays out the graph by compromising between the distances between nodes (the edge weights) and minimizing the number of node overlaps.

**Figure 7:**
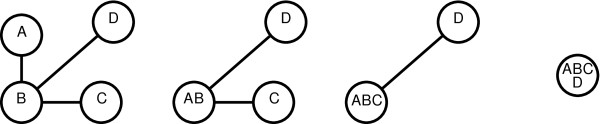
**The MSTs created though experiment merging**. Initially, the number of clusters is equal to the number of experiments. Successive merging of the *n *experiments produces *n *MSTs with the last one having a single node and no edges. MSTs are numbered from 0 by HAMSTER.

We illustrate this process by continuing our earlier example. The four MSTs produced from the microarray data set of Table 1 using Euclidean distance and single linkage are shown in Figure 7.

By comparing Figure 3 with Figure 7, we can see that the set of MSTs is analogous to taking snapshots at each iteration of the hierarchical clustering process. At each step, the corresponding images show the relationship between the clusters.

### Scoring and Ranking MSTs

While some users may be satisfied with the set of images produced by our method, others may require further guidance by having each image scored individually. This is analogous to users having to decide where to cut a dendrogram to give meaningful sub-trees. We investigated several scoring schemes for the purpose of MST ranking and implemented three: gap-based, ANOVA, and normalized association.

The gap-based and ANOVA schemes are based on the idea that the dissimilarities in the distance matrix can be separated into *two *disjoint groups: those that are within a cluster (intra-cluster) and those that are between clusters (inter-cluster). Starting from all of the distances in the inter-cluster group, the merging process used by HAMSTER moves distances from this group to the intra-cluster group until the former is empty and only a single cluster remains. Since distances are chosen in increasing order, these schemes determine the point at which many small distances are in the intra-cluster group and many large distances are in the inter-cluster group.

The gap-based scheme reports on the difference in distance between the largest intra-cluster distance and the smallest inter-cluster distance. The score is given a value of 0 when either groups are empty. In contrast, the ANOVA scheme calculates the analysis of variance between the means of the two groups. The score is the F-statistic, which is the variation between the two groups divided by the variation within the groups. In both cases, a large score indicates an MST whose two groups of distances are well-separated. The third scoring scheme is the normalized association (), which makes direct use of the clusters created through merging and has proven itself in problems related to image segmentation [23]. In a weighted, undirected graph, the association *A *between two clusters *X *and *Y *is the sum of the dissimilarities (or linkages) between two clusters:(1)

The sum of all dissimilarities within a cluster *X *is denoted as *A*(*X, X*) and the sum of all dissimilarities which have at least one experiment in *X *is *A*(*X, Z*), where *Z *is the set of all clusters. From this definition, we can see that *A*(*X, X*) ≤ *A*(*X, Z*). The normalized association () for an MST is the sum of the proportions of dissimilarities across all clusters:(2)

The normalized association measures how well the experiments within a cluster are associated with each other, relative to other experiments. In the case of HAMSTER, because every experiment has a dissimilarity to every other cluster, the largest normalized association is the trivial case of one cluster containing all experiments. Furthermore, the normalized association monotonically increases with each iteration. To correct this, we multiply Equation (2) by the number of clusters (|*Z*|). This increases the score if there are more clusters.

These scoring schemes are used to evaluate MSTs but are actually calculated by examining the effect merging has on the underlying distance matrix. The scoring schemes are all normalized so that they are percentages of the highest score. As a starting point for users, we suggest the gap-based scheme as it is the most familiar to people who employ hierarchical clustering. As suggested earlier, in a dendrogram like Figure 3, clusters are formed by cutting it horizontally to form many trees. Examining the gaps is analogous to assessing the point where such a cut should be made.

### Implementation

We describe our implementation of HAMSTER and, in less detail, its web server variant called HAMSTER^+^. The HAMSTER system and access to HAMSTER^+ ^are both available at http://hamster.cbrc.jp/.

HAMSTER is open source and distributed under the GNU General Public License (version 3 or later). The two parts of the HAMSTER system represent two separate executables (build-mst and layout-mst). The source code is written in C++ and documented in-line such that Doxygen [24] could be used to produce documentation for users who would like to extend HAMSTER's features (See additional files 1 and 2: build-refman.pdf and layout-refman.pdf, respectively.). The software was successfully compiled using Autotools and v4.3.2 of the g++ compiler running under Linux.

HAMSTER makes use of other external software or libraries which must be downloaded and installed separately, as shown in Table 2. The Boost library for C++ is required and must be both installed and compiled according to the instructions in the accompanying README file. If Graphviz [25] is absent, then only Graphviz source files are produced. The Open MPI library is also optional but, if properly installed, can be used to distribute the work of layout-mst across multiple CPUs.

**Table 2:** Additional software and libraries used by HAMSTER.

Software	Minimum Version	Purpose	System	Required?
Boost library [32]	1.39.0	Used extensively	Both	Yes
Graphviz (neato) [25]	2.20.2	MST layout and generation	layout-mst	No
Open MPI [26]	1.2.7	Parallel processing of MST layout	layout-mst	No

The software and libraries that are used by HAMSTER, as well as their version and purpose. Only the first is required to compile the software; the others are optional. Graphviz is required to generate images while Open MPI improves the execution time of layout-mst by distributing work across processors.

The features available from HAMSTER are described next, with a summary in Table 3. Options to both executables are provided on the command-line and in a configuration file called either build-mst.cfg or layout-mst.cfg. Command-line options take priority over the configuration file. To obtain a list of available options, use the option --help.

**Table 3:** Summary of the features of HAMSTER.

Program	Feature	Options
build-mst	Dissimilarities	Euclidean, Manhattan, Pearson correlation, and Spearman correlation
build-mst	Linkages	Single, Average, Complete, and Centroid
build-mst	Centroid linkage types	Euclidean, Manhattan, Pearson correlation, and Spearman correlation
layout-mst	Colors and shapes	Same as Graphviz
layout-mst	File formats	PNG, SVG, and Postscript; additional formats supported by Graphviz available by modifying the source code

The features currently offered by HAMSTER, with the relevant system indicated in the first column.

#### Running build-mst

The filename of the input microarray data file is required without any option flags. The format of the microarray data for HAMSTER is a tab-separated file with row and column labels included. All other values in the data file must be either floating point values or the string NULL to indicate a missing expression level. While our description of HAMSTER focuses on the experiments, in practice, the system can also be applied to the probes as well by transposing the data file.

An optional tab-separated file can be provided with the --attr option which describes each experiment's attributes. Every experiment and every node in each MST has two attributes: a shape and a color. The set of acceptable shapes and colors are defined by Graphviz with a few options listed in the README file. If a cluster consists of a mix of attributes, then the MST node which represents it obtains the default attribute of a "gray ellipse".

Four dissimilarity measures and four types of linkages are provided by build-mst, as summarized in Table 3. The four dissimilarity metrics are: Euclidean distance, Manhattan distance, Pearson correlation coefficient, and Spearman rank correlation coefficient. The latter of these two are converted to dissimilarity measures by subtracting from 1. The four available linkages are: single, average, complete, and centroid. The last linkage calculates the centroids of the two clusters and then the dissimilarity between them. The type of dissimilarity between centroids is typically the Euclidean distance, but the user can specify others using --centroid. Additional distance and linkage measures can be added by modifying vect_dist.cpp and cluster_link.cpp, respectively.

The software archive includes a sample configuration file, the sample data set of Table 1 as sample.data, and its corresponding attribute file called sample.attr. A sample application of build-mst would be:

build-mst sample.data --attr sample.attr

A summary of the merging process (summary.txt) is produced which shows information about each merge step.

#### Running layout-mst

After build-mst has calculated the MSTs, the layout-mst system generates the images. The only required argument is the summary created by build-mst. All other files are assumed to be in the same directory. Additional options are available which can be used to change the size or resolution of the images. The following command would use layout-mst with the sample data and default options:

layout-mst summary.txt

The layout of the images is performed externally by Graphviz. The result from this example is a series of images similar to the MSTs of Figure 7. Actual images may differ since the exact placement of nodes is determined by Graphviz, which is executed independently for each MST. An option (--fixedpos) is available which lays out each MST using the previous MST's node positions as starting points in order to minimize the visual differences between them.

Since these images are generated independently, layout-mst is also able to make use of MPI (Message Passing Interface) to distribute the workload to multiple CPUs. Details on how to do this is shown in the README file. The system has been tested with Open MPI v1.2.7 [26], but other libraries that follow the MPI standard should work.

Either --fixedpos or MPI can be used, but not both. This is because enabling MPI distributes the workload across multiple processors, while fixing the positions of nodes requires layout-mst to process the MSTs in sequential order.

A final time-savings measure that can be used with MPI is the --percent option which indicates the percentage of images to generate, starting with the ones corresponding to the MSTs with the highest scores.

#### Web Server - HAMSTER^+^

HAMSTER^+ ^adds a wrapper around the local version of HAMSTER and is also available from http://hamster.cbrc.jp/. Further details about HAMSTER^+^are provided in an on-line tutorial. Its main features are:

• No user login or local software installation is required.

• Support of microarray data from NCBI's Gene Expression Omnibus (GEO) in their Simple Omnibus Format in Text (SOFT) [7]. GEO data sets (GDS) can be referred to by their unique accession number and downloaded from NCBI's ftp server.

• The web interface allows experiments to be selected from the microarray data set.

• 559 experiments have been manually classified into 87 categories for the purpose of assigning initial attributes to them.

• Seven GEO platforms that encompass 19.3% of the 275,665 experiments in GEO (as of January 6, 2009) have been mapped to Gene Ontology categories [27]. This allows the probes of the microarray data to be selected based on gene functions.

• The MST images and their respective Graphviz sources can be browsed and downloaded.

• Each data set to be processed is assigned a unique URL that users can send to collaborators or use later for viewing. We recommend that users concerned about privacy to use the local version instead.

### Testing

We report on the expected running time of HAMSTER on various GEO data sets and then examine the output from both hierarchical clustering and HAMSTER for three of these data sets. For the first data set we consider all aspects of HAMSTER, including its similarity (and dissimilarity) with hierarchical clustering and the results from MST scoring. As for the remaining two data sets, we direct our attention to data sets which involve a gradient of sample categories which we believe make them suitable for visualization using MSTs. Since hierarchical clustering is not part of HAMSTER, we have chosen to use the agnes function of the cluster library for R to create the dendrograms [6,28]. We employ varies types of linkages in our examples to illustrate the possibilities with HAMSTER.

#### Execution time of HAMSTER

Table 4 presents the results from applying HAMSTER to six data sets from GEO. The table shows the dimensions of the data set in the second and third columns. In these experiments, we use the entire data set, irrespective of any additional information. For example, GDS596 contains 158 experiments of two sets of replicates. Thus, it would be more appropriate to apply the system to only 79 of the experiments. Nevertheless, the purpose of these results is to demonstrate the execution time of HAMSTER. Two of the data sets (GDS1962 and GDS2771) are currently the two largest data sets (in terms of number of experiments) in GEO. As the table shows, typical data sets are usually much smaller.

**Table 4:** Dimensions of GEO test data and running time and memory usage of build-mst.

	Data set size		Execution of build-mst	
		
Data set	Experiments	Probes	Elapsed time (s)	Memory usage (MB)
GDS596	158	22283	23.28	201.391
GDS1962	180	54681	66.65	383.980
GDS2765	13	45101	1.03	28.305
GDS2771	192	22283	33.03	276.352
GDS3069	12	22283	0.52	25.000
GDS3216	12	22810	0.56	25.758

The dimensions of the GEO test data are expressed as the number of experiments and the number of probes. The execution time of build-mst is reported in seconds, averaged across 5 trials, for a 2.4 GHz Intel Core 2 Quad CPU (Q6600) with 8 GB RAM. The maximum memory usage was not more than 400 MB for the largest data set.

All of our experiments were run on an otherwise idle 2.4 GHz Intel Core 2 Quad CPU (Q6600) with 8 GB RAM. Running times are reported as seconds and averaged across 5 trials.

The running time and memory usage of build-mst are shown as the last two columns of Table 4. The longest time is associated with GDS1962, which takes just over 1 minute. As expected, the running time is more dependent on the number of experiments than the number of probes, as shown by comparing the results of GDS596 with GDS2765. As for memory, the data set that gave the peak memory usage was GDS1962 at almost 400 MB.

**Figure 8:**
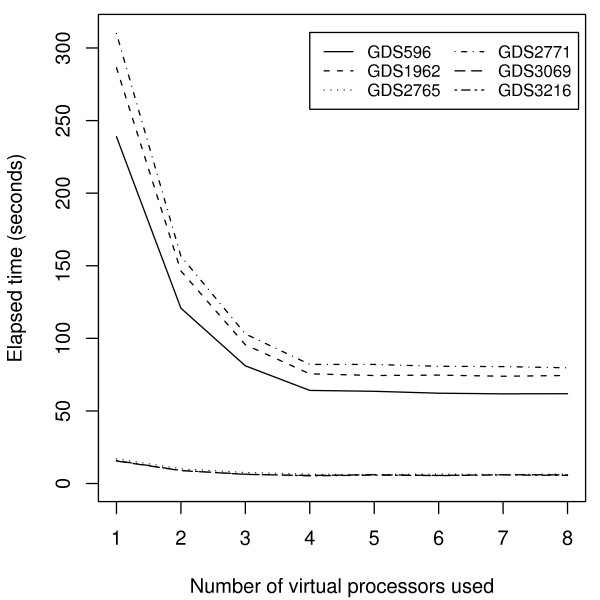
**Running time of **layout-mst** as a function of the number of processors**. The running time for six different data sets from NCBI GEO are shown after averaging across 5 trials. The system used is a 2.4 GHz Intel Core 2 Quad CPU (Q6600) with 8 GB RAM and the number of virtual processors range from 1 to 8. The files generated were Portable Network Graphics files (PNG) at a resolution of 300 dots per inch (DPI). (Higher resolutions require more time.)

In these experiments, we did not consider the running time of HC since it would not be comparable to the time required by HAMSTER. HC produces a single dendrogram while HAMSTER creates *n *distinct MSTs. As for the layout-mst system, the number of samples equals the number of images that need to be generated. We execute layout-mst using MPI while varying the number of virtual processors from 1 to 8. These results are shown in Figure 8. The maximum running time is just over 5 minutes, which decreases as the number of virtual processors increase. This decrease levels off after 4 virtual processors since this is also the number of cores present in our system. In other results (not shown), disabling MPI gave performance close to the results for 1 processor. Thus, the overhead of using MPI in our implementation appears to be negligible.

#### GDS2765: Effect of Creatine on Mice

As an example, we apply both HAMSTER and hierarchical clustering to the data set GDS2765, where researchers investigated the effect creatine has on the expression level of brain tissue in mice [29]. There are 13 samples in total and only two classes: untreated/control (7 samples) and creatine-treated (6 samples) mice. In this example, we have chosen Euclidean distance and single linkage for both so that hierarchical clustering is directly comparable to the corresponding MSTs. We should emphasize that in both cases, the methods are not provided with information about the experiment type (untreated or treated), except in the sense that we chose the color associated with each experiment based on its type (untreated in red; treated in blue).

**Figure 9:**
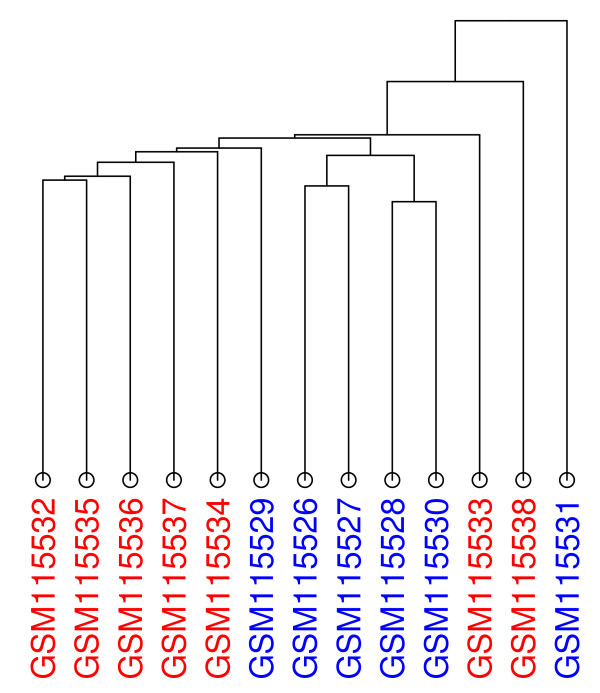
**Dendrogram for GDS2765**. The dendrogram was constructed using Euclidean distance and single linkage. The control samples are in red; the treated ones are in blue.

**Figure 10:**
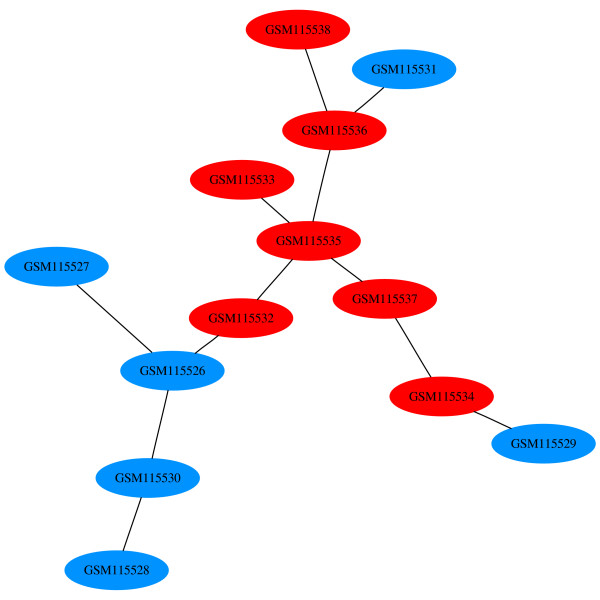
**MST 0 for GDS2765**. The first MST generated using HAMSTER using Euclidean distance and single linkage. The controls and treated samples are colored red and blue, respectively, similar to Figure 9. Its normalized association score is 0.

In Figure 9, the dendrogram for GDS2765 shows at least two groups - the five controls on the far left, followed by five treated samples. Four of the treated samples appear as one such group in the center. As with the example dendrogram of Figure 3, there is no significance in the fact that these four samples are in the center, since they can be swapped with the sub-tree at the far left and the dendrogram is still valid. Turning our attention to the MSTs, MST 0 (Figure 10) shows that the 7 control samples (in red) are similar to each other and group together in the center. This is in contrast to the 6 treated samples (in blue) which are spread across three different areas. The four treated samples on the left correspond to the aforementioned group of experiments at the center of the dendrogram. There are some differences between MST 0 and the dendrogram, though. In the center of MST 0, GSM115535 is similar to four other experiments, as indicating by the four edges emanating from it. If we compare this to the dendrogram (the second from the left sample), we note that this is not as evident. This is because an MST can have a node connected to any number of other nodes. In a dendrogram, though, the structure is restricted to recursive, pair-wise relationships.

**Figure 11:**
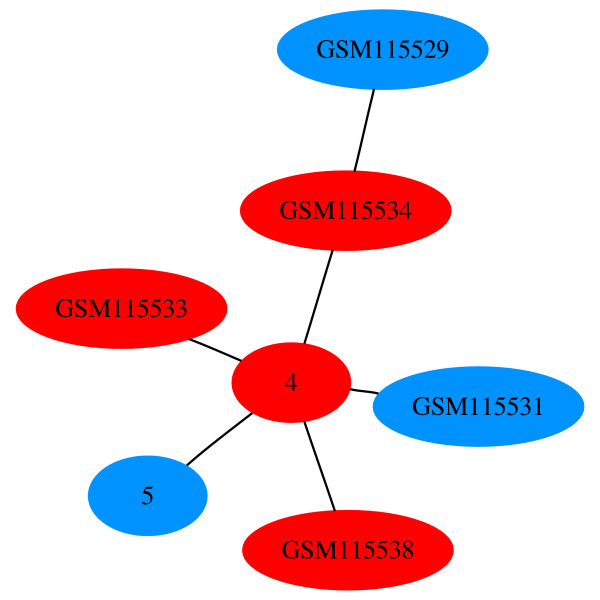
**MST 6 for GDS2765**. The node colors correspond to those of Figure 10. So far, control and treated samples have not yet mixed within any node. The normalized association score is 97.78.

**Figure 12:**
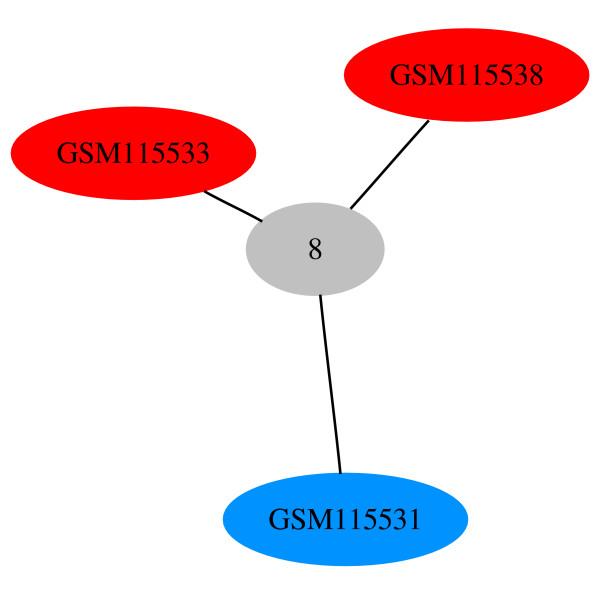
**MST 9 for GDS2765**. The colors of the nodes correspond to those of Figure 10. The node with the default attributes (gray ellipse) contains experiments with different attributes. The normalized association score is 100.

As we advance to MST 6 (Figure 11), we note that four of the treated samples have merged to form node 5 and four of the control samples have merged to form node 4. Merged nodes in HAMSTER are represented by successive integer numbers, starting from 0. MST 6 has a high normalized association score of 97.78. The highest scoring MST is MST 9 (Figure 12). In this figure, only three experiments remain by themselves and all other experiments have merged into node 8. The node has the default color and shape attributes since it has a mix of both attribute types.

**Figure 13:**
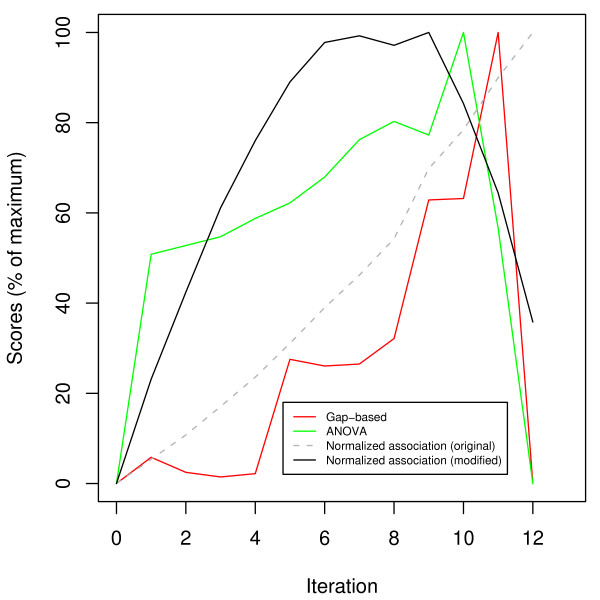
**Comparison of scoring schemes for GDS2765**. A comparison of the scoring schemes available to HAMSTER corresponding to the MSTs of Figures 10, 11, and 12. All schemes represent scores as a percentage of the maximum to facilitate easy comparisons. The iteration of HAMSTER is shown on the horizontal axis. As a comparison, the dashed line indicates the result from applying the original definition of normalized association [23] prior to the multiplication by the number of clusters.

We conclude this example with a graph comparing the various scoring schemes in Figure 13. We show all three scoring schemes as well as the original normalized association which does not multiply the score by the number of clusters. As reported earlier, the original normalized association grows monotonically, while the modified one now peaks in the middle. The remaining two scoring schemes (Gaps and ANOVA) grow steadily and peak after the half-way point.

The different peaks in this graph show that there are multiple ways in evaluating how well the experiments of a data set cluster, regardless of the clustering method. We suggest that users try the scheme which is most suitable for their needs, based on the definitions given earlier.

#### GDS3069: Various brain tumors

Our next sample data set is GDS3069 which was used to analyze 12 primary brain tumors based on their histological diagnoses [30]. Unlike the previous data set of two distinct categories, this one has a gradient of 5 categories with overlaps between them. Also, the number of samples per category varies greatly - for example one category has only one sample. The categories and the coloring that we have chosen are: high grade glioblastoma (red), high grade gliosarcoma (yellow), high grade glioblastoma/gliosarcoma (violet), low grade oligodendroglioma (green), and low grade anaplastic mixed glioma (blue). Euclidean distance and average linkage have been selected for this analysis.

**Figure 14:**
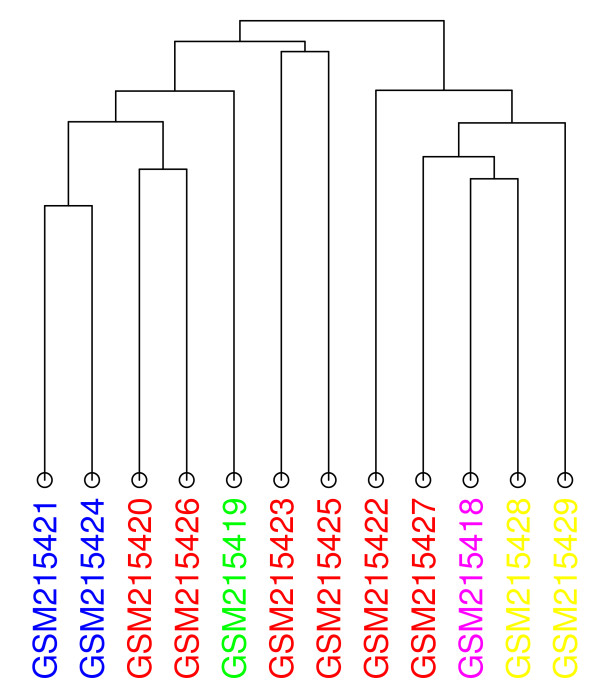
**Dendrogram for GDS3069**. This data set reports on the expression levels of 12 primary brain tumors, divided into 5 categories. The colors assigned to them are: high grade glioblastoma (red), high grade gliosarcoma (yellow), high grade glioblastoma/gliosarcoma (violet), low grade oligodendroglioma (green), and low grade anaplastic mixed glioma (blue). Euclidean distance with average linkage has been selected.

In the dendrogram of Figure 14, the four consecutive high grade glioblastoma samples (red) appear to be similar. However, upon closer inspection of the tree, we notice that the first two samples (GSM215423 and GSM215425) are grouped together in the same sub-tree, while the third (GSM215422) is combined with the four samples to its right at an earlier step.

**Figure 15:**
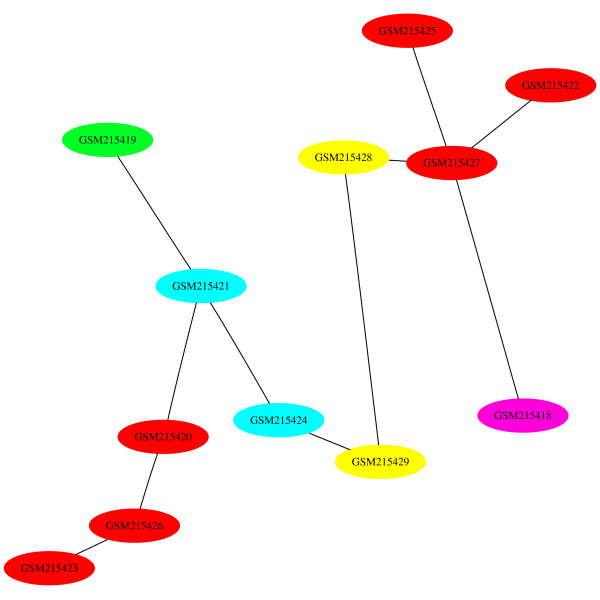
**MST 0 for GDS3069**. The nodes of the first MST have been colored in the same way as Figure 14. Euclidean distance with average linkage has been selected.

In the first MST (Figure 15), the high grade glioblastoma samples (red) are separated into two groups. However, the edges between the nodes within these two groups are relatively short, an indication that the samples are similar to each other in terms of expression level. Longer edges indicate a high level of dissimilarity, and for this data set, occurs between the two high grade gliosarcoma (yellow) samples. This example demonstrates the use of MSTs for data sets where the sample categories can be placed on a scale.

#### GDS3216: Whole seedling roots response to salinity stress: time-course

**Figure 16:**
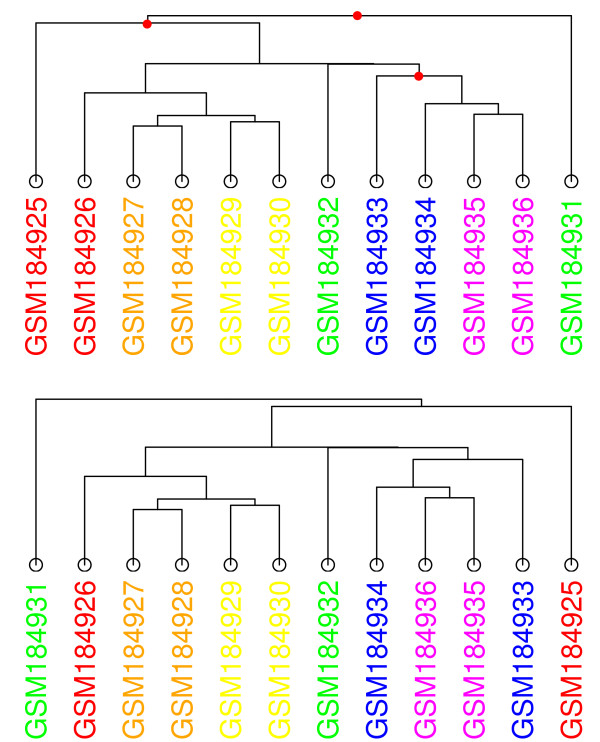
**Dendrogram for GDS3216**. The study associated with this data set measured the response *A. thaliana *seedling roots have to salinity in a time-course experiment. The nodes are colored according to the colors of the spectrum: 0 hours/control (red), 0.5 hours (orange), 1 hour (yellow), 4 hours (green), 16 hours (blue), and 32 hours (violet). Manhattan distance with average linkage has been chosen. The top dendrogram is the default one produced by agnes. By swapping the branches at the internal nodes indicated in red in this dendrogram, the one below is produced.

**Figure 17:**
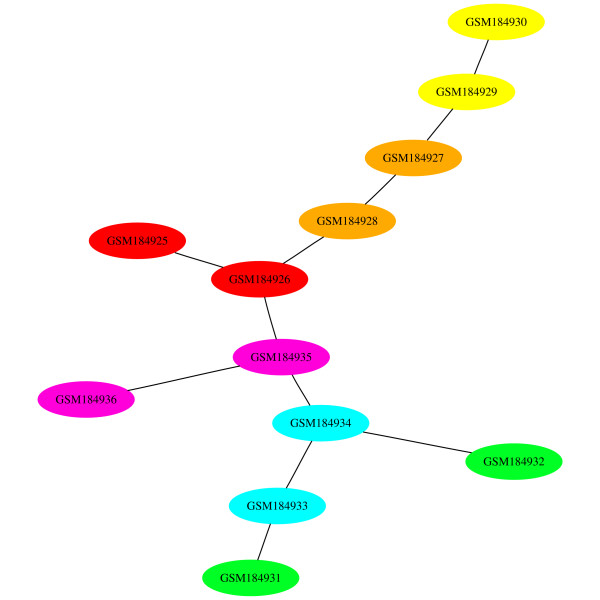
**MST 0 for GDS3216**. The nodes of the first MST have been colored in the same way as Figure 16. Manhattan distance with average linkage has been selected.

The presence of a scale is more obvious in our final example since this data set is associated with a time-course experiment (GDS3216). The experiment examines the effect salinity has on *A. thaliana *seedling roots [31]. Six time points are available with two replicates each: 0 hours (control), 0.5 hours, 1 hour, 4 hours, 16 hours, and 32 hours. We chose Manhattan distance and average linkage this time. The samples are colored according to the colors of the spectrum. In other words, these 6 time points are colored red, orange, yellow, green, blue, and violet for the control to the 32 hours time points, respectively. At first glance, the dendrogram and MST 0 appear similar (top of Figure 16 and Figure 17). In both cases, we would expect replicates to be adjacent to each other. Then, the experiments would be ordered based on time. For an MST, others have shown that a chain of experiments would be formed, ordered by the time-index [22].

The images show that the replicates are generally adjacent to each other in both the dendrogram at the top and MST 0. It seems that the dendrogram gives a better view of the time-series data set than the MST. The colors indicate that the experiments in the dendrogram are ordered by time-index, with the exception of GSM184931 at the far right. In the MST, we have a gradient split into two parts. The first six experiments appear at the top and the last six at the bottom, with GSM184932 (green) lying away from this latter group. Rather than connected end-to-end, a red experiment is connected to a violet one.

However, as suggested earlier, there are many possible permutations to a dendrogram that would be valid. In the dendrogram of the top of Figure 16, we can swap the branches at the three internal nodes indicated in red to form the dendrogram below it. These swaps have moved GSM184931 (green) to the far left and GSM184925 (red) to the far right. GSM184933 (blue) has also shifted to the right side of its sub-tree. While three pairs of replicates remain together (yellow, orange, and violet) since they are part of their own sub-tree, these swaps have shown that a different dendrogram could easily have been generated. It appears that the agnes function for R uses the order of the experiments in the original data sets to determine the final dendrogram leaf order. The reason why the top dendrogram is produced is that the experiments appear in time-index order in the original GEO data file. This would be a useful feature if the natural ordering of the data is both known a priori and is valid for visualization.

**Figure 18:**
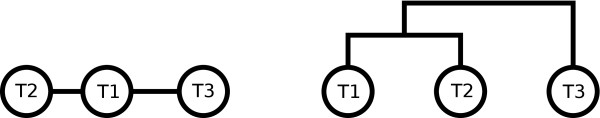
**MSTs and time-series data**. An example with a time-series data set of three samples (T1, T2, and T3) where the dissimilarity between T1 and T2 is the smallest, but between T2 and T3 is the largest. This phenomenon is depicted well by the MST (left), but not by the dendrogram (right) since T1 and T2 are part of the same sub-tree and could be re-ordered depending on the hierarchical clustering implementation.

To emphasize this point, suppose we have a time-course experiment with three samples at time points T1, T2, and T3. If the expression levels are such that T1 and T2 are the most similar, but T2 and T3 are the most dissimilar, then the MST to the left of Figure 18 would be generated. However, hierarchical clustering implementations such as agnes would produce the dendrogram on the right since T1 and T2 are part of the same sub-tree and their relative order is determined by the order of the experiments in the data file. This is not a property specific to agnes since the definition of hierarchical clustering does not give preference to either of the two parts of each sub-tree. It is for this reason that MSTs are more suitable for presenting time-series data.

## Conclusion

In this paper, we have described a system called HAMSTER which allows users to visualize the experiments of a gene expression data set as a *set *of minimum spanning trees (MSTs). In addition, we also describe three scoring schemes which help users judge the quality of these MSTs.

Our results show that MSTs offer a view of microarray data that is related to, but still different from the dendrograms that have been used for data visualization and clustering by others. The creation of a set of MSTs in this manner is absent from previous works with MSTs. This feature allows users to visualize microarray data in terms of dendrograms by presenting relationships between sub-trees. Through examples, we show that MSTs are particularly useful for microarray studies with gradient-based data (such as time-course studies).

The HAMSTER system implements the above procedure as an open-source, GPL licensed software that makes use of other tools, including Graphviz and (optionally) Open MPI. In addition, we also introduced a web server called HAMSTER^+ ^which has been developed to add a wrapper around HAMSTER that is tailored toward NCBI GEO data. HAMSTER^+ ^allows users to evaluate HAMSTER without any local software installation. Both HAMSTER and HAMSTER^+ ^are available from http://hamster.cbrc.jp/. While HAMSTER's original intention was to depict microarray experiments as a set of MSTs, the system is general enough that it could be used directly for probes if the data set is transposed. Evaluation of this potential purpose of HAMSTER is left as future work. Our survey of GEO data has shown that the number of samples in a microarray data set is typically less than 200 (see Table 4). While HAMSTER's running time of 67 seconds for the largest data set seems acceptable, if data sets were many more times this, then parallelization of build-mst using MPI is another possible avenue for future work [20]. It would also be interesting to explore other aspects of HAMSTER unrelated to running time, such as scoring schemes that better reflect the needs of users.

## Abbreviations

MST: minimum spanning tree; HC: hierarchical clustering; HAMSTER: Helpful Abstraction using Minimum Spanning Trees for Expression Relations; MPI: Message Passing Interface; GEO: Gene Expression Omnibus; SOFT: Simple Omnibus Format in Text; PNG: Portable Network Graphics; DPI: dots per inch.

## Competing interests

The authors declare that they have no competing interests.

## Authors' contributions

LK, HH, and PH initially applied MSTs to microarray data. The HAMSTER system was conceived by RW, HM, and PH and implemented by RW. LK manually assigned categories to the GEO used by the HAMSTER server. HAMSTER^+ ^was developed and is maintained by RW and HH. RW, HM, and PH drafted the manuscript. All authors read and approved the final manuscript.

## Supplementary Material

Additional file 1 **HAMSTER - build-mst**. Source code documentation for build-mst generated using Doxygen [24]. Click here for file

Additional file 2 **HAMSTER - layout-mst**. Source code documentation for layout-mst generated using Doxygen [24]. Click here for file
