# Supplementary material for: HAMSTER: visualizing microarray experiments as a set of minimum spanning trees
Source: Source Code Biol Med. 2009 Nov 20;4:8. doi: 10.1186/1751-0473-4-8 (PMC2784758; doi:10.1186/1751-0473-4-8)
Supplement: Additional file 1 — HAMSTER - build-mst. Source code documentation for build-mst generated using Doxygen [24]. [file 1751-0473-4-8-S1.PDF]

Hamster - build-mst

1.2

Generated by Doxygen 1.5.6

Sun Oct 11 18:19:42 2009

Contents

1 Class Index **1**

1.1 Class List 1

2 File Index **3**

2.1 File List 3

3 Class Documentation **5**

3.1 BUILDMST Class Reference 5

3.1.1 Detailed Description 8

3.1.2 Constructor & Destructor Documentation 8

3.1.2.1 BUILDMST 8

3.1.3 Member Function Documentation 8

3.1.3.1 processOptions 8

3.1.3.2 initSettings 9

3.1.3.3 checkSettings 10

3.1.3.4 run 10

3.1.3.5 readMicroarray 11

3.1.3.6 readAttr 12

3.1.3.7 initializeDistances 12

3.1.3.8 initializeClusters 13

3.1.3.9 calculateLinkage 13

3.1.3.10 calculateScores 13

3.1.3.11 normalizeScores 14

3.1.3.12 printScores 14

3.1.3.13 setDebug 14

3.1.3.14 getDebug 14

3.1.3.15 setVerbose 14

3.1.3.16 getVerbose 14

3.1.3.17 setDistance 14

3.1.3.18 getDistance 14
3.1.3.19 setLinkage 14
3.1.3.20 getLinkage 15
3.1.3.21 setScoreMethod 15
3.1.3.22 getScoreMethod 15
3.1.3.23 setCentroid 15
3.1.3.24 getCentroid 15
3.1.3.25 setMicroarrayFn 15
3.1.3.26 getMicroarrayFn 15
3.1.3.27 setAttrFn 15
3.1.3.28 getAttrFn 15
3.1.3.29 setPath 16
3.1.3.30 getPath 16
3.1.3.31 setM 16
3.1.3.32 getM 16
3.1.3.33 setN 16
3.1.3.34 getN 16
3.1.4 Member Data Documentation 16
3.1.4.1 debug_flag 16
3.1.4.2 verbose_flag 16
3.1.4.3 distance 16
3.1.4.4 linkage 16
3.1.4.5 scoring 17
3.1.4.6 centroid 17
3.1.4.7 attr_fn 17
3.1.4.8 microarray_fn 17
3.1.4.9 path 17
3.1.4.10 M 17
3.1.4.11 N 17
3.1.4.12 clusters 17
3.1.4.13 pqueue 17
3.1.4.14 scores 17
3.1.4.15 data 17
3.1.4.16 dist_matrix 18
3.2 CLUSTER Class Reference 19
3.2.1 Detailed Description 21

3.2.2 Constructor & Destructor Documentation 21
3.2.2.1 CLUSTER 21
3.2.2.2 CLUSTER 21
3.2.2.3 CLUSTER 21
3.2.3 Member Function Documentation 22
3.2.3.1 setName 22
3.2.3.2 setColour 22
3.2.3.3 setShape 22
3.2.3.4 getID 22
3.2.3.5 getName 22
3.2.3.6 getColour 22
3.2.3.7 getShape 23
3.2.3.8 getItems 23
3.2.3.9 haveAncestors 23
3.2.3.10 setAncestors 23
3.2.3.11 formCentroid 23
3.2.3.12 getCentroid 23
3.2.3.13 linkSingle 23
3.2.3.14 linkAverage 24
3.2.3.15 linkComplete 24
3.2.3.16 linkCentroid 24
3.2.4 Member Data Documentation 24
3.2.4.1 id 24
3.2.4.2 name 24
3.2.4.3 colour 25
3.2.4.4 shape 25
3.2.4.5 items 25
3.2.4.6 ancestors 25
3.2.4.7 centroid 25
3.3 GRAPH Class Reference 26
3.3.1 Detailed Description 26
3.3.2 Constructor & Destructor Documentation 27
3.3.2.1 GRAPH 27
3.3.3 Member Function Documentation 27
3.3.3.1 printEdges 27
3.3.3.2 printNodes 27

3.3.4 Member Data Documentation 27
3.3.4.1 g 27
3.3.4.2 edges 27
3.3.4.3 weights 27
3.3.4.4 weights_pmap 28
3.3.4.5 spanning_tree_edges 28
3.3.4.6 spanning_tree_vertices 28
3.4 HEAPNODE Class Reference 29
3.4.1 Detailed Description 29
3.4.2 Constructor & Destructor Documentation 30
3.4.2.1 HEAPNODE 30
3.4.2.2 HEAPNODE 30
3.4.3 Member Function Documentation 30
3.4.3.1 operator< 30
3.4.3.2 operator> 30
3.4.3.3 getLeft 30
3.4.3.4 getRight 30
3.4.3.5 getScore 30
3.4.4 Member Data Documentation 30
3.4.4.1 left 30
3.4.4.2 right 30
3.4.4.3 score 31
3.5 SCORE Class Reference 32
3.5.1 Detailed Description 33
3.5.2 Constructor & Destructor Documentation 33
3.5.2.1 SCORE 33
3.5.2.2 SCORE 34
3.5.3 Member Function Documentation 34
3.5.3.1 setID 34
3.5.3.2 setLeft 34
3.5.3.3 setRight 34
3.5.3.4 setScore1 34
3.5.3.5 setScore2 34
3.5.3.6 setCombinedScore 34
3.5.3.7 getID 34
3.5.3.8 getLeft 34

3.5.3.9 getRight 34
3.5.3.10 getScore1 35
3.5.3.11 getScore2 35
3.5.3.12 getCombinedScore 35
3.5.3.13 scoreGaps 35
3.5.3.14 scoreANOVA 35
3.5.3.15 scoreNormalizedAssoc 36
3.5.3.16 scoreNormalizedAssocOrig 36
3.5.4 Member Data Documentation 37
3.5.4.1 id 37
3.5.4.2 left 37
3.5.4.3 right 37
3.5.4.4 score1 37
3.5.4.5 score2 37
3.5.4.6 combined 37
3.6 SPEARMAN Class Reference 38
3.6.1 Detailed Description 39
3.6.2 Constructor & Destructor Documentation 39
3.6.2.1 SPEARMAN 39
3.6.2.2 SPEARMAN 39
3.6.3 Member Function Documentation 39
3.6.3.1 setValue 39
3.6.3.2 setOrigPos 39
3.6.3.3 setRank 39
3.6.3.4 getValue 39
3.6.3.5 getOrigPos 39
3.6.3.6 getRank 39
3.6.3.7 operator< 40
3.6.3.8 copyOrigPosToKey 40
3.6.4 Member Data Documentation 40
3.6.4.1 key 40
3.6.4.2 value 40
3.6.4.3 origpos 40
3.6.4.4 rank 40
3.7 VECT Class Reference 41
3.7.1 Detailed Description 43

3.7.2 Constructor & Destructor Documentation 43
3.7.2.1 VECT 43
3.7.2.2 VECT 43
3.7.2.3 VECT 43
3.7.2.4 VECT 43
3.7.3 Member Function Documentation 44
3.7.3.1 setID 44
3.7.3.2 setName 44
3.7.3.3 setColour 44
3.7.3.4 setShape 44
3.7.3.5 getID 44
3.7.3.6 getName 44
3.7.3.7 getColour 44
3.7.3.8 getShape 44
3.7.3.9 getExpr 45
3.7.3.10 getNull 45
3.7.3.11 isNull 45
3.7.3.12 putExpr 45
3.7.3.13 putNull 45
3.7.3.14 getN 45
3.7.3.15 resize 45
3.7.3.16 simEuc 45
3.7.3.17 simMan 46
3.7.3.18 simPear 46
3.7.3.19 simSpear 46
3.7.4 Member Data Documentation 47
3.7.4.1 id 47
3.7.4.2 name 47
3.7.4.3 colour 47
3.7.4.4 shape 47
3.7.4.5 exprs 47
3.7.4.6 nulls 47

4 File Documentation 49

4.1 build_mst.cpp File Reference 49
4.2 build_mst.h File Reference 50
4.3 calculate.cpp File Reference 51

4.4 check.cpp File Reference . . . 52
4.4.1 Function Documentation . . . 52
4.4.1.1 sanitizeFilename . . . 52
4.4.1.2 sanitizePath . . . 52
4.4.1.3 sanitizeURL . . . 52
4.5 check.h File Reference . . . 53
4.5.1 Function Documentation . . . 53
4.5.1.1 sanitizeFilename . . . 53
4.5.1.2 sanitizePath . . . 53
4.5.1.3 sanitizeURL . . . 53
4.6 cluster.cpp File Reference . . . 54
4.7 cluster.h File Reference . . . 55
4.8 cluster_link.cpp File Reference . . . 56
4.9 global_defn.h File Reference . . . 57
4.9.1 Define Documentation . . . 58
4.9.1.1 CFG_FILENAME . . . 58
4.9.1.2 DEFAULT_COLOUR . . . 58
4.9.1.3 DEFAULT_SHAPE . . . 58
4.9.1.4 EDGES_FILE_EXTENSION . . . 58
4.9.1.5 NODES_FILE_EXTENSION . . . 58
4.9.1.6 NULL_EXPR . . . 58
4.9.1.7 SCORES_FILENAME . . . 58
4.9.1.8 VERBOSE_WIDTH . . . 58
4.9.2 Enumeration Type Documentation . . . 58
4.9.2.1 DIST_METHOD . . . 58
4.9.2.2 LINK_METHOD . . . 59
4.9.2.3 SCORE_METHOD . . . 59
4.10 graph.h File Reference . . . 60
4.10.1 Typedef Documentation . . . 60
4.10.1.1 adjGraph . . . 60
4.10.1.2 Edge . . . 60
4.10.1.3 EdgePair . . . 60
4.10.1.4 Vertex . . . 61
4.11 graph_kruskal.cpp File Reference . . . 62
4.12 heapnode.cpp File Reference . . . 63
4.13 heapnode.h File Reference . . . 64

4.14 io.cpp File Reference . . . 65
4.15 main.cpp File Reference . . . 66
4.15.1 Function Documentation . . . 66
4.15.1.1 main . . . 66
4.16 parameters.cpp File Reference . . . 68
4.17 run.cpp File Reference . . . 69
4.18 score.cpp File Reference . . . 70
4.19 score.h File Reference . . . 71
4.20 vect.cpp File Reference . . . 72
4.21 vect.h File Reference . . . 73
4.22 vect_dist.cpp File Reference . . . 74
4.23 vect_spear.cpp File Reference . . . 75
4.24 vect_spear.h File Reference . . . 76

Chapter 1

Class Index

1.1 Class List

Here are the classes, structs, unions and interfaces with brief descriptions:

BUILDMST 5
CLUSTER 19
GRAPH (Edge weight for undirected graphs) 26
HEAPNODE 29
SCORE 32
SPEARMAN 38
VECT 41

Chapter 2

File Index

2.1 File List

Here is a list of all files with brief descriptions:

build_mst.cpp 49
build_mst.h 50
calculate.cpp 51
check.cpp 52
check.h 53
cluster.cpp 54
cluster.h 55
cluster_link.cpp 56
global_defn.h 57
graph.h 60
graph_kruskal.cpp 62
heapnode.cpp 63
heapnode.h 64
io.cpp 65
main.cpp 66
parameters.cpp 68
run.cpp 69
score.cpp 70
score.h 71
vect.cpp 72
vect.h 73
vect_dist.cpp 74
vect_spear.cpp 75
vect_spear.h 76

Chapter 3

Class Documentation

3.1 BUILDMST Class Reference

`#include <build_mst.h>`

Public Member Functions

- BUILDMST ()

  Constructor that takes no arguments.

- bool processOptions (int argc, char *argv[ ])

  Process options from the command line and the configuration file CFG_FILENAME.

- void initSettings ()

  Initialize settings to provide default values.

- bool checkSettings ()

  Check the settings to ensure they are valid.

- void run ()

  Execute the program after all parameters check out – does the main work of the program.

- bool readMicroarray ()

  Read the microarray data file in.

- bool readAttr ()

  Read the optional attribute file in.

- void initializeDistances ()

  Initialize the distance matrix.

- void initializeClusters ()

  Initialize the clusters with the data file.

- void calculateLinkage (CLUSTER &arg)

Calculate the linkage.

- void calculateScores (SCORE &arg)

  Calculate the graph scores.

- void normalizeScores ()

  Normalize the scores in the scores vector.

- bool printScores (string outpath)

  Print all of the scores out to a text file.

- void setDebug (bool arg)

  Set whether or not debugging output is required.

- bool getDebug () const

  Get the debug setting.

- void setVerbose (bool arg)

  Set whether or not verbose output is required.

- bool getVerbose () const

  Get the verbose setting.

- void setDistance (DIST_METHOD arg)

  Set the distance method.

- DIST_METHOD getDistance () const

  Get the distance setting.

- void setLinkage (LINK_METHOD arg)

  Set the linkage method.

- LINK_METHOD getLinkage () const

  Get the linkage setting.

- void setScoreMethod (SCORE_METHOD arg)

  Set the scoring method.

- SCORE_METHOD getScoreMethod () const

  Get the score method setting.

- void setCentroid (DIST_METHOD arg)

  Set the distance method for centroid linkage.

- DIST_METHOD getCentroid () const

  Get the distance setting for centroid linkage.

- void setMicroarrayFn (string arg)

  Set the microarray filename.

- string getMicroarrayFn () const

  Get the microarray filename.

- void setAttrFn (string arg)

  Set the attribute filename.

- string getAttrFn () const

  Get the attribute filename.

- void setPath (string arg)

  Set the output path (the path where files will be written to).

- string getPath () const

  Get the output path.

- void setM (unsigned int arg)

  Set M – the number of objects (experiments or rows) in the data set.

- unsigned int getM () const

  Get the number of objects in the data set.

- void setN (unsigned int arg)

  Set N – the number of attributes (probes or columns) in the data set.

- unsigned int getN () const

  Get the number of attributes in the data set.

Private Attributes

- bool debug_flag

  Set to true if debug output is required; false otherwise.

- bool verbose_flag

  Set to true if verbose output is required; false otherwise.

- enum DIST_METHOD distance

  Distance method.

- enum LINK_METHOD linkage

  Linkage method.

- enum SCORE_METHOD scoring

  MST scoring method.

- enum DIST_METHOD centroid

  Distance method for centroid linkage.

- string attr_fn

Attribute filename.

- string microarray_fn

  Microarray filename.

- string path

  Output path.

- unsigned int M

  Number of rows.

- unsigned int N

  Number of columns.

- vector< CLUSTER > clusters

  The vector of clusters; grows from M to at most (M + M - 1) entries.

- priority_queue< HEAPNODE, std::vector< HEAPNODE >, greater< HEAPNODE > > pqueue

  The priority queue, implemented as a heap.

- vector< SCORE > scores
- vector< VECT > data

  Original microarray data with each row as an entry in this vector.

- double ∗∗ dist_matrix

  Distance matrix of size (M ∗ M).

3.1.1 Detailed Description

The BUILDMST class is the main class for this program. The main driver (in main.cpp) creates an instance of this class as the first task. It is also the last class destroyed before the program exits.

3.1.2 Constructor & Destructor Documentation

3.1.2.1 BUILDMST::BUILDMST ()

Constructor that takes no arguments.

3.1.3 Member Function Documentation

3.1.3.1 bool BUILDMST::processOptions (int *argc*, char ∗ *argv*[ ])

Process options from the command line and the configuration file CFG_FILENAME.

This function makes use of Boost's program_options for handling arguments on the command line and in options in a configuration file whose format resembles .ini files.

Initially, boolean and enumerated values are given default values. Then, the available options are set up, with default values for string and numeric types. The description of the options are recorded.

Next, the command line options are read, followed by the configuration file options. The command line options take priority over the configuration file ones. Then, the options are processed, one-by-one.

All of this is encapsulated within a try...catch block.

Here is the call graph for this function:

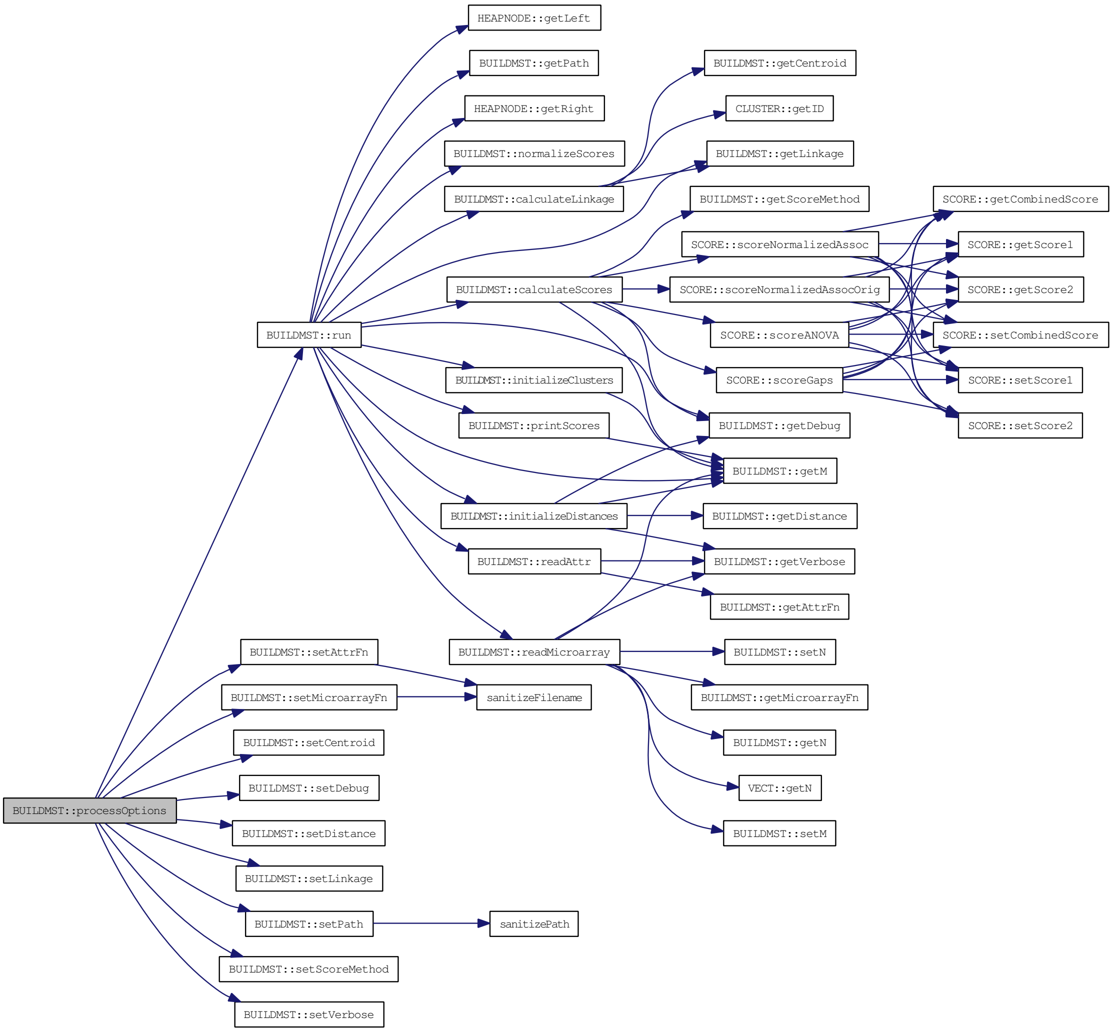

3.1.3.2 void BUILDMST::initSettings ()

Initialize settings to provide default values.

We initialize values which are not initialized by the processOptions function.

Here is the call graph for this function:

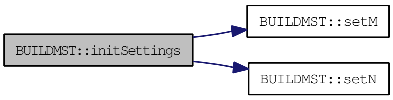

3.1.3.3 bool BUILDMST::checkSettings ()

Check the settings to ensure they are valid.

If no distance or linkage is set, then Euclidean and single linkage are assigned by default. If –verbose has been set, then the options that the user chose are printed out to STDERR.

Here is the call graph for this function:

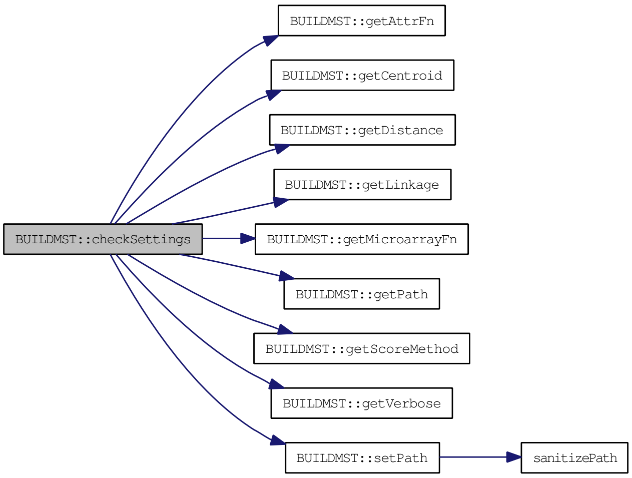

3.1.3.4 void BUILDMST::run ()

Execute the program after all parameters check out – does the main work of the program.

Here is the call graph for this function:

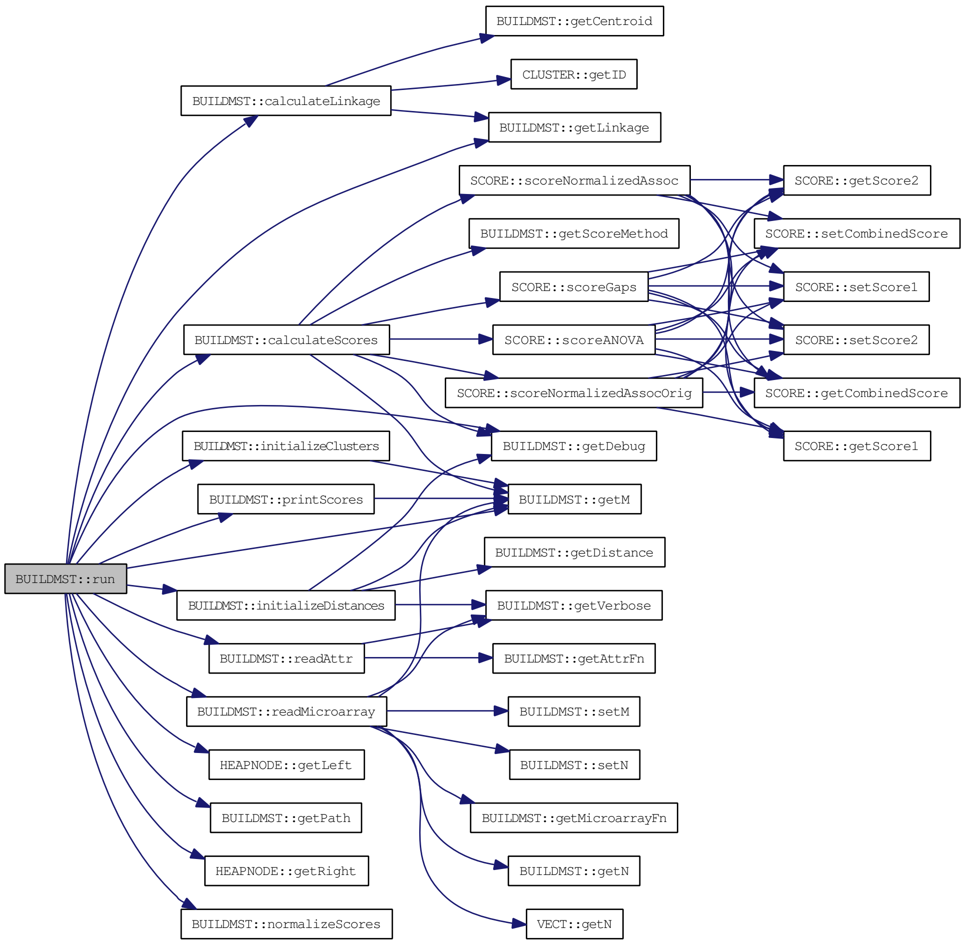

3.1.3.5 bool BUILDMST::readMicroarray ()

Read the microarray data file in.

The data file must be tab-separated with an experiment on each line (assuming the user is building MSTs on the experiments). The first row and column are headers and are basically ignored. All other fields must be either floating point values or the string NULL.

Each row in the data file translates into a VECT object.

Here is the call graph for this function:

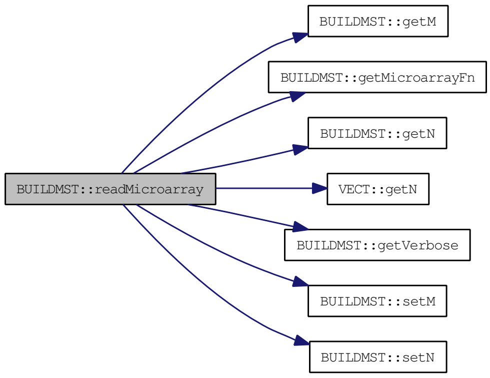

3.1.3.6 bool BUILDMST::readAttr ()

Read the optional attribute file in.

The attribute file is optional. If it is unavailable, then every experiment is assumed to have the default attributes (DEFAULT_COLOUR and DEFAULT_SHAPE, found in buildmst.h). The file is tab-separated with 3 fields on each line: (name, colour, shape).

Here is the call graph for this function:

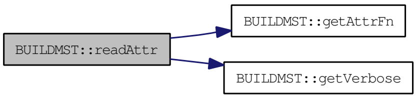

3.1.3.7 void BUILDMST::initializeDistances ()

Initialize the distance matrix.

All of the functions that it calls must be distance (or dissimilarity) functions. That is, a low value (0) indicates highly similar and a high value indicates highly dissimilar.

Distances are stored twice in the matrix – on both sides of the diagonal. As distances are calculated, they are added into the priority queue.

Here is the call graph for this function:

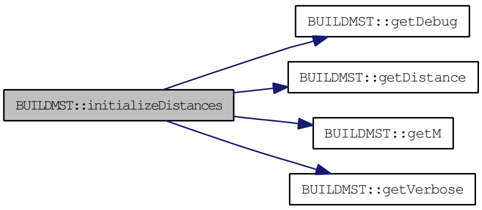

3.1.3.8 void BUILDMST::initializeClusters ()

Initialize the clusters with the data file.

Since bottom-up clustering starts off with each object in its own cluster, this function initializes the clusters vector.

Here is the call graph for this function:

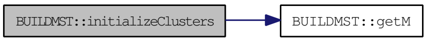

3.1.3.9 void BUILDMST::calculateLinkage (CLUSTER & *arg*)

Calculate the linkage.

For a given cluster, the linkage between it and every other cluster is calculated and added into the priority queue.

Here is the call graph for this function:

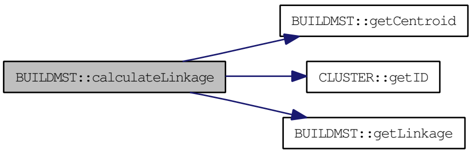

3.1.3.10 void BUILDMST::calculateScores (SCORE & *arg*)

Calculate the graph scores.

The score for the current graph configuration (based on intra and inter-cluster edge weights) is calculated.

Here is the call graph for this function:

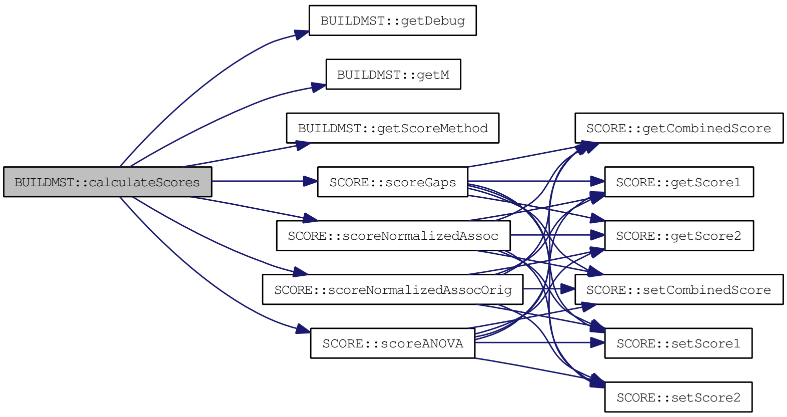

3.1.3.11 void BUILDMST::normalizeScores ()

Normalize the scores in the scores vector.

Make two passes over the scores vector. First, find the maximum combined score. Then divide by this value and multiply by 100 so that they are in the range [0, 100].

If no maximum was found, then everything has a value of 0.0. This indicates something might have gone wrong (i.e., data set too small, etc.), but we set everything to 100 instead.

3.1.3.12 bool BUILDMST::printScores (string *outpath*)

Print all of the scores out to a text file.

Here is the call graph for this function:

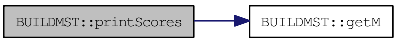

3.1.3.13 void BUILDMST::setDebug (bool *arg*)

Set whether or not debugging output is required.

3.1.3.14 bool BUILDMST::getDebug () const

Get the debug setting.

3.1.3.15 void BUILDMST::setVerbose (bool *arg*)

Set whether or not verbose output is required.

3.1.3.16 bool BUILDMST::getVerbose () const

Get the verbose setting.

3.1.3.17 void BUILDMST::setDistance (DIST_METHOD *arg*)

Set the distance method.

3.1.3.18 DIST_METHOD BUILDMST::getDistance () const

Get the distance setting.

3.1.3.19 void BUILDMST::setLinkage (LINK_METHOD *arg*)

Set the linkage method.

3.1.3.20 LINK_METHOD BUILDMST::getLinkage () const

Get the linkage setting.

3.1.3.21 void BUILDMST::setScoreMethod (SCORE_METHOD *arg*)

Set the scoring method.

3.1.3.22 SCORE_METHOD BUILDMST::getScoreMethod () const

Get the score method setting.

3.1.3.23 void BUILDMST::setCentroid (DIST_METHOD *arg*)

Set the distance method for centroid linkage.

3.1.3.24 DIST_METHOD BUILDMST::getCentroid () const

Get the distance setting for centroid linkage.

3.1.3.25 void BUILDMST::setMicroarrayFn (string *arg*)

Set the microarray filename.

Here is the call graph for this function:

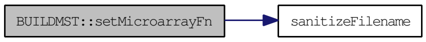

3.1.3.26 string BUILDMST::getMicroarrayFn () const

Get the microarray filename.

3.1.3.27 void BUILDMST::setAttrFn (string *arg*)

Set the attribute filename.

Here is the call graph for this function:

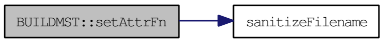

3.1.3.28 string BUILDMST::getAttrFn () const

Get the attribute filename.

3.1.3.29 void BUILDMST::setPath (string *arg*)

Set the output path (the path where files will be written to).

Here is the call graph for this function:

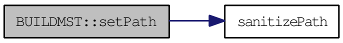

3.1.3.30 string BUILDMST::getPath () const

Get the output path.

3.1.3.31 void BUILDMST::setM (unsigned int *arg*)

Set M – the number of objects (experiments or rows) in the data set.

3.1.3.32 unsigned int BUILDMST::getM () const

Get the number of objects in the data set.

3.1.3.33 void BUILDMST::setN (unsigned int *arg*)

Set N – the number of attributes (probes or columns) in the data set.

3.1.3.34 unsigned int BUILDMST::getN () const

Get the number of attributes in the data set.

3.1.4 Member Data Documentation

3.1.4.1 bool BUILDMST::debug_flag `[private]`

Set to true if debug output is required; false otherwise.

3.1.4.2 bool BUILDMST::verbose_flag `[private]`

Set to true if verbose output is required; false otherwise.

3.1.4.3 enum DIST_METHOD BUILDMST::distance `[private]`

Distance method.

3.1.4.4 enum LINK_METHOD BUILDMST::linkage `[private]`

Linkage method.

3.1.4.5 enum SCORE_METHOD BUILDMST::scoring `[private]`

MST scoring method.

3.1.4.6 enum DIST_METHOD BUILDMST::centroid `[private]`

Distance method for centroid linkage.

3.1.4.7 string BUILDMST::attr_fn `[private]`

Attribute filename.

3.1.4.8 string BUILDMST::microarray_fn `[private]`

Microarray filename.

3.1.4.9 string BUILDMST::path `[private]`

Output path.

3.1.4.10 unsigned int BUILDMST::M `[private]`

Number of rows.

3.1.4.11 unsigned int BUILDMST::N `[private]`

Number of columns.

3.1.4.12 vector<CLUSTER> BUILDMST::clusters `[private]`

The vector of clusters; grows from M to at most (M + M - 1) entries.

3.1.4.13 priority_queue<HEAPNODE, std::vector<HEAPNODE>, greater<HEAPNODE> > BUILDMST::pqueue `[private]`

The priority queue, implemented as a heap.

3.1.4.14 vector<SCORE> BUILDMST::scores `[private]`

The vector of scores; each position in this array represents the score of the MST from one merge step.

3.1.4.15 vector<VECT> BUILDMST::data `[private]`

Original microarray data with each row as an entry in this vector.

3.1.4.16 double** BUILDMST::dist_matrix [private]

Distance matrix of size (M * M).

The documentation for this class was generated from the following files:

- build_mst.h
- build_mst.cpp
- calculate.cpp
- io.cpp
- parameters.cpp
- run.cpp

3.2 CLUSTER Class Reference

`#include <cluster.h>`

Collaboration diagram for CLUSTER:

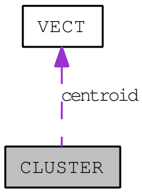

Public Member Functions

- CLUSTER ()

  Default constructor; should never be called (not needed).

- CLUSTER (unsigned int arg_id, string arg1, string arg2, string arg3, VECT arg4)

  Constructor for creating the initial clusters.

- CLUSTER (unsigned int arg_id, CLUSTER *arg1, CLUSTER *arg2, LINK_METHOD arg3, unsigned int arg4)

  Constructor for merging two clusters.

- void setName (string arg)

  Set the cluster name.

- void setColour (string arg)

  Set the cluster colour.

- void setShape (string arg)

  Set the cluster shape.

- unsigned int getID () const

  Get the cluster ID.

- string getName () const

  Get the cluster name.

- string getColour () const

  Get the cluster colour.

- string getShape () const

  Get the cluster shape.

- vector< unsigned int > getItems () const

  Get the components that make up a cluster.

- bool haveAncestors ()
- void setAncestors ()

  Set the ancestors variable to TRUE.

- void formCentroid (CLUSTER ∗a, CLUSTER ∗b)

  Create a centroid vector from two clusters.

- VECT getCentroid () const

  Get the centroid vector from this cluster.

- double linkSingle (CLUSTER ∗other, double ∗∗d)

  The single linkage between this cluster and another one.

- double linkAverage (CLUSTER ∗other, double ∗∗d)

  The average linkage between this cluster and another one.

- double linkComplete (CLUSTER ∗other, double ∗∗d)

  The complete linkage between this cluster and another one.

- double linkCentroid (CLUSTER ∗other, vector< VECT > ∗data, enum DIST_METHOD distance)

  The centroid linkage between this cluster and another one.

Private Attributes

- unsigned int id

  Numerical ID for this cluster.

- string name

  The name of this cluster; essentially the string representation of the id.

- string colour

  The colour of this cluster when drawn.

- string shape

  The shape of this cluster when drawn.

- vector< unsigned int > items

  The experiments (components) that make up this cluster.

- bool ancestors

  Does this cluster have an ancestor?

- VECT centroid

  The vector that represents the centroid of this cluster.

3.2.1 Detailed Description

The CLUSTER class represents a set of one or more experiments. Like experiments, it has attributes like a name, colour, and shape. These attributes are inherited from the experiments that form the cluster. Colours and shapes are their default values if the experiments that make it up are a mix of different attributes. The name is simply a string representation of their integral IDs.

Clusters also have a vector of the experiments that make it up. We call them "items". The vector of items has no order.

If a cluster has an ancestor (ancestor = TRUE), then that means it is contained within a larger cluster. When this program completes, every cluster has an ancestor except for the very last one.

Clusters are formed using constructors from either a single experiment or from two clusters.

Since linkages are calculated between clusters, their functions are part of this class.

3.2.2 Constructor & Destructor Documentation

3.2.2.1 CLUSTER::CLUSTER ()

Default constructor; should never be called (not needed).

3.2.2.2 CLUSTER::CLUSTER (unsigned int *arg_id*, string *arg1*, string *arg2*, string *arg3*, VECT *arg4*)

Constructor for creating the initial clusters.

Parameters:

arg_id The integral ID for this cluster

arg1 The name of this cluster

arg2 The colour of this cluster

arg3 The shape of this cluster

arg4 The VECT object for this cluster of one item

3.2.2.3 CLUSTER::CLUSTER (unsigned int *arg_id*, CLUSTER ∗ *arg1*, CLUSTER ∗ *arg2*, LINK_METHOD *arg3*, unsigned int *arg4*)

Constructor for merging two clusters.

Parameters:

arg_id The integral ID for this cluster

arg1 The first cluster

arg2 The second cluster

arg3 The linkage method being used

arg4 Number of experiments in microarray (for naming)

This constructor sets the attributes of the cluster and if centroid linkage is used, calls formCentroid () to build a centroid vector.

Note: There is no order to clusters so arg1 and arg2 can be swapped.

Here is the call graph for this function:

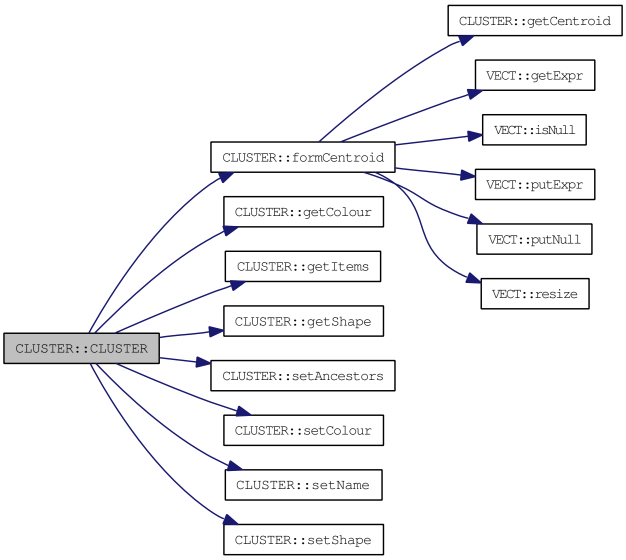

3.2.3 Member Function Documentation

3.2.3.1 void CLUSTER::setName (string *arg*)

Set the cluster name.

3.2.3.2 void CLUSTER::setColour (string *arg*)

Set the cluster colour.

3.2.3.3 void CLUSTER::setShape (string *arg*)

Set the cluster shape.

3.2.3.4 unsigned int CLUSTER::getID () const

Get the cluster ID.

3.2.3.5 string CLUSTER::getName () const

Get the cluster name.

3.2.3.6 string CLUSTER::getColour () const

Get the cluster colour.

3.2.3.7 string CLUSTER::getShape () const

Get the cluster shape.

3.2.3.8 vector< unsigned int > CLUSTER::getItems () const

Get the components that make up a cluster.

3.2.3.9 bool CLUSTER::haveAncestors () [inline]

3.2.3.10 void CLUSTER::setAncestors ()

Set the ancestors variable to TRUE.

3.2.3.11 void CLUSTER::formCentroid (CLUSTER * *a*, CLUSTER * *b*)

Create a centroid vector from two clusters.

The centroid vector is formed by averaging across each feature of the vector. If the value of position i in any vector is NULL, then position i of the new column is automatically NULL such that the sum of any value with NULL is NULL. The centroid vector is stored as a VECT object.

Here is the call graph for this function:

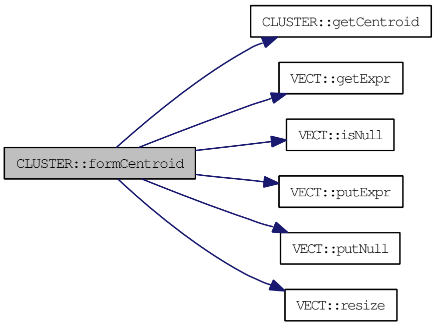

3.2.3.12 VECT CLUSTER::getCentroid () const

Get the centroid vector from this cluster.

3.2.3.13 double CLUSTER::linkSingle (CLUSTER * *other*, double ** *d*)

The single linkage between this cluster and another one.

Here is the call graph for this function:

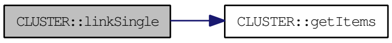

3.2.3.14 double CLUSTER::linkAverage (CLUSTER * *other*, double ** *d*)

The average linkage between this cluster and another one.

Here is the call graph for this function:

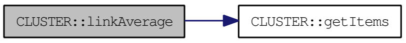

3.2.3.15 double CLUSTER::linkComplete (CLUSTER * *other*, double ** *d*)

The complete linkage between this cluster and another one.

Here is the call graph for this function:

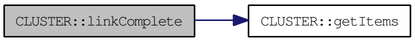

3.2.3.16 double CLUSTER::linkCentroid (CLUSTER * *other*, vector< VECT > * *data*, enum DIST_METHOD *distance*)

The centroid linkage between this cluster and another one.

The dissimilarity between two centroid vectors depends on the distance parameter chosen by the user. That is, the Euclidean distance is not always used. Always using the Euclidean distance may be preferred, but that is up to the user.

Here is the call graph for this function:

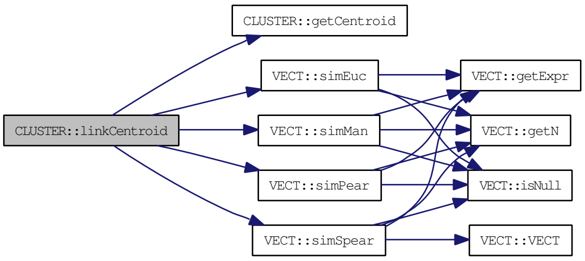

3.2.4 Member Data Documentation

3.2.4.1 unsigned int CLUSTER::id [private]

Numerical ID for this cluster.

3.2.4.2 string CLUSTER::name [private]

The name of this cluster; essentially the string representation of the id.

3.2.4.3 string CLUSTER::colour [private]

The colour of this cluster when drawn.

3.2.4.4 string CLUSTER::shape [private]

The shape of this cluster when drawn.

3.2.4.5 vector<unsigned int> CLUSTER::items [private]

The experiments (components) that make up this cluster.

3.2.4.6 bool CLUSTER::ancestors [private]

Does this cluster have an ancestor?

i.e., if set to TRUE, then a larger cluster has been formed that includes this cluster; default value FALSE for all clusters.

3.2.4.7 VECT CLUSTER::centroid [private]

The vector that represents the centroid of this cluster.

The documentation for this class was generated from the following files:

- cluster.h
- cluster.cpp
- cluster_link.cpp

3.3 GRAPH Class Reference

Edge weight for undirected graphs.

```
#include <graph.h>
```

Public Member Functions

- GRAPH (unsigned int M, priority_queue< HEAPNODE, std::vector< HEAPNODE >, greater< HEAPNODE > > pqueue, vector< CLUSTER > clusters)

  Constructor for GRAPH object with three parameters.

- void printEdges (unsigned int id, vector< CLUSTER > clusters, string outpath)

  Print the edges of the MST out to file.

- void printNodes (unsigned int id, vector< CLUSTER > clusters, string outpath)

  Print the nodes of the MST out to file.

Private Attributes

- adjGraph * g

  The undirected graph represented as an adjacency list.

- vector< EdgePair > edges

  The set of edges.

- vector< double > weights

  The set of edge weights.

- boost::property_map< adjGraph, boost::edge_weight_t >::type weights_pmap

  A property map holding the edge weights for the graph.

- vector< Edge > spanning_tree_edges

  The MST as a vector of edges.

- vector< Vertex > spanning_tree_vertices

  The MST as a vector of vertices [unused].

3.3.1 Detailed Description

Edge weight for undirected graphs.

A GRAPH object includes an undirected graph represented as an adjacency list and the corresponding MST representation.

Edges and edge weights are kept in two separate vectors in a one-to-one relationship such that the weight of edge i is in the weights vector at position i.

In addition to creating the original graph and its corresponding MST, there are functions for printing the graph and its nodes to separate files.

3.3.2 Constructor & Destructor Documentation

3.3.2.1 GRAPH::GRAPH (unsigned int *M*, priority_queue< HEAPNODE, std::vector< HEAPNODE >, greater< HEAPNODE > > *pqueue*, vector< CLUSTER > *clusters*)

Constructor for GRAPH object with three parameters.

Parameters:

- ***M*** Number of experiments (rows) for the current graph (decreases by 1 with each iteration)
- ***pqueue*** Priority queue of potential clusters
- ***clusters*** Vector of clusters

The constructor builds the graph and then calculates the MST using Boost Graph Library's implementation of Kruskal's algorithm.

Here is the call graph for this function:

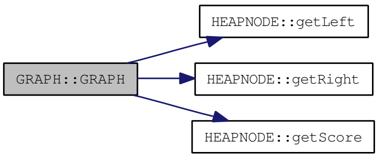

3.3.3 Member Function Documentation

3.3.3.1 void GRAPH::printEdges (unsigned int *id*, vector< CLUSTER > *clusters*, string *outpath*)

Print the edges of the MST out to file.

3.3.3.2 void GRAPH::printNodes (unsigned int *id*, vector< CLUSTER > *clusters*, string *outpath*)

Print the nodes of the MST out to file.

3.3.4 Member Data Documentation

3.3.4.1 adjGraph* GRAPH::g `[private]`

The undirected graph represented as an adjacency list.

3.3.4.2 vector<EdgePair> GRAPH::edges `[private]`

The set of edges.

3.3.4.3 vector<double> GRAPH::weights `[private]`

The set of edge weights.

3.3.4.4 boost::property_map< adjGraph, boost::edge_weight_t >::type GRAPH::weights_pmap [private]

A property map holding the edge weights for the graph.

3.3.4.5 vector< Edge > GRAPH::spanning_tree_edges [private]

The MST as a vector of edges.

3.3.4.6 vector< Vertex > GRAPH::spanning_tree_vertices [private]

The MST as a vector of vertices [unused].

The documentation for this class was generated from the following files:

- graph.h
- graph_kruskal.cpp

3.4 HEAPNODE Class Reference

```
#include <heapnode.h>
```

Public Member Functions

- HEAPNODE ()

  Default HEAPNODE constructor; should never be called (not needed).

- HEAPNODE (unsigned int arg1, unsigned int arg2, double arg3)

  Constructor for a HEAPNODE with three arguments.

- bool operator< (const HEAPNODE &arg) const

  Overloaded operator for HEAPNODEs (less than).

- bool operator> (const HEAPNODE &arg) const

  Overloaded operator for HEAPNODEs (greater than).

- unsigned int getLeft () const

  Get the left child.

- unsigned int getRight () const

  Get the right child.

- double getScore () const

  Get the score.

Private Attributes

- unsigned int left

  The left child.

- unsigned int right

  The right child.

- double score

  The score indicating the dissimilarity between the two children.

3.4.1 Detailed Description

HEAPNODEs represent potential clusters that are formed from two existing clusters. We call these existing clusters "children". One child is the "left" child and the other child is the "right" child. Of course, there is no meaning to this labeling since there is no notion of "left" or "right".

A HEAPNODE becomes an actual cluster if (a) it appears at the top of the priority queue, and (b) both of its child clusters have not already been used to form another cluster earlier in the agglomeration process.

3.4.2 Constructor & Destructor Documentation

3.4.2.1 HEAPNODE::HEAPNODE ()

Default HEAPNODE constructor; should never be called (not needed).

3.4.2.2 HEAPNODE::HEAPNODE (unsigned int *arg1*, unsigned int *arg2*, double *arg3*)

Constructor for a HEAPNODE with three arguments.

Parameters:

arg1 The left child

arg2 The right child

arg3 The score

3.4.3 Member Function Documentation

3.4.3.1 bool HEAPNODE::operator< (const HEAPNODE & *arg*) const

Overloaded operator for HEAPNODEs (less than).

3.4.3.2 bool HEAPNODE::operator> (const HEAPNODE & *arg*) const

Overloaded operator for HEAPNODEs (greater than).

3.4.3.3 unsigned int HEAPNODE::getLeft () const

Get the left child.

3.4.3.4 unsigned int HEAPNODE::getRight () const

Get the right child.

3.4.3.5 double HEAPNODE::getScore () const

Get the score.

3.4.4 Member Data Documentation

3.4.4.1 unsigned int HEAPNODE::left `[private]`

The left child.

3.4.4.2 unsigned int HEAPNODE::right `[private]`

The right child.

3.4.4.3 double HEAPNODE::score [private]

The score indicating the dissimilarity between the two children.

The documentation for this class was generated from the following files:

- heapnode.h
- heapnode.cpp

3.5 SCORE Class Reference

```
#include <score.h>
```

Public Member Functions

- SCORE ()

  Constructor that takes no arguments.

- SCORE (unsigned int arg1, unsigned int arg2, unsigned int arg3)

  Constructor that takes three arguments.

- void setID (unsigned int arg)

  Set the ID.

- void setLeft (unsigned int arg)

  Set the ID of the left cluster.

- void setRight (unsigned int arg)

  Set the ID of the right cluster associated with this score.

- void setScore1 (double arg)

  Set score 1.

- void setScore2 (double arg)

  Set score 2.

- void setCombinedScore (double arg)

  Set combined score.

- unsigned int getID () const

  Get the ID.

- unsigned int getLeft () const

  Get the ID of the left cluster.

- unsigned int getRight () const

  Get the ID of the right cluster.

- double getScore1 () const

  Get score 1.

- double getScore2 () const

  Get score 2.

- double getCombinedScore () const

  Get combined score.

- void scoreGaps (unsigned int M, vector< CLUSTER > *clusters, double **d, bool debug)

Calculate the MST score based on the size of the gap between the largest intra-cluster weight and the smallest inter-cluster weight.

- void scoreANOVA (unsigned int M, vector< CLUSTER > *clusters, double **d, bool debug)

  Calculate the MST score based on the ANOVA of the two groups.

- void scoreNormalizedAssoc (unsigned int M, vector< CLUSTER > *clusters, double **d, bool debug)

  Calculate the MST score based on the normalized association of Shi and Malik (2000).

- void scoreNormalizedAssocOrig (unsigned int M, vector< CLUSTER > *clusters, double **d, bool debug)

  Calculate the MST score based on the normalized association of Shi and Malik (2000).

Private Attributes

- unsigned int id

  The merge ID, numbered from 0.

- unsigned int left

  The left cluster in the merge.

- unsigned int right

  The right cluster in the merge.

- double score1

  The intra-cluster score (within-cluster) or the mean square for groups.

- double score2

  The inter-cluster score (between-cluster) or the mean square error.

- double combined

  The combined score calculated by either subtracting or dividing score1 and score2.

3.5.1 Detailed Description

A SCORE node keeps track of the intra and inter-cluster scores for a particular merge. In addition to these scores, information about the merge (its unique integral ID and the IDs of the two clusters that were merged) are also kept track of

3.5.2 Constructor & Destructor Documentation

3.5.2.1 SCORE::SCORE ()

Constructor that takes no arguments.

3.5.2.2 SCORE::SCORE (unsigned int *arg1*, unsigned int *arg2*, unsigned int *arg3*)

Constructor that takes three arguments.

Parameters:

- ***arg1*** ID of the node
- ***arg2*** ID of the left cluster
- ***arg3*** ID of the right cluster

3.5.3 Member Function Documentation

3.5.3.1 void SCORE::setID (unsigned int *arg*)

Set the ID.

3.5.3.2 void SCORE::setLeft (unsigned int *arg*)

Set the ID of the left cluster.

3.5.3.3 void SCORE::setRight (unsigned int *arg*)

Set the ID of the right cluster associated with this score.

3.5.3.4 void SCORE::setScore1 (double *arg*)

Set score 1.

3.5.3.5 void SCORE::setScore2 (double *arg*)

Set score 2.

3.5.3.6 void SCORE::setCombinedScore (double *arg*)

Set combined score.

3.5.3.7 unsigned int SCORE::getID () const

Get the ID.

3.5.3.8 unsigned int SCORE::getLeft () const

Get the ID of the left cluster.

3.5.3.9 unsigned int SCORE::getRight () const

Get the ID of the right cluster.

3.5.3.10 double SCORE::getScore1 () const

Get score 1.

3.5.3.11 double SCORE::getScore2 () const

Get score 2.

3.5.3.12 double SCORE::getCombinedScore () const

Get combined score.

3.5.3.13 void SCORE::scoreGaps (unsigned int *M*, vector< CLUSTER > $*$ *clusters*, double $**$ *d*, bool *debug*)

Calculate the MST score based on the size of the gap between the largest intra-cluster weight and the smallest inter-cluster weight.

The main data structure is a matrix called distance_to_cluster which maps a distance at d[i][j] to the cluster it is in; the value UINT_MAX is used to indicate it is not in a cluster (i.e., it is an inter-cluster relationship).

After constructing this data structure, it is used to separate all distances into those that are within a cluster and those that are not. As clustering is performed by increasing distances, we seek to find the size of the gap (in distance) between the largest intra-cluster distance and the smallest inter-cluster distance. Once this is found, we set it and take the absolute value of their difference.

Here is the call graph for this function:

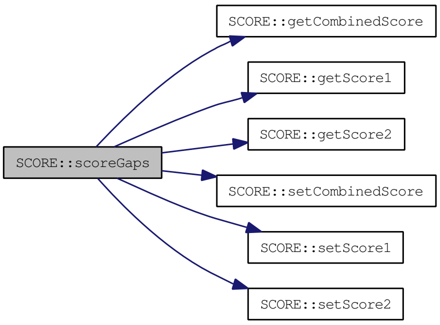

3.5.3.14 void SCORE::scoreANOVA (unsigned int *M*, vector< CLUSTER > $*$ *clusters*, double $**$ *d*, bool *debug*)

Calculate the MST score based on the ANOVA of the two groups.

The distances between experiments are separated into two groups (intra-cluster and inter-cluster) and their sum of squares of both are calculated against the global mean. That is, the mean is unchanged with each application of this function – it is simply the mean of all the values in the distance matrix.

The main data structure is a matrix called distance_to_cluster (see SCORE::scoreGaps).

A vector of intra- and inter-cluster scores are kept since we make two passes over the data (the first to get the mean; the second to calculate the sums of squares).

Here is the call graph for this function:

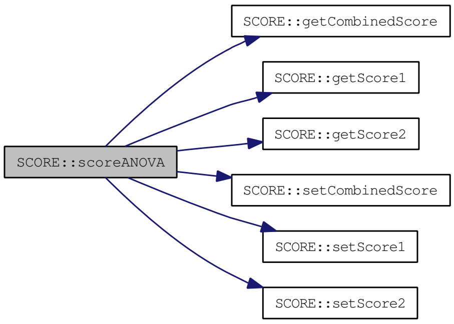

3.5.3.15 void SCORE::scoreNormalizedAssoc (unsigned int *M*, vector< CLUSTER > * *clusters*, double ** *d*, bool *debug*)

Calculate the MST score based on the normalized association of Shi and Malik (2000).

Defn: Assoc (A, A) is the sum of all weights for all distances within cluster A; Assoc (A, V) is the sum of all weights for all distances from nodes in A with all other nodes. If A is a cluster with only one node, then Assoc (A, A) = 0 since there are no self-loops.

The main data structure is a matrix called distance_to_cluster (see SCORE::scoreGaps).

This method performs the following steps: (1) Build distance_to_cluster; (2) Calculate the self-association, Assoc (A, A), for each cluster; (3) Calculate the all-association, Assoc (A, V), for each cluster; (4) Accumulate the scores for all clusters that were "valid" [valid clusters are top-level clusters with no ancestors]; (5) Adjust the scores by multiplying it by the number of clusters; (6) Free memory and store values.

Step (5) is not part of the definition of normalized association of Shi and Malik (2000).

Here is the call graph for this function:

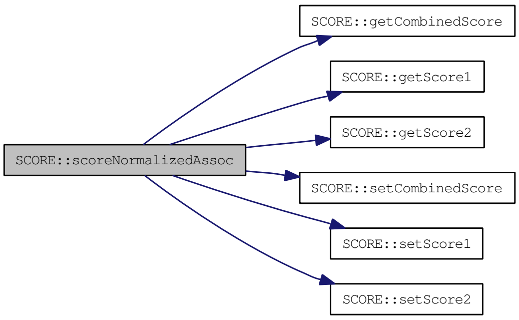

3.5.3.16 void SCORE::scoreNormalizedAssocOrig (unsigned int *M*, vector< CLUSTER > * *clusters*, double ** *d*, bool *debug*)

Calculate the MST score based on the normalized association of Shi and Malik (2000).

See SCORE::scoreNormalizedAssocOrig for a description. Only difference is that the score is not multiplied by the number of clusters.

Here is the call graph for this function:

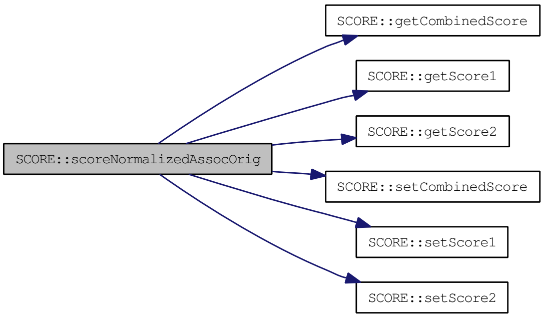

3.5.4 Member Data Documentation

3.5.4.1 unsigned int SCORE::id `[private]`

The merge ID, numbered from 0.

3.5.4.2 unsigned int SCORE::left `[private]`

The left cluster in the merge.

3.5.4.3 unsigned int SCORE::right `[private]`

The right cluster in the merge.

3.5.4.4 double SCORE::score1 `[private]`

The intra-cluster score (within-cluster) or the mean square for groups.

3.5.4.5 double SCORE::score2 `[private]`

The inter-cluster score (between-cluster) or the mean square error.

3.5.4.6 double SCORE::combined `[private]`

The combined score calculated by either subtracting or dividing score1 and score2.

The documentation for this class was generated from the following files:

- score.h
- score.cpp

3.6 SPEARMAN Class Reference

```
#include <vect_spear.h>
```

Public Member Functions

- SPEARMAN ()
  Constructor that takes no arguments.
- SPEARMAN (double v, unsigned int p, double r)
  Constructor that takes three arguments (key/value, original position, rank).
- void setValue (double v)
  Set the value.
- void setOrigPos (unsigned int p)
  Set the original position.
- void setRank (double r)
  Set the rank.
- double getValue () const
  Get the value.
- unsigned int getOrigPos () const
  Get the original position.
- double getRank () const
  Get the rank.
- bool operator< (const SPEARMAN &arg) const
  Overloaded operator for SPEARMAN nodes (less than).
- void copyOrigPosToKey ()
  Copy the original position to the key.

Private Attributes

- double key
  The sort key (either the expression value or the original position in the vector).
- double value
  The expression level of the node.
- unsigned int origpos
  The original position in the vector.
- double rank
  The rank of node within the vector.

3.6.1 Detailed Description

A SPEARMAN node is used to keep track of the expression value, original rank (position), and final, sorted rank.

The nodes are sorted based on the value in the key. For example, if we are sorting based on the expression values, then they are copied to the key and a sort function is applied to the array.

3.6.2 Constructor & Destructor Documentation

3.6.2.1 SPEARMAN::SPEARMAN ()

Constructor that takes no arguments.

3.6.2.2 SPEARMAN::SPEARMAN (double *v*, unsigned int *p*, double *r*)

Constructor that takes three arguments (key/value, original position, rank).

Parameters:

- ***v*** The key (the value we sort on) or the value of the node (i.e., the expression level)
- ***p*** The original position in the vector
- ***r*** The rank of this node respective to the other values

3.6.3 Member Function Documentation

3.6.3.1 void SPEARMAN::setValue (double *v*)

Set the value.

3.6.3.2 void SPEARMAN::setOrigPos (unsigned int *p*)

Set the original position.

3.6.3.3 void SPEARMAN::setRank (double *r*)

Set the rank.

3.6.3.4 double SPEARMAN::getValue () const

Get the value.

3.6.3.5 unsigned int SPEARMAN::getOrigPos () const

Get the original position.

3.6.3.6 double SPEARMAN::getRank () const

Get the rank.

3.6.3.7 bool SPEARMAN::operator< (const SPEARMAN & *arg*) const

Overloaded operator for SPEARMAN nodes (less than).

3.6.3.8 void SPEARMAN::copyOrigPosToKey ()

Copy the original position to the key.

3.6.4 Member Data Documentation

3.6.4.1 double SPEARMAN::key `[private]`

The sort key (either the expression value or the original position in the vector).

3.6.4.2 double SPEARMAN::value `[private]`

The expression level of the node.

3.6.4.3 unsigned int SPEARMAN::origpos `[private]`

The original position in the vector.

3.6.4.4 double SPEARMAN::rank `[private]`

The rank of node within the vector.

The documentation for this class was generated from the following files:

- vect_spear.h
- vect_spear.cpp

3.7 VECT Class Reference

```
#include <vect.h>
```

Public Member Functions

- VECT ()

    Default constructor that takes no arguments.

- VECT (unsigned int arg1, string arg2)

    Constructor that takes two arguments.

- VECT (vector< SPEARMAN > values)

    Constructor that takes a single argument.

- VECT (const VECT &src)

    Copy constructor for a VECT object.

- void setID (unsigned int arg)

    Set the ID.

- void setName (string arg)

    Set the name.

- void setColour (string arg)

    Set the colour.

- void setShape (string arg)

    Set the shape.

- unsigned int getID () const

    Get the ID.

- string getName () const

    Get the name.

- string getColour () const

    Get the colour.

- string getShape () const

    Get the shape.

- double getExpr (unsigned int i) const

    Get the expression level at position i.

- bool getNull (unsigned int i) const

    Get the NULL flag at position i.

- bool isNull (unsigned int i)

Test if the expression level in position i is NULL.

- void putExpr (unsigned int pos, double value)

  Put the expression level at position i.

- void putNull (unsigned int pos, bool value)

  Put the NULL value (true or false) at position i.

- unsigned int getN () const

  Get the size of the vector.

- void resize (unsigned int arg)

  Resize the vector.

- double simEuc (VECT ∗other)

  The Euclidean distance between this vector and another one.

- double simMan (VECT ∗other)

  The Manhattan distance between this vector and another one.

- double simPear (VECT ∗other)

  The Pearson correlation coefficient (distance) between this vector and another one.

- double simSpear (VECT ∗other)

  The Spearman rank correlation coefficient (distance) between this vector and another one.

Private Attributes

- unsigned int id

  ID of this vector (or row).

- string name

  Name of the experiment corresponding to this vector.

- string colour

  Colour of the experiment corresponding to this vector.

- string shape

  Shape of the experiment corresponding to this vector.

- vector< double > exprs

  Vector of expression levels.

- vector< bool > nulls

  Vector of NULL levels as a boolean flag (TRUE = NULL expression level).

3.7.1 Detailed Description

The VECT class represents a vector (or row) in a microarray data file. Each instance has attributes such as a name, colour, and shape. Also, they have a vector of expression levels and a vector of null values. The lengths of these two vectors are identical and match one-to-one. That is, if the value in position (column) i is NULL, then the expression level is a 0 (and should not be used).

Since dissimilarity functions operate between two vectors, these functions are part of this class but are in their own file.

3.7.2 Constructor & Destructor Documentation

3.7.2.1 VECT::VECT ()

Default constructor that takes no arguments.

3.7.2.2 VECT::VECT (unsigned int *arg1*, string *arg2*)

Constructor that takes two arguments.

Parameters:

arg1 ID of the vector

arg2 The row from the microarray data file, represented as a string

Here is the call graph for this function:

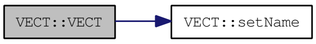

3.7.2.3 VECT::VECT (vector< SPEARMAN > *values*)

Constructor that takes a single argument.

Parameters:

values A vector of SPEARMAN nodes

This constructor copies the values in the SPEARMAN nodes into a VECT object. It assumes that there are NULL values since every value is a rank. NULL values have an expression level of NULL_EXPR and would have all been pushed to one side.

3.7.2.4 VECT::VECT (const VECT & *src*)

Copy constructor for a VECT object.

Parameters:

src The source that we are copying from

This function is used to copy the two vectors an element at a time. The function receives a reference to prevent infinite recursion.

Here is the call graph for this function:

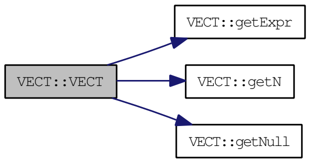

3.7.3 Member Function Documentation

3.7.3.1 void VECT::setID (unsigned int *arg*)

Set the ID.

3.7.3.2 void VECT::setName (string *arg*)

Set the name.

3.7.3.3 void VECT::setColour (string *arg*)

Set the colour.

3.7.3.4 void VECT::setShape (string *arg*)

Set the shape.

3.7.3.5 unsigned int VECT::getID () const

Get the ID.

3.7.3.6 string VECT::getName () const

Get the name.

3.7.3.7 string VECT::getColour () const

Get the colour.

3.7.3.8 string VECT::getShape () const

Get the shape.

3.7.3.9 double VECT::getExpr (unsigned int *i*) const [inline]

Get the expression level at position i.

3.7.3.10 bool VECT::getNull (unsigned int *i*) const [inline]

Get the NULL flag at position i.

3.7.3.11 bool VECT::isNull (unsigned int *i*) [inline]

Test if the expression level in position i is NULL.

3.7.3.12 void VECT::putExpr (unsigned int *pos*, double *value*) [inline]

Put the expression level at position i.

3.7.3.13 void VECT::putNull (unsigned int *pos*, bool *value*) [inline]

Put the NULL value (true or false) at position i.

3.7.3.14 unsigned int VECT::getN () const [inline]

Get the size of the vector.

Note that since exprs and nulls are the same size, we could have also returned the size of exprs.

3.7.3.15 void VECT::resize (unsigned int *arg*)

Resize the vector.

In order to resize the VECT object, we have to keep the lengths of both exprs and nulls the same at all times; so we change both simultaneously here.

3.7.3.16 double VECT::simEuc (VECT * *other*)

The Euclidean distance between this vector and another one.

Function exits if the two vectors are of different dimensions.

Here is the call graph for this function:

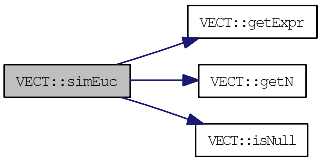

3.7.3.17 double VECT::simMan (VECT * *other*)

The Manhattan distance between this vector and another one.

Function exits if the two vectors are of different dimensions.

Here is the call graph for this function:

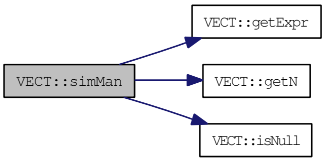

3.7.3.18 double VECT::simPear (VECT * *other*)

The Pearson correlation coefficient (distance) between this vector and another one.

The Pearson correlation coefficient (r) is subtracted from 2 to obtain a distance whose range is [0, 2] such that 0 means two vectors are highly correlated.

Function exits if the two vectors are of different dimensions.

Note: The calculation makes use of the population standard deviation.

Here is the call graph for this function:

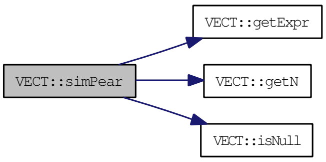

3.7.3.19 double VECT::simSpear (VECT * *other*)

The Spearman rank correlation coefficient (distance) between this vector and another one.

The Spearman rank correlation coefficient is calculated by sorting the two vectors by value, enumerating them separately (i.e., assign ranks), and then returning them to their original order. These ranks are passed to the simPear function.

Function exits if the two vectors are of different dimensions.

The final result is a distance whose range is [0, 2] such that 0 means two vectors are highly correlated.

Here is the call graph for this function:

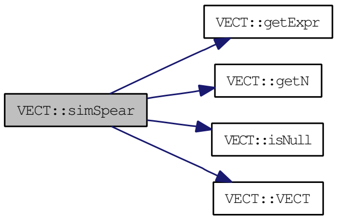

3.7.4 Member Data Documentation

3.7.4.1 unsigned int VECT::id [private]

ID of this vector (or row).

3.7.4.2 string VECT::name [private]

Name of the experiment corresponding to this vector.

3.7.4.3 string VECT::colour [private]

Colour of the experiment corresponding to this vector.

3.7.4.4 string VECT::shape [private]

Shape of the experiment corresponding to this vector.

3.7.4.5 vector<double> VECT::exprs [private]

Vector of expression levels.

3.7.4.6 vector<bool> VECT::nulls [private]

Vector of NULL levels as a boolean flag (TRUE = NULL expression level).

The documentation for this class was generated from the following files:

- vect.h
- vect.cpp
- vect_dist.cpp

Chapter 4

File Documentation

4.1 build_mst.cpp File Reference

```
#include <iostream>
#include <string>
#include <vector>
#include <queue>
#include <cstdlib>
#include "global_defn.h"
#include "check.h"
#include "heapnode.h"
#include "vect_spear.h"
#include "vect.h"
#include "cluster.h"
#include "score.h"
#include "build_mst.h"
```

Include dependency graph for build_mst.cpp:

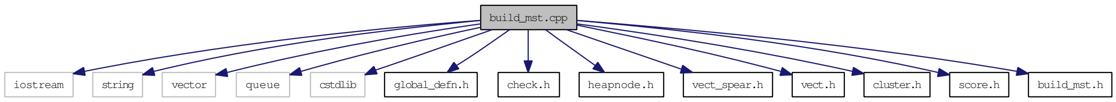

4.2 build_mst.h File Reference

This graph shows which files directly or indirectly include this file:

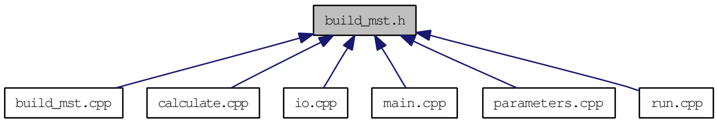

Classes

- class BUILDMST

4.3 calculate.cpp File Reference

```
#include <iostream>
#include <iomanip>
#include <string>
#include <vector>
#include <queue>
#include <fstream>
#include <cstdlib>
#include "global_defn.h"
#include "heapnode.h"
#include "vect_spear.h"
#include "vect.h"
#include "cluster.h"
#include "score.h"
#include "build_mst.h"
```

Include dependency graph for calculate.cpp:

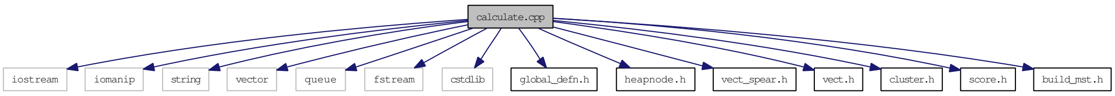

4.4 check.cpp File Reference

```
#include <string>
#include <cctype>
#include "check.h"
```

Include dependency graph for check.cpp:

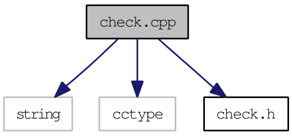

Functions

- string sanitizeFilename (string arg)

  Sanitize a filename by disallowing [/\].

- string sanitizePath (string arg)

  Sanitize a path by allowing alphanumeric characters and [./].

- string sanitizeURL (string arg)

  Sanitize a URL by allowing alphanumeric characters and [/:.].

4.4.1 Function Documentation

4.4.1.1 string sanitizeFilename (string *arg*)

Sanitize a filename by disallowing [/\].

We allow all characters since the filename is not as dangerous. However, we do not allow the slash or backslash characters to prevent a change in path and any form of escaping.

4.4.1.2 string sanitizePath (string *arg*)

Sanitize a path by allowing alphanumeric characters and [./].

Note that the backslash character has been purposely excluded to prevent any escaping. This will cause problems to the Windows' family of operating systems and should be added in, if required.

4.4.1.3 string sanitizeURL (string *arg*)

Sanitize a URL by allowing alphanumeric characters and [/:.].

Note that the backslash character has been purposely excluded to prevent any escaping.

4.5 check.h File Reference

This graph shows which files directly or indirectly include this file:

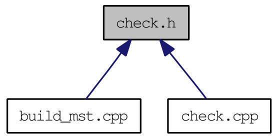

Functions

- string sanitizeFilename (string arg)

  Sanitize a filename by disallowing [/\].

- string sanitizePath (string arg)

  Sanitize a path by allowing alphanumeric characters and [./].

- string sanitizeURL (string arg)

  Sanitize a URL by allowing alphanumeric characters and [/:.].

4.5.1 Function Documentation

4.5.1.1 string sanitizeFilename (string *arg*)

Sanitize a filename by disallowing [/\].

We allow all characters since the filename is not as dangerous. However, we do not allow the slash or backslash characters to prevent a change in path and any form of escaping.

4.5.1.2 string sanitizePath (string *arg*)

Sanitize a path by allowing alphanumeric characters and [./].

Note that the backslash character has been purposely excluded to prevent any escaping. This will cause problems to the Windows' family of operating systems and should be added in, if required.

4.5.1.3 string sanitizeURL (string *arg*)

Sanitize a URL by allowing alphanumeric characters and [/:.].

Note that the backslash character has been purposely excluded to prevent any escaping.

4.6 cluster.cpp File Reference

```
#include <iostream>
#include <string>
#include <vector>
#include <boost/lexical_cast.hpp>
#include <cstdlib>
#include "global_defn.h"
#include "vect_spear.h"
#include "vect.h"
#include "cluster.h"
```

Include dependency graph for cluster.cpp:

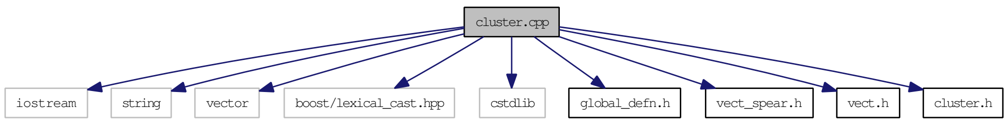

4.7 cluster.h File Reference

This graph shows which files directly or indirectly include this file:

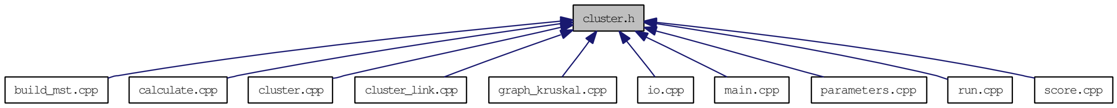

Classes

- class CLUSTER

4.8 cluster_link.cpp File Reference

```
#include <string>
#include <vector>
#include <cfloat>
#include "global_defn.h"
#include "vect_spear.h"
#include "vect.h"
#include "cluster.h"
```

Include dependency graph for cluster_link.cpp:

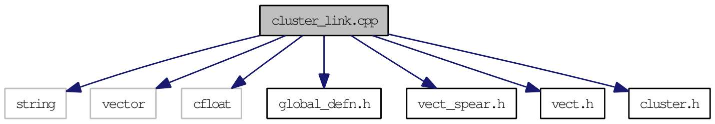

4.9 global_defn.h File Reference

This graph shows which files directly or indirectly include this file:

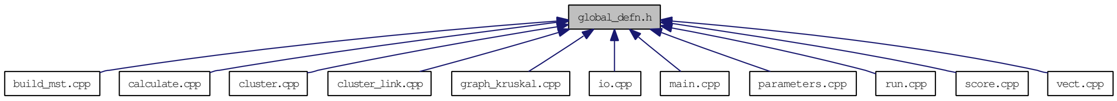

Defines

- #define VERBOSE_WIDTH 35

  Spacing for aligning the verbose output (in characters).

- #define CFG_FILENAME "build-mst.cfg"

  The default filename for the configuration file.

- #define SCORES_FILENAME "summary.txt"

  The default filename for the list of scores.

- #define EDGES_FILE_EXTENSION ".edges"

  File extension for the file of edges.

- #define NODES_FILE_EXTENSION ".nodes"

  File extension for the file of nodes.

- #define DEFAULT_COLOUR "gray"

  The default node colour.

- #define DEFAULT_SHAPE "ellipse"

  The default node shape.

- #define NULL_EXPR 0

  The numerical place-holder for a NULL expression; value does not matter.

Enumerations

- enum DIST_METHOD { DIST_EUC, DIST_MAN, DIST_PEAR, DIST_SPEAR }

  The distance method used.

- enum LINK_METHOD { LINK_SINGLE, LINK_AVERAGE, LINK_COMPLETE, LINK_-CENTROID }

  The linkage method used.

- enum SCORE_METHOD { SCORE_GAPS, SCORE_ANOVA, SCORE_NASSOC, SCORE_-NASSOC_ORIG }

  The scoring method used.

4.9.1 Define Documentation

4.9.1.1 #define CFG_FILENAME "build-mst.cfg"

The default filename for the configuration file.

4.9.1.2 #define DEFAULT_COLOUR "gray"

The default node colour.

4.9.1.3 #define DEFAULT_SHAPE "ellipse"

The default node shape.

4.9.1.4 #define EDGES_FILE_EXTENSION ".edges"

File extension for the file of edges.

4.9.1.5 #define NODES_FILE_EXTENSION ".nodes"

File extension for the file of nodes.

4.9.1.6 #define NULL_EXPR 0

The numerical place-holder for a NULL expression; value does not matter.

4.9.1.7 #define SCORES_FILENAME "summary.txt"

The default filename for the list of scores.

4.9.1.8 #define VERBOSE_WIDTH 35

Spacing for aligning the verbose output (in characters).

4.9.2 Enumeration Type Documentation

4.9.2.1 enum DIST_METHOD

The distance method used.

Enumerator:

DIST_EUC Euclidean distance

DIST_MAN Manhattan distance

DIST_PEAR Pearson correlation coefficient

DIST_SPEAR Spearman rank correlation coefficient

4.9.2.2 enum LINK_METHOD

The linkage method used.

Enumerator:

LINK_SINGLE Single linkage

LINK_AVERAGE Average linkage

LINK_COMPLETE Complete linkage

LINK_CENTROID Centroid linkage

4.9.2.3 enum SCORE_METHOD

The scoring method used.

Enumerator:

SCORE_GAPS Score based on gap size

SCORE_ANOVA Score based on ANOVA

SCORE_NASSOC Score based on the normalized association of Shi and Malik (2000) (modified)

SCORE_NASSOC_ORIG Score based on the normalized association of Shi and Malik (2000) (original)

4.10 graph.h File Reference

This graph shows which files directly or indirectly include this file:

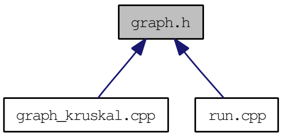

Classes

- class GRAPH

    Edge weight for undirected graphs.

Typedefs

- typedef boost::adjacency_list< boost::vecS, boost::vecS, boost::undirectedS, boost::no_property, boost::property< boost::edge_weight_t, double > > adjGraph

    An undirected graph represented as an adjacency list.

- typedef boost::graph_traits< adjGraph >::vertex_descriptor Vertex

    A vertex in the adjacency list graph.

- typedef boost::graph_traits< adjGraph >::edge_descriptor Edge

    An edge in the adjacency list graph.

- typedef pair< int, int > EdgePair

    A pair of edges represented as a pair of integers.

4.10.1 Typedef Documentation

4.10.1.1 typedef boost::adjacency_list< boost::vecS, boost::vecS, boost::undirectedS, boost::no_property, boost::property < boost::edge_weight_t, double > > adjGraph

An undirected graph represented as an adjacency list.

4.10.1.2 typedef boost::graph_traits< adjGraph >::edge_descriptor Edge

An edge in the adjacency list graph.

4.10.1.3 typedef pair<int, int> EdgePair

A pair of edges represented as a pair of integers.

4.10.1.4 typedef boost::graph_traits< adjGraph >::vertex_descriptor Vertex

A vertex in the adjacency list graph.

4.11 graph_kruskal.cpp File Reference

```
#include <fstream>
#include <vector>
#include <iostream>
#include <queue>
#include <boost/graph/adjacency_list.hpp>
#include <boost/graph/kruskal_min_spanning_tree.hpp>
#include <boost/lexical_cast.hpp>
#include "global_defn.h"
#include "vect_spear.h"
#include "vect.h"
#include "cluster.h"
#include "heapnode.h"
#include "graph.h"
```

Include dependency graph for graph_kruskal.cpp:

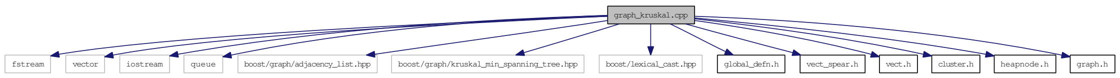

4.12 heapnode.cpp File Reference

```
#include <climits>
#include <cfloat>
#include "heapnode.h"
```

Include dependency graph for heapnode.cpp:

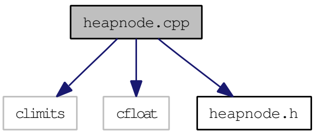

4.13 heapnode.h File Reference

This graph shows which files directly or indirectly include this file:

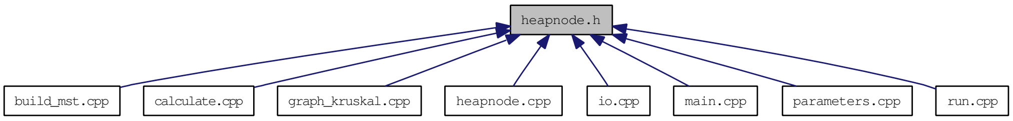

Classes

- class HEAPNODE

4.14 io.cpp File Reference

```
#include <iostream>
#include <iomanip>
#include <fstream>
#include <queue>
#include <boost/tokenizer.hpp>
#include "global_defn.h"
#include "heapnode.h"
#include "vect_spear.h"
#include "vect.h"
#include "cluster.h"
#include "score.h"
#include "build_mst.h"
```

Include dependency graph for io.cpp:

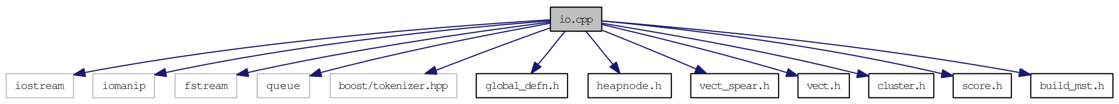

4.15 main.cpp File Reference

```
#include <iostream>
#include <string>
#include <vector>
#include <queue>
#include <cstdlib>
#include "global_defn.h"
#include "heapnode.h"
#include "vect_spear.h"
#include "vect.h"
#include "cluster.h"
#include "score.h"
#include "build_mst.h"
```

Include dependency graph for main.cpp:

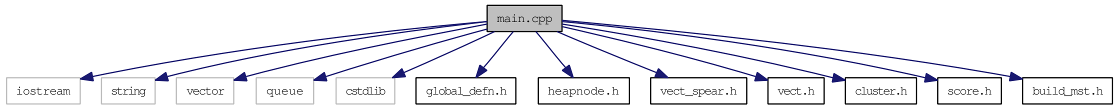

Functions

- int main (int argc, char ∗argv[ ])

  The main () function of the program.

4.15.1 Function Documentation

4.15.1.1 int main (int *argc*, char ∗ *argv*[ ])

The main () function of the program.

Create a BUILDMST object and then uses it to read in the parameters from the file and the command line. If all the settings check out, then run the main program.

Here is the call graph for this function:

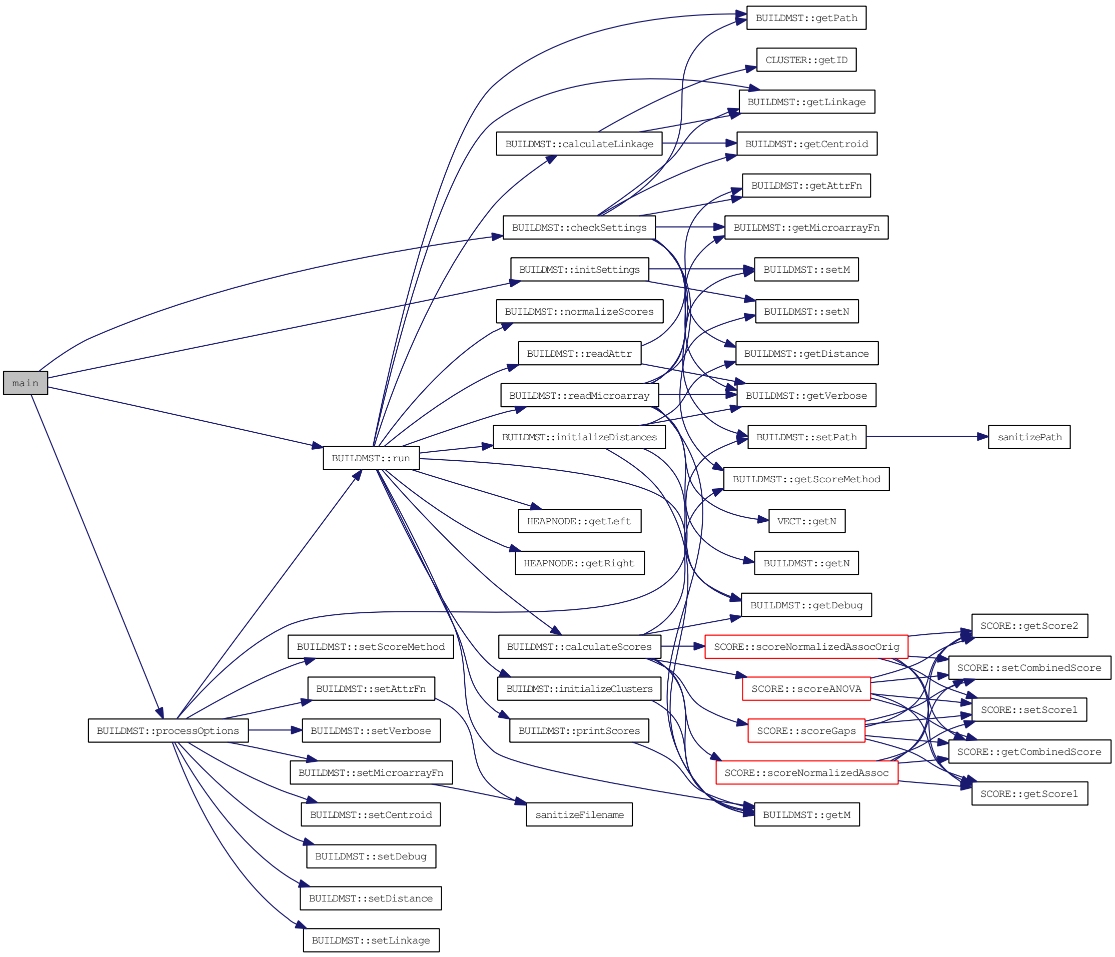

4.16 parameters.cpp File Reference

```
#include <iostream>
#include <iomanip>
#include <fstream>
#include <string>
#include <vector>
#include <queue>
#include "config.h"
#include <boost/program_options.hpp>
#include "global_defn.h"
#include "heapnode.h"
#include "vect_spear.h"
#include "vect.h"
#include "cluster.h"
#include "score.h"
#include "build_mst.h"
```

Include dependency graph for parameters.cpp:

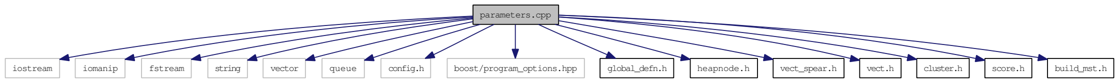

4.17 run.cpp File Reference

```
#include <iostream>
#include <string>
#include <vector>
#include <queue>
#include <boost/graph/adjacency_list.hpp>
#include <boost/graph/adjacency_matrix.hpp>
#include "global_defn.h"
#include "heapnode.h"
#include "vect_spear.h"
#include "vect.h"
#include "cluster.h"
#include "graph.h"
#include "score.h"
#include "build_mst.h"
```

Include dependency graph for run.cpp:

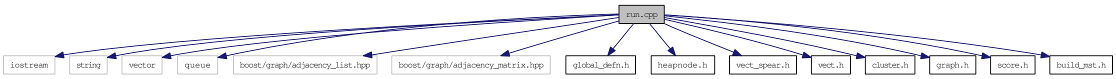

4.18 score.cpp File Reference

#include <iostream>

#include <string>

#include <vector>

#include <cmath>

#include <climits>

#include <cfloat>

#include "global_defn.h"

#include "vect_spear.h"

#include "vect.h"

#include "cluster.h"

#include "score.h"

Include dependency graph for score.cpp:

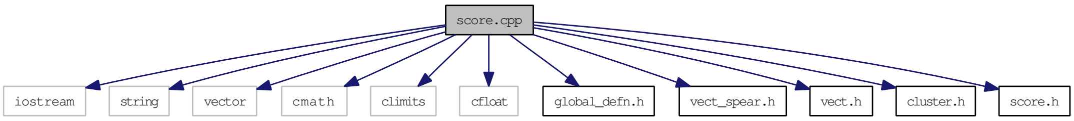

4.19 score.h File Reference

This graph shows which files directly or indirectly include this file:

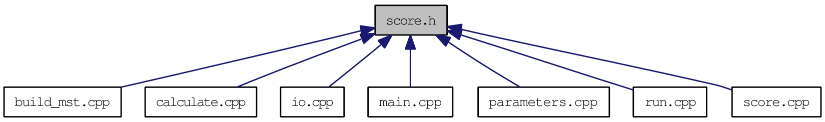

Classes

- class SCORE

4.20 vect.cpp File Reference

```
#include <string>
#include <vector>
#include <iostream>
#include <boost/tokenizer.hpp>
#include <boost/lexical_cast.hpp>
#include "global_defn.h"
#include "vect_spear.h"
#include "vect.h"
```

Include dependency graph for vect.cpp:

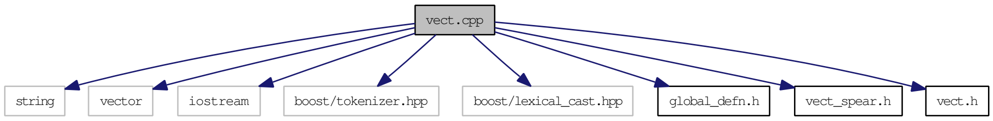

4.21 vect.h File Reference

This graph shows which files directly or indirectly include this file:

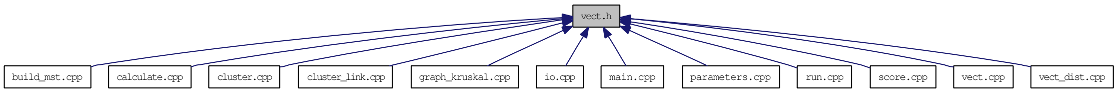

Classes

- class VECT

4.22 vect_dist.cpp File Reference

```
#include <iostream>
#include <string>
#include <vector>
#include <algorithm>
#include <cmath>
#include <cfloat>
#include <cstdlib>
#include "vect_spear.h"
#include "vect.h"
```

Include dependency graph for vect_dist.cpp:

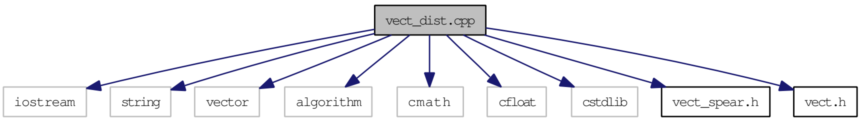

4.23 vect_spear.cpp File Reference

```
#include <vector>
#include <iostream>
#include "vect_spear.h"
```

Include dependency graph for vect_spear.cpp:

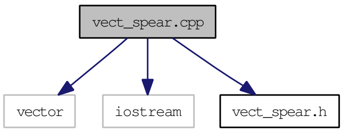

4.24 vect_spear.h File Reference

This graph shows which files directly or indirectly include this file:

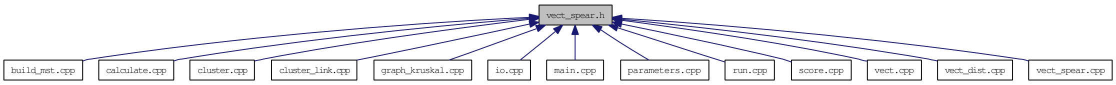

Classes

- class SPEARMAN

Index

adjGraph
graph.h, 60
ancestors
CLUSTER, 25
attr_fn
BUILDMST, 17

build_mst.cpp, 49
build_mst.h, 50
BUILDMST, 5
attr_fn, 17
BUILDMST, 8
calculateLinkage, 13
calculateScores, 13
centroid, 17
checkSettings, 9
clusters, 17
data, 17
debug_flag, 16
dist_matrix, 17
distance, 16
getAttrFn, 15
getCentroid, 15
getDebug, 14
getDistance, 14
getLinkage, 14
getM, 16
getMicroarrayFn, 15
getN, 16
getPath, 16
getScoreMethod, 15
getVerbose, 14
initializeClusters, 12
initializeDistances, 12
initSettings, 9
linkage, 16
M, 17
microarray_fn, 17
N, 17
normalizeScores, 13
path, 17
pqueue, 17
printScores, 14
processOptions, 8
readAttr, 12
readMicroarray, 11
run, 10
scores, 17
scoring, 16
setAttrFn, 15
setCentroid, 15
setDebug, 14
setDistance, 14
setLinkage, 14
setM, 16
setMicroarrayFn, 15
setN, 16
setPath, 15
setScoreMethod, 15
setVerbose, 14
verbose_flag, 16

calculate.cpp, 51
calculateLinkage
BUILDMST, 13
calculateScores
BUILDMST, 13
centroid
BUILDMST, 17
CLUSTER, 25
CFG_FILENAME
global_defn.h, 58
check.cpp, 52
sanitizeFilename, 52
sanitizePath, 52
sanitizeURL, 52
check.h, 53
sanitizeFilename, 53
sanitizePath, 53
sanitizeURL, 53
checkSettings
BUILDMST, 9
CLUSTER, 19
ancestors, 25
centroid, 25
CLUSTER, 21
colour, 24
formCentroid, 23
getCentroid, 23
getColour, 22

getID, 22
getItems, 23
getName, 22
getShape, 22
haveAncestors, 23
id, 24
items, 25
linkAverage, 23
linkCentroid, 24
linkComplete, 24
linkSingle, 23
name, 24
setAncestors, 23
setColour, 22
setName, 22
setShape, 22
shape, 25
cluster.cpp, 54
cluster.h, 55
cluster_link.cpp, 56
clusters
BUILDMST, 17
colour
CLUSTER, 24
VECT, 47
combined
SCORE, 37
copyOrigPosToKey
SPEARMAN, 40

data
BUILDMST, 17
debug_flag
BUILDMST, 16
DEFAULT_COLOUR
global_defn.h, 58
DEFAULT_SHAPE
global_defn.h, 58
DIST_EUC
global_defn.h, 58
DIST_MAN
global_defn.h, 58
DIST_PEAR
global_defn.h, 58
DIST_SPEAR
global_defn.h, 58
dist_matrix
BUILDMST, 17
DIST_METHOD
global_defn.h, 58
distance
BUILDMST, 16

Edge
graph.h, 60
EdgePair
graph.h, 60
edges
GRAPH, 27
EDGES_FILE_EXTENSION
global_defn.h, 58
exprs
VECT, 47

formCentroid
CLUSTER, 23

g
GRAPH, 27
getAttrFn
BUILDMST, 15
getCentroid
BUILDMST, 15
CLUSTER, 23
getColour
CLUSTER, 22
VECT, 44
getCombinedScore
SCORE, 35
getDebug
BUILDMST, 14
getDistance
BUILDMST, 14
getExpr
VECT, 44
getID
CLUSTER, 22
SCORE, 34
VECT, 44
getItems
CLUSTER, 23
getLeft
HEAPNODE, 30
SCORE, 34
getLinkage
BUILDMST, 14
getM
BUILDMST, 16
getMicroarrayFn
BUILDMST, 15
getN
BUILDMST, 16
VECT, 45
getName
CLUSTER, 22
VECT, 44
getNull
VECT, 45

getOrigPos
SPEARMAN, 39
getPath
BUILDMST, 16
getRank
SPEARMAN, 39
getRight
HEAPNODE, 30
SCORE, 34
getScore
HEAPNODE, 30
getScore1
SCORE, 34
getScore2
SCORE, 35
getScoreMethod
BUILDMST, 15
getShape
CLUSTER, 22
VECT, 44
getValue
SPEARMAN, 39
getVerbose
BUILDMST, 14
global_defn.h
DIST_EUC, 58
DIST_MAN, 58
DIST_PEAR, 58
DIST_SPEAR, 58
LINK_AVERAGE, 59
LINK_CENTROID, 59
LINK_COMPLETE, 59
LINK_SINGLE, 59
SCORE_ANOVA, 59
SCORE_GAPS, 59
SCORE_NASSOC, 59
SCORE_NASSOC_ORIG, 59
global_defn.h, 57
CFG_FILENAME, 58
DEFAULT_COLOUR, 58
DEFAULT_SHAPE, 58
DIST_METHOD, 58
EDGES_FILE_EXTENSION, 58
LINK_METHOD, 58
NODES_FILE_EXTENSION, 58
NULL_EXPR, 58
SCORE_METHOD, 59
SCORES_FILENAME, 58
VERBOSE_WIDTH, 58
GRAPH, 26
edges, 27
g, 27
GRAPH, 27
printEdges, 27
printNodes, 27
spanning_tree_edges, 28
spanning_tree_vertices, 28
weights, 27
weights_pmap, 27
graph.h, 60
adjGraph, 60
Edge, 60
EdgePair, 60
Vertex, 60
graph_kruskal.cpp, 62

haveAncestors
CLUSTER, 23
HEAPNODE, 29
getLeft, 30
getRight, 30
getScore, 30
HEAPNODE, 30
left, 30
operator<, 30
operator>, 30
right, 30
score, 30
heapnode.cpp, 63
heapnode.h, 64

id
CLUSTER, 24
SCORE, 37
VECT, 47
initializeClusters
BUILDMST, 12
initializeDistances
BUILDMST, 12
initSettings
BUILDMST, 9
io.cpp, 65
isNull
VECT, 45
items
CLUSTER, 25

key
SPEARMAN, 40

left
HEAPNODE, 30
SCORE, 37
LINK_AVERAGE
global_defn.h, 59
LINK_CENTROID
global_defn.h, 59
LINK_COMPLETE

global_defn.h, 59
LINK_SINGLE
global_defn.h, 59
LINK_METHOD
global_defn.h, 58
linkage
BUILDMST, 16
linkAverage
CLUSTER, 23
linkCentroid
CLUSTER, 24
linkComplete
CLUSTER, 24
linkSingle
CLUSTER, 23

M
BUILDMST, 17
main
main.cpp, 66
main.cpp, 66
main, 66
microarray_fn
BUILDMST, 17

N
BUILDMST, 17
name
CLUSTER, 24
VECT, 47
NODES_FILE_EXTENSION
global_defn.h, 58
normalizeScores
BUILDMST, 13
NULL_EXPR
global_defn.h, 58
nulls
VECT, 47

operator<
HEAPNODE, 30
SPEARMAN, 39
operator>
HEAPNODE, 30
origpos
SPEARMAN, 40

parameters.cpp, 68
path
BUILDMST, 17
pqueue
BUILDMST, 17
printEdges
GRAPH, 27
printNodes
GRAPH, 27
printScores
BUILDMST, 14
processOptions
BUILDMST, 8
putExpr
VECT, 45
putNull
VECT, 45

rank
SPEARMAN, 40
readAttr
BUILDMST, 12
readMicroarray
BUILDMST, 11
resize
VECT, 45
right
HEAPNODE, 30
SCORE, 37
run
BUILDMST, 10
run.cpp, 69

sanitizeFilename
check.cpp, 52
check.h, 53
sanitizePath
check.cpp, 52
check.h, 53
sanitizeURL
check.cpp, 52
check.h, 53
SCORE, 32
combined, 37
getCombinedScore, 35
getID, 34
getLeft, 34
getRight, 34
getScore1, 34
getScore2, 35
id, 37
left, 37
right, 37
SCORE, 33
score1, 37
score2, 37
scoreANOVA, 35
scoreGaps, 35
scoreNormalizedAssoc, 36
scoreNormalizedAssocOrig, 36
setCombinedScore, 34

setID, 34
setLeft, 34
setRight, 34
setScore1, 34
setScore2, 34
score
HEAPNODE, 30
score.cpp, 70
score.h, 71
score1
SCORE, 37
score2
SCORE, 37
SCORE_ANOVA
global_defn.h, 59
SCORE_GAPS
global_defn.h, 59
SCORE_NASSOC
global_defn.h, 59
SCORE_NASSOC_ORIG
global_defn.h, 59
SCORE_METHOD
global_defn.h, 59
scoreANOVA
SCORE, 35
scoreGaps
SCORE, 35
scoreNormalizedAssoc
SCORE, 36
scoreNormalizedAssocOrig
SCORE, 36
scores
BUILDMST, 17
SCORES_FILENAME
global_defn.h, 58
scoring
BUILDMST, 16
setAncestors
CLUSTER, 23
setAttrFn
BUILDMST, 15
setCentroid
BUILDMST, 15
setColour
CLUSTER, 22
VECT, 44
setCombinedScore
SCORE, 34
setDebug
BUILDMST, 14
setDistance
BUILDMST, 14
setID
SCORE, 34
VECT, 44
setLeft
SCORE, 34
setLinkage
BUILDMST, 14
setM
BUILDMST, 16
setMicroarrayFn
BUILDMST, 15
setN
BUILDMST, 16
setName
CLUSTER, 22
VECT, 44
setOrigPos
SPEARMAN, 39
setPath
BUILDMST, 15
setRank
SPEARMAN, 39
setRight
SCORE, 34
setScore1
SCORE, 34
setScore2
SCORE, 34
setScoreMethod
BUILDMST, 15
setShape
CLUSTER, 22
VECT, 44
setValue
SPEARMAN, 39
setVerbose
BUILDMST, 14
shape
CLUSTER, 25
VECT, 47
simEuc
VECT, 45
simMan
VECT, 45
simPear
VECT, 46
simSpear
VECT, 46
spanning_tree_edges
GRAPH, 28
spanning_tree_vertices
GRAPH, 28
SPEARMAN, 38
copyOrigPosToKey, 40
getOrigPos, 39
getRank, 39

getValue, 39
key, 40
operator<, 39
origpos, 40
rank, 40
setOrigPos, 39
setRank, 39
setValue, 39
SPEARMAN, 39
value, 40

value
SPEARMAN, 40
VECT, 41
colour, 47
exprs, 47
getColour, 44
getExpr, 44
getID, 44
getN, 45
getName, 44
getNull, 45
getShape, 44
id, 47
isNull, 45
name, 47
nulls, 47
putExpr, 45
putNull, 45
resize, 45
setColour, 44
setID, 44
setName, 44
setShape, 44
shape, 47
simEuc, 45
simMan, 45
simPear, 46
simSpear, 46
VECT, 43
vect.cpp, 72
vect.h, 73
vect_dist.cpp, 74
vect_spear.cpp, 75
vect_spear.h, 76
verbose_flag
BUILDMST, 16
VERBOSE_WIDTH
global_defn.h, 58
Vertex
graph.h, 60

weights
GRAPH, 27
weights_pmap
GRAPH, 27
