# Supplementary material for: HAMSTER: visualizing microarray experiments as a set of minimum spanning trees
Source: Source Code Biol Med. 2009 Nov 20;4:8. doi: 10.1186/1751-0473-4-8 (PMC2784758; doi:10.1186/1751-0473-4-8)
Supplement: Additional file 2 — HAMSTER - layout-mst. Source code documentation for layout-mst generated using Doxygen [24]. [file 1751-0473-4-8-S2.PDF]

Hamster - layout-mst

1.2

Generated by Doxygen 1.5.6

Sun Oct 11 18:19:58 2009

Contents

1 Class Index **1**

1.1 Class List 1

2 File Index **3**

2.1 File List 3

3 Class Documentation **5**

3.1 EDGE Class Reference 5

3.1.1 Detailed Description 6

3.1.2 Constructor & Destructor Documentation 6

3.1.2.1 EDGE 6

3.1.2.2 EDGE 6

3.1.3 Member Function Documentation 6

3.1.3.1 setStart 6

3.1.3.2 getStart 6

3.1.3.3 setEnd 6

3.1.3.4 getEnd 7

3.1.3.5 setWeight 7

3.1.3.6 getWeight 7

3.1.4 Member Data Documentation 7

3.1.4.1 start 7

3.1.4.2 end 7

3.1.4.3 weight 7

3.2 LAYOUTMST Class Reference 8

3.2.1 Detailed Description 13

3.2.2 Constructor & Destructor Documentation 13

3.2.2.1 LAYOUTMST 13

3.2.3 Member Function Documentation 13

3.2.3.1 processOptions 13

3.2.3.2 initSettings . . . 14
3.2.3.3 checkSettings . . . 14
3.2.3.4 sendOKFail . . . 15
3.2.3.5 recvOKFail . . . 16
3.2.3.6 runPrimary . . . 16
3.2.3.7 runAllProcessors . . . 18
3.2.3.8 readScores . . . 20
3.2.3.9 processScores . . . 20
3.2.3.10 expandPacket . . . 20
3.2.3.11 broadcastData . . . 22
3.2.3.12 receiveData . . . 24
3.2.3.13 callGraphViz . . . 26
3.2.3.14 process . . . 26
3.2.3.15 printGraphViz . . . 27
3.2.3.16 readNodes . . . 28
3.2.3.17 readEdges . . . 28
3.2.3.18 updateNodePositions . . . 28
3.2.3.19 setDebug . . . 29
3.2.3.20 getDebug . . . 29
3.2.3.21 setVerbose . . . 29
3.2.3.22 getVerbose . . . 29
3.2.3.23 setPreview . . . 29
3.2.3.24 getPreview . . . 29
3.2.3.25 setFixedPos . . . 29
3.2.3.26 getFixedPos . . . 29
3.2.3.27 setPath . . . 29
3.2.3.28 getPath . . . 29
3.2.3.29 setURL . . . 30
3.2.3.30 getURL . . . 30
3.2.3.31 setWidth . . . 30
3.2.3.32 getWidth . . . 30
3.2.3.33 setHeight . . . 30
3.2.3.34 getHeight . . . 30
3.2.3.35 setPWidth . . . 30
3.2.3.36 getPWidth . . . 30
3.2.3.37 setPHeight . . . 30

3.2.3.38 getPHeight 30
3.2.3.39 setOuttype 31
3.2.3.40 getOuttype 31
3.2.3.41 setPercent 31
3.2.3.42 getPercent 31
3.2.3.43 setDPI 31
3.2.3.44 getDPI 31
3.2.3.45 setSpline 31
3.2.3.46 getSpline 31
3.2.3.47 setFontsize 31
3.2.3.48 getFontsize 31
3.2.3.49 setTotalIter 31
3.2.3.50 getTotalIter 32
3.2.3.51 setScoresFn 32
3.2.3.52 getScoresFn 32
3.2.3.53 setMyWorkunit 32
3.2.3.54 getMyWorkunit 32
3.2.3.55 setAllWorkunits 32
3.2.3.56 getAllWorkunits 32
3.2.3.57 setRank 32
3.2.3.58 getRank 33
3.2.3.59 setWorldSize 33
3.2.3.60 getWorldSize 33
3.2.4 Member Data Documentation 33
3.2.4.1 debug_flag 33
3.2.4.2 verbose_flag 33
3.2.4.3 preview_flag 33
3.2.4.4 fixed_pos 33
3.2.4.5 path 33
3.2.4.6 url 33
3.2.4.7 width 33
3.2.4.8 height 33
3.2.4.9 pwidth 34
3.2.4.10 pheight 34
3.2.4.11 outtype 34
3.2.4.12 percent 34

3.2.4.13 dpi . . . 34
3.2.4.14 spline_flag . . . 34
3.2.4.15 fontsize . . . 34
3.2.4.16 vertices . . . 34
3.2.4.17 edges . . . 34
3.2.4.18 total_iter . . . 34
3.2.4.19 scores_fn . . . 34
3.2.4.20 scores . . . 35
3.2.4.21 all_workunits . . . 35
3.2.4.22 my_workunit . . . 35
3.2.4.23 rank . . . 35
3.2.4.24 world_size . . . 35
3.3 PACKET Class Reference . . . 36
3.3.1 Detailed Description . . . 39
3.3.2 Constructor & Destructor Documentation . . . 39
3.3.2.1 PACKET . . . 39
3.3.3 Member Function Documentation . . . 39
3.3.3.1 setDebug . . . 39
3.3.3.2 getDebug . . . 39
3.3.3.3 setVerbose . . . 39
3.3.3.4 getVerbose . . . 39
3.3.3.5 setPreview . . . 39
3.3.3.6 getPreview . . . 39
3.3.3.7 setFixedPos . . . 40
3.3.3.8 getFixedPos . . . 40
3.3.3.9 setPath . . . 40
3.3.3.10 getPath . . . 40
3.3.3.11 setURL . . . 40
3.3.3.12 getURL . . . 40
3.3.3.13 setWidth . . . 40
3.3.3.14 getWidth . . . 40
3.3.3.15 setHeight . . . 40
3.3.3.16 getHeight . . . 40
3.3.3.17 setPWidth . . . 40
3.3.3.18 getPWidth . . . 41
3.3.3.19 setPHeight . . . 41

3.3.3.20 getPHeight 41
3.3.3.21 setOuttype 41
3.3.3.22 getOuttype 41
3.3.3.23 setPercent 41
3.3.3.24 getPercent 41
3.3.3.25 setDPI 41
3.3.3.26 getDPI 41
3.3.3.27 setSpline 41
3.3.3.28 getSpline 41
3.3.3.29 setFontsize 42
3.3.3.30 getFontsize 42
3.3.3.31 setMyWorkunit 42
3.3.3.32 getMyWorkunit 42
3.3.4 Member Data Documentation 42
3.3.4.1 debug_flag 42
3.3.4.2 verbose_flag 42
3.3.4.3 preview_flag 42
3.3.4.4 fixed_pos 42
3.3.4.5 path 42
3.3.4.6 url 42
3.3.4.7 width 42
3.3.4.8 height 43
3.3.4.9 pwidth 43
3.3.4.10 pheight 43
3.3.4.11 outtype 43
3.3.4.12 percent 43
3.3.4.13 dpi 43
3.3.4.14 spline_flag 43
3.3.4.15 fontsize 43
3.3.4.16 my_workunit 43
3.4 SCORE Class Reference 44
3.4.1 Detailed Description 45
3.4.2 Constructor & Destructor Documentation 45
3.4.2.1 SCORE 45
3.4.2.2 SCORE 45
3.4.3 Member Function Documentation 46

3.4.3.1 setID 46
3.4.3.2 setLeft 46
3.4.3.3 setRight 46
3.4.3.4 setScore1 46
3.4.3.5 setScore2 46
3.4.3.6 setCombinedScore 46
3.4.3.7 getID 46
3.4.3.8 getLeft 46
3.4.3.9 getRight 46
3.4.3.10 getScore1 46
3.4.3.11 getScore2 47
3.4.3.12 getCombinedScore 47
3.4.3.13 operator< 47
3.4.3.14 operator> 47
3.4.4 Member Data Documentation 47
3.4.4.1 id 47
3.4.4.2 left 47
3.4.4.3 right 47
3.4.4.4 score1 47
3.4.4.5 score2 47
3.4.4.6 combined 47
3.5 VERTEX Class Reference 48
3.5.1 Detailed Description 50
3.5.2 Constructor & Destructor Documentation 50
3.5.2.1 VERTEX 50
3.5.2.2 VERTEX 50
3.5.3 Member Function Documentation 50
3.5.3.1 setName 50
3.5.3.2 getName 50
3.5.3.3 setColour 50
3.5.3.4 getColour 50
3.5.3.5 setShape 50
3.5.3.6 getShape 50
3.5.3.7 setComponents 51
3.5.3.8 getComponents 51
3.5.3.9 setUpdated 51

3.5.3.10 getUpdated 51
3.5.3.11 setX 51
3.5.3.12 getX 51
3.5.3.13 setY 51
3.5.3.14 getY 51
3.5.3.15 setWidth 51
3.5.3.16 getWidth 51
3.5.3.17 setHeight 51
3.5.3.18 getHeight 52
3.5.4 Member Data Documentation 52
3.5.4.1 name 52
3.5.4.2 colour 52
3.5.4.3 shape 52
3.5.4.4 updated 52
3.5.4.5 components 52
3.5.4.6 x 52
3.5.4.7 y 52
3.5.4.8 width 52
3.5.4.9 height 52

4 File Documentation 53

4.1 check.cpp File Reference 53
4.1.1 Function Documentation 53
4.1.1.1 sanitizeFilename 53
4.1.1.2 sanitizePath 54
4.1.1.3 sanitizeURL 54
4.2 check.h File Reference 55
4.2.1 Function Documentation 55
4.2.1.1 sanitizeFilename 55
4.2.1.2 sanitizePath 55
4.2.1.3 sanitizeURL 55
4.3 edge.cpp File Reference 56
4.4 edge.h File Reference 57
4.5 global_defn.h File Reference 58
4.5.1 Define Documentation 60
4.5.1.1 CFG_FILENAME 60
4.5.1.2 DEFAULT_DPI 60

4.5.1.3 DEFAULT_FONTSIZE . . . 60
4.5.1.4 DEFAULT_HEIGHT . . . 60
4.5.1.5 DEFAULT_PERCENT . . . 60
4.5.1.6 DEFAULT_PHEIGHT . . . 60
4.5.1.7 DEFAULT_PWIDTH . . . 60
4.5.1.8 DEFAULT_URL . . . 60
4.5.1.9 DEFAULT_WIDTH . . . 60
4.5.1.10 DOT_FILE_EXTENSION . . . 60
4.5.1.11 EDGES_FILE_EXTENSION . . . 60
4.5.1.12 ENABLE_LARGE_NODE . . . 61
4.5.1.13 GV_FILE_EXTENSION . . . 61
4.5.1.14 GVPV_FILE_EXTENSION . . . 61
4.5.1.15 LARGE_NODE_INCREASE . . . 61
4.5.1.16 LARGE_NODE_THRESH . . . 61
4.5.1.17 MAX_DIM . . . 61
4.5.1.18 MAX_DPI . . . 61
4.5.1.19 MAX_FONTSIZE . . . 61
4.5.1.20 MIN_DIM . . . 61
4.5.1.21 MIN_DPI . . . 61
4.5.1.22 MIN_FONTSIZE . . . 61
4.5.1.23 NODES_FILE_EXTENSION . . . 62
4.5.1.24 SCALE_FACTOR . . . 62
4.5.1.25 SCORES_FIELDS . . . 62
4.5.1.26 VERBOSE_WIDTH . . . 62
4.5.2 Enumeration Type Documentation . . . 62
4.5.2.1 FILETYPE . . . 62
4.6 graphviz.cpp File Reference . . . 63
4.7 io.cpp File Reference . . . 64
4.8 layout_mst.cpp File Reference . . . 65
4.9 layout_mst.h File Reference . . . 66
4.10 main.cpp File Reference . . . 67
4.10.1 Function Documentation . . . 67
4.10.1.1 main . . . 67
4.11 packet.cpp File Reference . . . 68
4.12 packet.h File Reference . . . 69
4.13 parameters.cpp File Reference . . . 70

4.14 process_scores.cpp File Reference 71
4.14.1 Function Documentation 71
4.14.1.1 scoresGreaterCmp 71
4.14.1.2 scoresGreaterIDCmp 71
4.15 run.cpp File Reference 73
4.16 score.cpp File Reference 74
4.17 score.h File Reference 75
4.18 transmit.cpp File Reference 76
4.19 vertex.cpp File Reference 77
4.20 vertex.h File Reference 78

Chapter 1

Class Index

1.1 Class List

Here are the classes, structs, unions and interfaces with brief descriptions:

EDGE 5
LAYOUTMST 8
PACKET 36
SCORE 44
VERTEX 48

Chapter 2

File Index

2.1 File List

Here is a list of all files with brief descriptions:

check.cpp 53
check.h 55
edge.cpp 56
edge.h 57
global_defn.h 58
graphviz.cpp 63
io.cpp 64
layout_mst.cpp 65
layout_mst.h 66
main.cpp 67
packet.cpp 68
packet.h 69
parameters.cpp 70
process_scores.cpp 71
run.cpp 73
score.cpp 74
score.h 75
transmit.cpp 76
vertex.cpp 77
vertex.h 78

Chapter 3

Class Documentation

3.1 EDGE Class Reference

`#include <edge.h>`

Public Member Functions

- EDGE ()

  Default constructor that takes no arguments.

- EDGE (string arg1, string arg2, double arg3)

  Default constructor that takes three arguments.

- void setStart (string arg)

  Set the start vertex name.

- string getStart () const

  Get the start vertex name.

- void setEnd (string arg)

  Set the end vertex name.

- string getEnd () const

  Get the end vertex name.

- void setWeight (double arg)

  Set the edge weight.

- double getWeight () const

  Get the edge weight.

Private Attributes

- string start

    Edge start.

- string end

    Edge end.

- double weight

    Edge weight.

3.1.1 Detailed Description

An EDGE contains three attributes: start, end, and weight. As the graph is undirected, there assignment to start and end is arbitrary. The edge weight is a floating point value.

3.1.2 Constructor & Destructor Documentation

3.1.2.1 EDGE::EDGE ()

Default constructor that takes no arguments.

3.1.2.2 EDGE::EDGE (string *arg1*, string *arg2*, double *arg3*)

Default constructor that takes three arguments.

Parameters:

arg1 Edge start point

arg2 Edge end point

arg3 Edge weight

3.1.3 Member Function Documentation

3.1.3.1 void EDGE::setStart (string *arg*)

Set the start vertex name.

3.1.3.2 string EDGE::getStart () const

Get the start vertex name.

3.1.3.3 void EDGE::setEnd (string *arg*)

Set the end vertex name.

3.1.3.4 string EDGE::getEnd () const

Get the end vertex name.

3.1.3.5 void EDGE::setWeight (double *arg*)

Set the edge weight.

3.1.3.6 double EDGE::getWeight () const

Get the edge weight.

3.1.4 Member Data Documentation

3.1.4.1 string EDGE::start `[private]`

Edge start.

3.1.4.2 string EDGE::end `[private]`

Edge end.

3.1.4.3 double EDGE::weight `[private]`

Edge weight.

The documentation for this class was generated from the following files:

- edge.h
- edge.cpp

3.2 LAYOUTMST Class Reference

```
#include <layout_mst.h>
```

Public Member Functions

- LAYOUTMST ()

  Default constructor that takes no arguments.

- bool processOptions (int argc, char ∗argv[ ])

  Process options from the command line and the configuration file CFG_FILENAME.

- void initSettings ()

  Initialize settings when there is no MPI available.

- bool checkSettings ()

  Check the settings to ensure they are valid.

- void sendOKFail (bool arg)

  Send the initial signal to all processes to tell them to continue or not.

- bool recvOKFail ()

  Receive the initial signal from the primary process to determine whether to continue or not.

- bool runPrimary ()

  Execute the part of the program that is for the primary processor only.

- void runAllProcessors ()

  Execute the part of the program that is for all processors (including the primary).

- bool readScores ()

  Read the data file of scores in.

- void processScores ()

  Process the scores by sorting and inserting into separate lists.

- void expandPacket (PACKET ∗p)

  Expand the information in a PACKET object and set the private variables.

- void broadcastData ()

  Construct PACKET objects for each processor and send it to them.

- void receiveData ()

  Receive PACKET objects from the primary processor.

- void callGraphViz (unsigned int id, enum FILETYPE in_ftype, enum FILETYPE out_ftype)

  Call GraphViz by executing it as a shell command.

- void process (unsigned int id)

Process a single MST.

- void printGraphViz (unsigned int id, double width, double height, string extension, vector< VERTEX > vertices, vector< EDGE > edges)

  Output the graph information in GraphViz format.

- void readNodes (unsigned int id)

  Read in the nodes from file.

- double readEdges (unsigned int id)

  Read in the edges from file.

- void updateNodePositions (unsigned int id)

  Update the node positions.

- void setDebug (bool arg)
- bool getDebug () const

  Get the debug setting.

- void setVerbose (bool arg)

  Set whether or not verbose output is required.

- bool getVerbose () const

  Get the verbose setting.

- void setPreview (bool arg)

  Set whether or not preview images are required.

- bool getPreview () const

  Get the preview setting.

- void setFixedPos (bool arg)

  Set whether or not node positions are fixed using the previous MST.

- bool getFixedPos () const

  Get the fixed node position setting.

- void setPath (string arg)

  Set the input/output path (the path where files are).

- string getPath () const

  Get the output path.

- void setURL (string arg)

  Set the URL for image maps.

- string getURL () const

  Get the URL.

- void setWidth (double arg)

Set the width of the images.

- double getWidth () const

  Get the width of the images.

- void setHeight (double arg)

  Set the height of the images.

- double getHeight () const

  Get the height of the images.

- void setPWidth (double arg)

  Set the width of the preview images.

- double getPWidth () const

  Get the width of the preview images.

- void setPHeight (double arg)

  Set the height of the preview images.

- double getPHeight () const

  Get the height of the preview images.

- void setOuttype (enum FILETYPE)

  Set the type of output image.

- enum FILETYPE getOuttype () const

  Get the type of output image.

- void setPercent (unsigned int arg)

  Set the percent.

- unsigned int getPercent () const

  Get the percent.

- void setDPI (unsigned int arg)

  Set the DPI.

- unsigned int getDPI () const

  Get the DPI.

- void setSpline (bool arg)

  Set the spline flag.

- bool getSpline () const

  Get the spline flag.

- void setFontsize (unsigned int arg)

  Set the font size.

- unsigned int getFontsize () const

  Get the font size.

- void setTotalIter (unsigned int arg)
- unsigned int getTotalIter () const

  Get the total number of iterations.

- void setScoresFn (string arg)

  Set the scores filename.

- string getScoresFn () const

  Get the scores filename.

- void setMyWorkunit (vector< unsigned int > arg)
- vector< unsigned int > getMyWorkunit () const

  Get the workunits for the current process to do.

- void setAllWorkunits (unsigned int arg1, vector< unsigned int > arg2)

  Set the workunits for the child process arg1 to do (used by the primary processor only).

- vector< unsigned int > getAllWorkunits (unsigned int arg) const

  Get the workunits for the child process arg1 to do from the primary processor.

- void setRank (unsigned int arg)

  Set the rank for the current process.

- unsigned int getRank () const

  Get the rank for the current process.

- void setWorldSize (unsigned int arg)

  Get the total number of processes.

- unsigned int getWorldSize () const

  Set the total number of processes.

Private Attributes

- bool debug_flag

  Set to true if debug output is required; false otherwise.

- bool verbose_flag

  Set to true if verbose output is required; false otherwise.

- bool preview_flag

  Set to true if smaller, preview images are required; false otherwise.

- bool fixed_pos

  Set to true if each MSTs initial node position is fixed using the previous MST (as a result, if MPI is in use, this value is forced to false).

- string path

  Input and output path.

- string url

  URL for image maps.

- double width

  Width of the images.

- double height

  Height of the images.

- double pwidth

  Width of the preview images.

- double pheight

  Height of the preview images.

- enum FILETYPE outtype

  Type of output file.

- unsigned int percent

  Percentage of images to generate.

- unsigned int dpi

  The resolution (dots per inch) to use in the MST.

- bool spline_flag

  Set to true if lines should not cross in the MST (takes more time).

- unsigned int fontsize

  Size of the fonts to use.

- vector< VERTEX > vertices

  Record the vertex attributes of the current workunit.

- vector< EDGE > edges

  Record the edges of the current workunit.

- unsigned int total_iter

  Total number of iterations.

- string scores_fn

  Scores filename.

- vector< SCORE > scores

  The vector of scores.

- vector< vector< unsigned int > > all_workunits

All of the workunits as stored by the main processor only (not used by child processors).

- vector< unsigned int > my_workunit

  The workunit for processing by the current processor.

- unsigned int rank

  Rank of this process.

- unsigned int world_size

  Total number of processes.

3.2.1 Detailed Description

The LAYOUTMST class is the main class for this program. The main driver (in main.cpp) creates an instance of this class as the first task. It is also the last class destroyed before the program exits.

3.2.2 Constructor & Destructor Documentation

3.2.2.1 LAYOUTMST::LAYOUTMST ()

Default constructor that takes no arguments.

3.2.3 Member Function Documentation

3.2.3.1 bool LAYOUTMST::processOptions (int *argc*, char ∗ *argv*[ ])

Process options from the command line and the configuration file CFG_FILENAME.

This function makes use of Boost's program_options for handling arguments on the command line and in options in a configuration file whose format resembles .ini files.

Initially, boolean and enumerated values are given default values. Then, the available options are set up, with default values for string and numeric types. The description of the options are recorded.

Next, the command line options are read, followed by the configuration file options. The command line options take priority over the configuration file ones. Then, the options are processed, one-by-one.

All of this is encapsulated within a try...catch block.

Here is the call graph for this function:

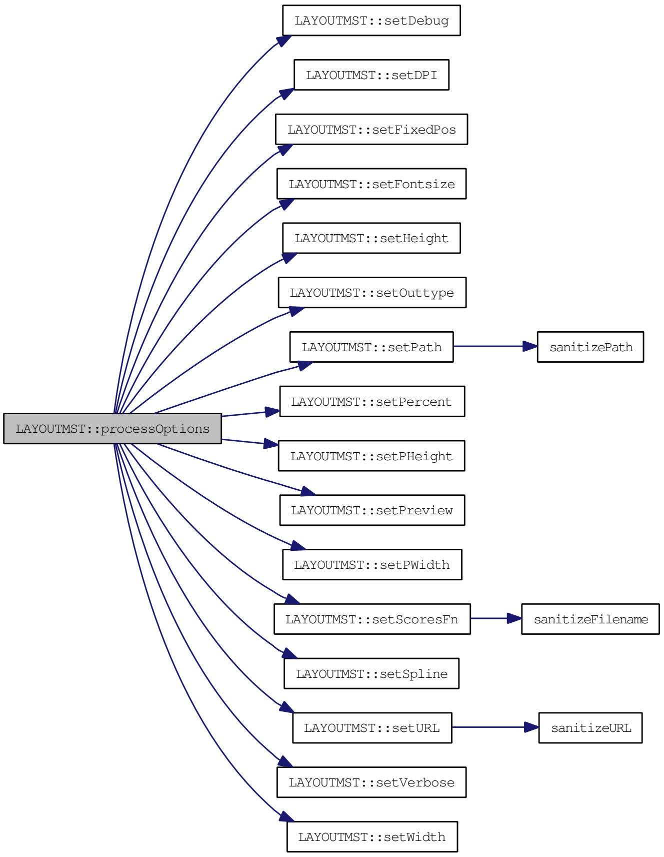

3.2.3.2 void LAYOUTMST::initSettings ()

Initialize settings when there is no MPI available.

If MPI is unavailable, then the rank of this process is 0 and the size of the "world" is 1.

Here is the call graph for this function:

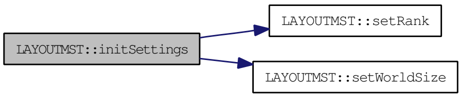

3.2.3.3 bool LAYOUTMST::checkSettings ()

Check the settings to ensure they are valid.

If no distance or linkage is set, then Euclidean and single linkage are assigned by default. If –verbose has been set, then the options that the user chose are printed out to STDERR.

Here is the call graph for this function:

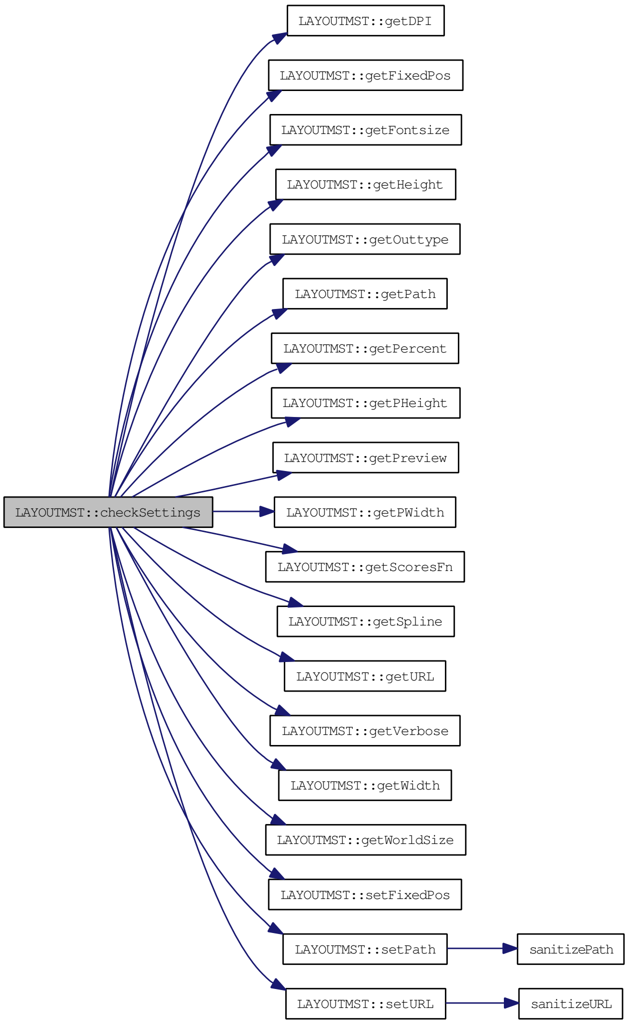

3.2.3.4 void LAYOUTMST::sendOKFail (bool *arg*)

Send the initial signal to all processes to tell them to continue or not.

Parameters:

arg Indicate whether or not sub-processes should proceed (true - yes; false - no)

Here is the call graph for this function:

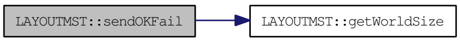

3.2.3.5 bool LAYOUTMST::recvOKFail ()

Receive the initial signal from the primary process to determine whether to continue or not.

3.2.3.6 bool LAYOUTMST::runPrimary ()

Execute the part of the program that is for the primary processor only.

Here is the call graph for this function:

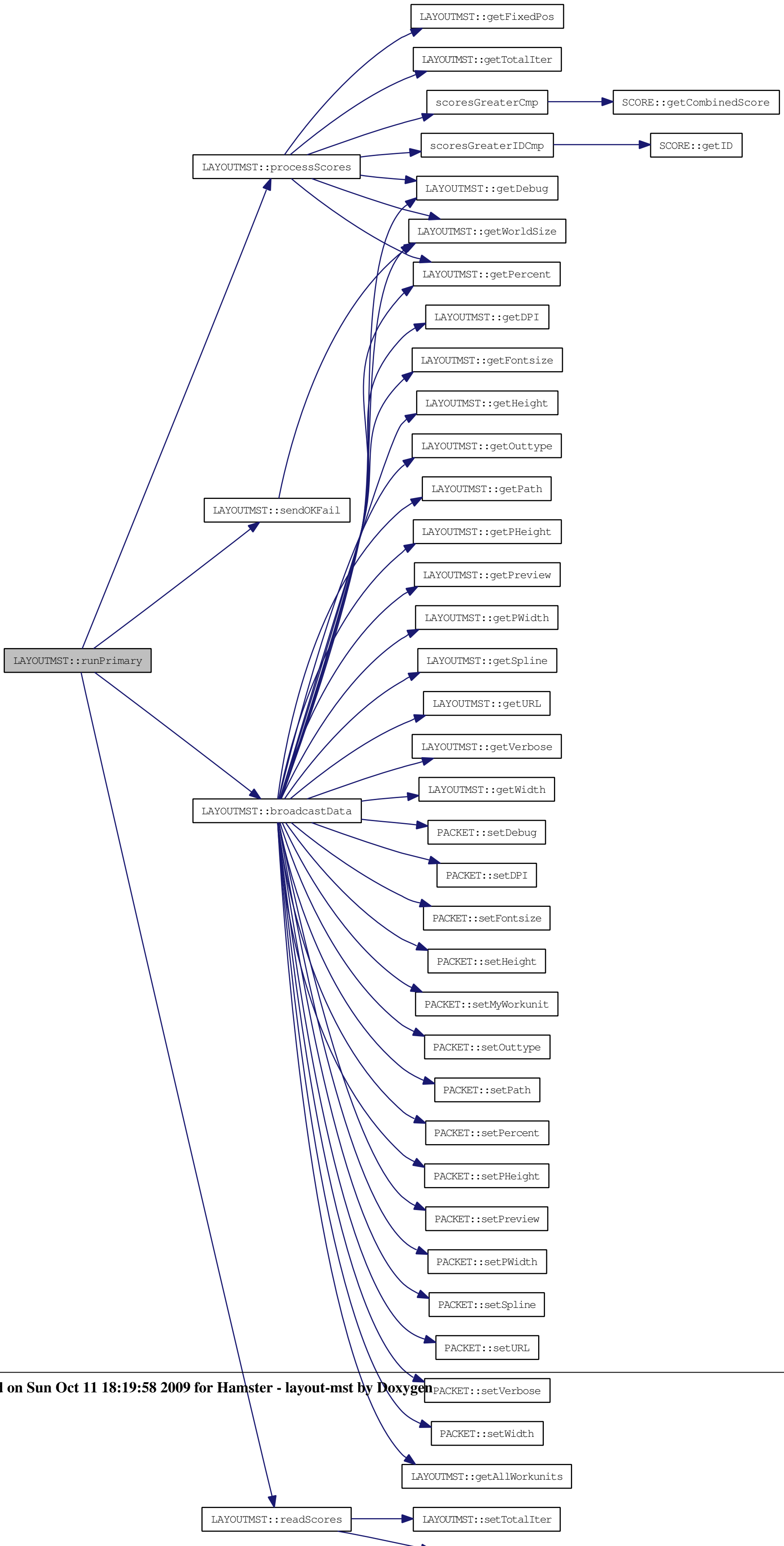

3.2.3.7 void LAYOUTMST::runAllProcessors ()

Execute the part of the program that is for all processors (including the primary).

Here is the call graph for this function:

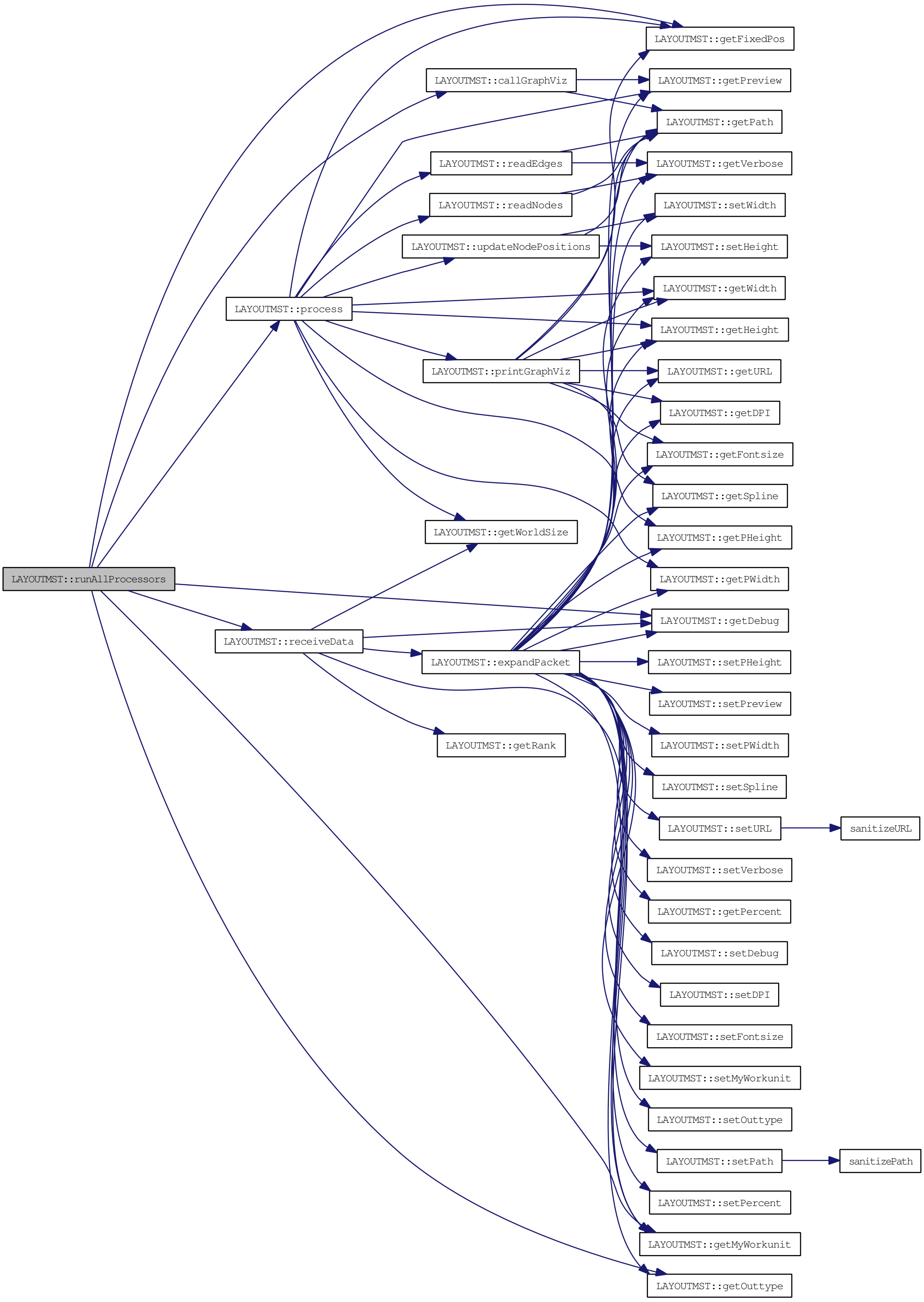

3.2.3.8 bool LAYOUTMST::readScores ()

Read the data file of scores in.

The data file must be tab-separated with an experiment on each line (assuming the user is building MSTs on the experiments). The first row and column are headers and are basically ignored. All other fields must be either floating point values or the string NULL.

Each row in the data file translates into a VECT object.

Here is the call graph for this function:

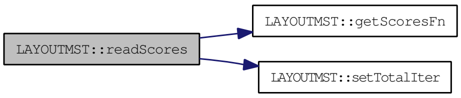

3.2.3.9 void LAYOUTMST::processScores ()

Process the scores by sorting and inserting into separate lists.

The jobs (MST IDs) are sorted by increasing score and then assigned to each process in a round-robin fashion. This ensures that if there are k processors available, the k most important MSTs are processed first simultaneously by all processors. Then, the next k most important MSTs are done, etc.

Here is the call graph for this function:

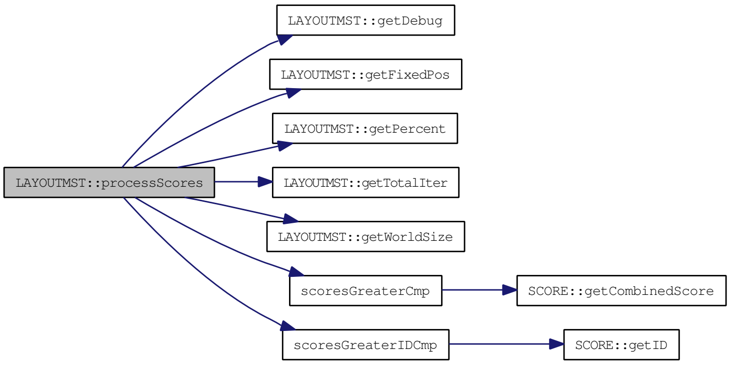

3.2.3.10 void LAYOUTMST::expandPacket (PACKET ∗ *p*)

Expand the information in a PACKET object and set the private variables.

Here is the call graph for this function:

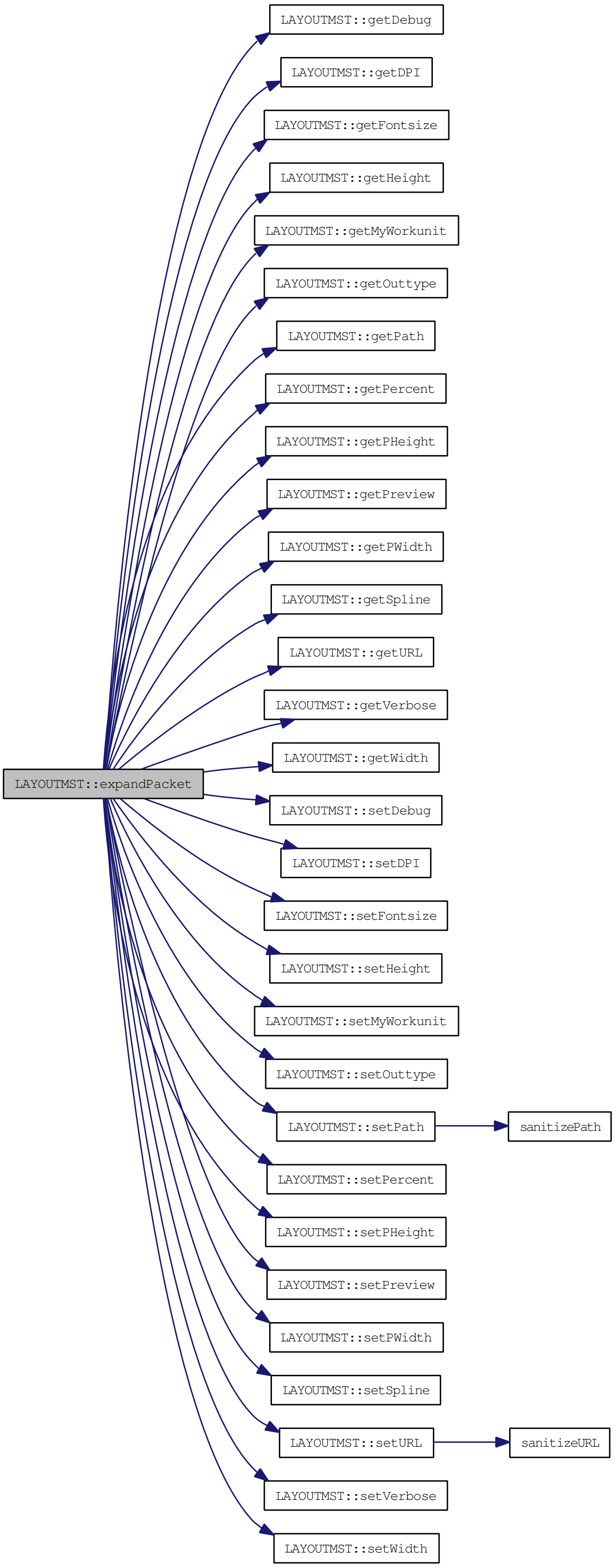

3.2.3.11 void LAYOUTMST::broadcastData ()

Construct PACKET objects for each processor and send it to them.

PACKET objects are delivered to each process; in case MPI is unavailable, then all of the workunits are simply copied from one vector (all_workunits[0]) to another (my_workunit)

Here is the call graph for this function:

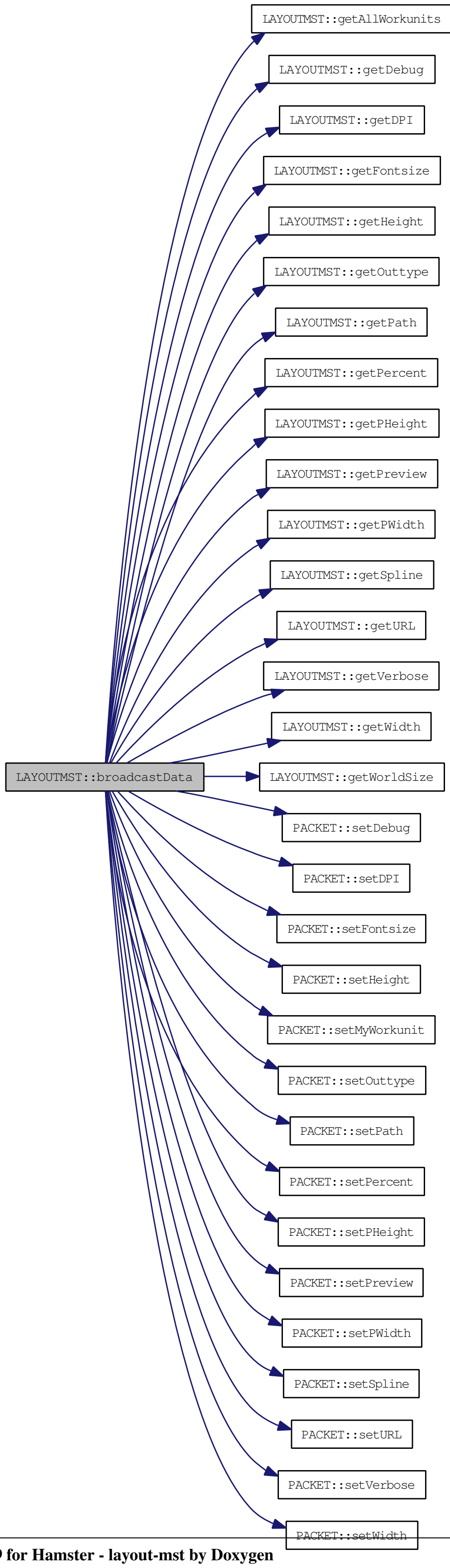

3.2.3.12 void LAYOUTMST::receiveData ()

Receive PACKET objects from the primary processor.

A PACKET object is received from the primary processor; in case MPI is unavailable, then nothing is done. Instead, the copying was accomplished by broadcastData () already.

Here is the call graph for this function:

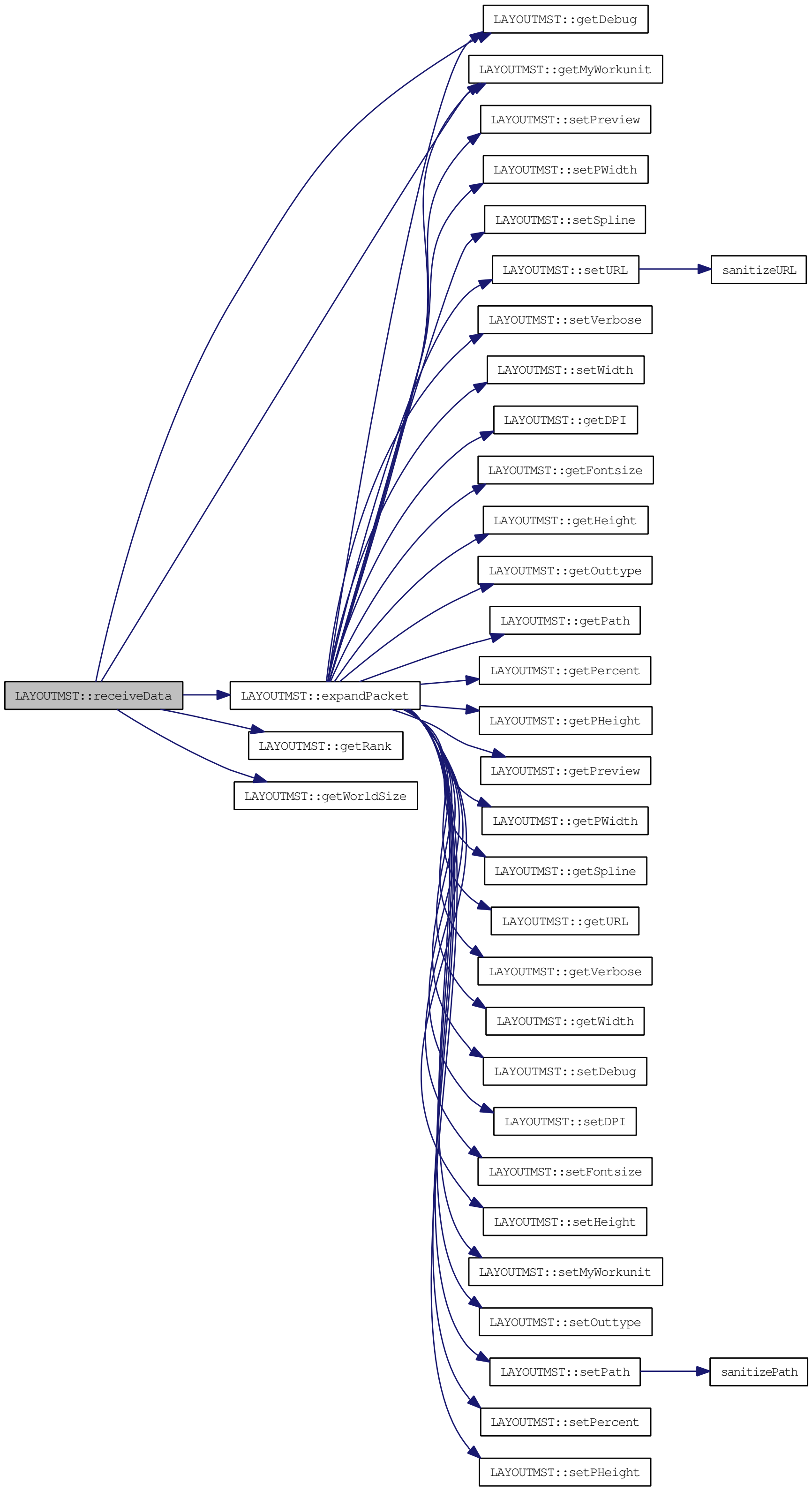

3.2.3.13 void LAYOUTMST::callGraphViz (unsigned int *id*, enum FILETYPE *in_ftype*, enum FILETYPE *out_ftype*)

Call GraphViz by executing it as a shell command.

This function calls GraphViz (the program defined in config.h as GRAPHVIZ_PATH) using "system". If GraphViz has not been installed, then this function essentially does nothing.

Parameters:

id ID of the output file we are generating

in_ftype Type of the input file (only 2 possibilities: DOT or GV)

out_ftype Type of the output file

Here is the call graph for this function:

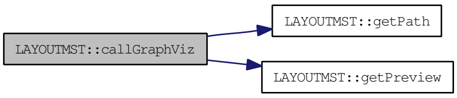

3.2.3.14 void LAYOUTMST::process (unsigned int *id*)

Process a single MST.

Parameters:

id ID of the MST (0-based)

This function processes (creates) a single MST, which is assigned a unique integral id. For each MST, the following is done:

1) Read in the corresponding nodes file. 2) Read in the corresponding edges file. 3) Read in the previous MST's nodes and edges. 4) Normalize edge weights by the largest weight smaller than DBL_MAX. 5) Output this information in GraphViz format by calling printGraphViz ().

Here is the call graph for this function:

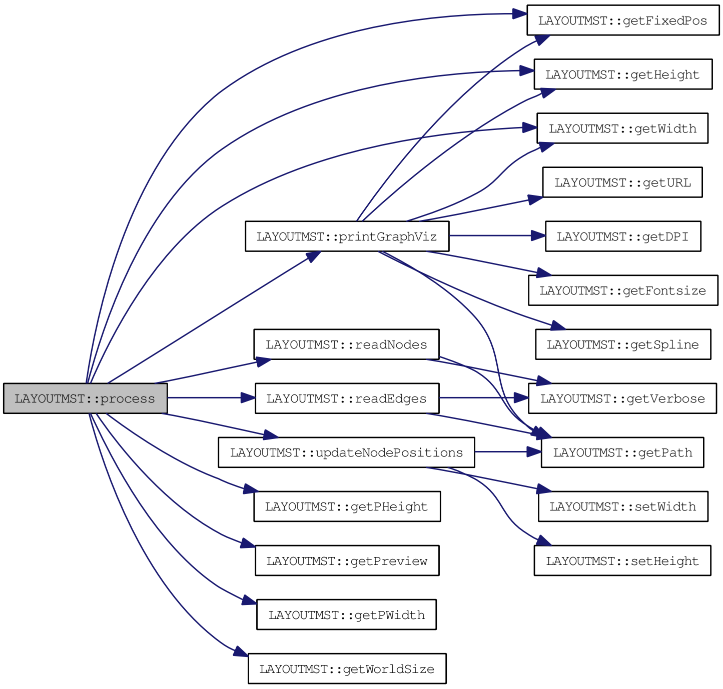

3.2.3.15 void LAYOUTMST::printGraphViz (unsigned int *id*, double *width*, double *height*, string *extension*, vector< VERTEX > *vertices*, vector< EDGE > *edges*)

Output the graph information in GraphViz format.

The function has been generalized so that it can print the actual MSTs or the preview (smaller) ones depending on the arguments given to it.

Here is the call graph for this function:

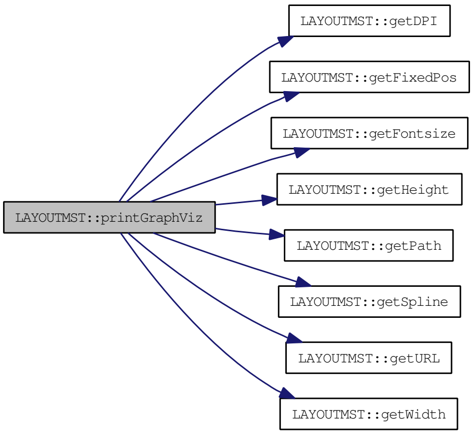

3.2.3.16 void LAYOUTMST::readNodes (unsigned int *id*)

Read in the nodes from file.

Parameters:

id ID of the MST (0-based)

Here is the call graph for this function:

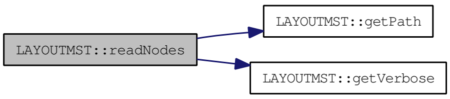

3.2.3.17 double LAYOUTMST::readEdges (unsigned int *id*)

Read in the edges from file.

Parameters:

id ID of the MST (0-based)

Here is the call graph for this function:

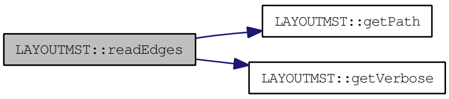

3.2.3.18 void LAYOUTMST::updateNodePositions (unsigned int *id*)

Update the node positions.

Parameters:

id ID of the MST (0-based)

Read in the previous MST and add positions to each node. If the current MST is 0, then immediately return.

Here is the call graph for this function:

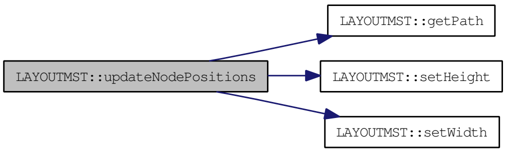

3.2.3.19 void LAYOUTMST::setDebug (bool *arg*)

Set whether or not debugging output is required

3.2.3.20 bool LAYOUTMST::getDebug () const

Get the debug setting.

3.2.3.21 void LAYOUTMST::setVerbose (bool *arg*)

Set whether or not verbose output is required.

3.2.3.22 bool LAYOUTMST::getVerbose () const

Get the verbose setting.

3.2.3.23 void LAYOUTMST::setPreview (bool *arg*)

Set whether or not preview images are required.

3.2.3.24 bool LAYOUTMST::getPreview () const

Get the preview setting.

3.2.3.25 void LAYOUTMST::setFixedPos (bool *arg*)

Set whether or not node positions are fixed using the previous MST.

3.2.3.26 bool LAYOUTMST::getFixedPos () const

Get the fixed node position setting.

3.2.3.27 void LAYOUTMST::setPath (string *arg*)

Set the input/output path (the path where files are).

Here is the call graph for this function:

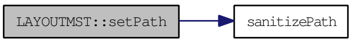

3.2.3.28 string LAYOUTMST::getPath () const

Get the output path.

3.2.3.29 void LAYOUTMST::setURL (string *arg*)

Set the URL for image maps.

Here is the call graph for this function:

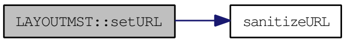

3.2.3.30 string LAYOUTMST::getURL () const

Get the URL.

3.2.3.31 void LAYOUTMST::setWidth (double *arg*)

Set the width of the images.

3.2.3.32 double LAYOUTMST::getWidth () const

Get the width of the images.

3.2.3.33 void LAYOUTMST::setHeight (double *arg*)

Set the height of the images.

3.2.3.34 double LAYOUTMST::getHeight () const

Get the height of the images.

3.2.3.35 void LAYOUTMST::setPWidth (double *arg*)

Set the width of the preview images.

3.2.3.36 double LAYOUTMST::getPWidth () const

Get the width of the preview images.

3.2.3.37 void LAYOUTMST::setPHeight (double *arg*)

Set the height of the preview images.

3.2.3.38 double LAYOUTMST::getPHeight () const

Get the height of the preview images.

3.2.3.39 void LAYOUTMST::setOuttype (enum FILETYPE *arg*)

Set the type of output image.

3.2.3.40 enum FILETYPE LAYOUTMST::getOuttype () const

Get the type of output image.

3.2.3.41 void LAYOUTMST::setPercent (unsigned int *arg*)

Set the percent.

3.2.3.42 unsigned int LAYOUTMST::getPercent () const

Get the percent.

3.2.3.43 void LAYOUTMST::setDPI (unsigned int *arg*)

Set the DPI.

3.2.3.44 unsigned int LAYOUTMST::getDPI () const

Get the DPI.

3.2.3.45 void LAYOUTMST::setSpline (bool *arg*)

Set the spline flag.

3.2.3.46 bool LAYOUTMST::getSpline () const

Get the spline flag.

3.2.3.47 void LAYOUTMST::setFontsize (unsigned int *arg*)

Set the font size.

3.2.3.48 unsigned int LAYOUTMST::getFontsize () const

Get the font size.

3.2.3.49 void LAYOUTMST::setTotalIter (unsigned int *arg*)

Set the total number of iterations

3.2.3.50 unsigned int LAYOUTMST::getTotalIter () const

Get the total number of iterations.

3.2.3.51 void LAYOUTMST::setScoresFn (string *arg*)

Set the scores filename.

Here is the call graph for this function:

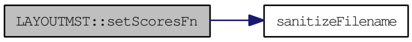

3.2.3.52 string LAYOUTMST::getScoresFn () const

Get the scores filename.

3.2.3.53 void LAYOUTMST::setMyWorkunit (vector< unsigned int > *arg*)

Set the workunits for the current process to do

3.2.3.54 vector< unsigned int > LAYOUTMST::getMyWorkunit () const

Get the workunits for the current process to do.

3.2.3.55 void LAYOUTMST::setAllWorkunits (unsigned int *arg1*, vector< unsigned int > *arg2*)

Set the workunits for the child process arg1 to do (used by the primary processor only).

Parameters:

arg1 The rank of the process that will get this workunit

arg2 The workunit

3.2.3.56 vector< unsigned int > LAYOUTMST::getAllWorkunits (unsigned int *arg*) const

Get the workunits for the child process arg1 to do from the primary processor.

Parameters:

arg The rank of the process that will get this workunit

3.2.3.57 void LAYOUTMST::setRank (unsigned int *arg*)

Set the rank for the current process.

3.2.3.58 unsigned int LAYOUTMST::getRank () const

Get the rank for the current process.

3.2.3.59 void LAYOUTMST::setWorldSize (unsigned int *arg*)

Get the total number of processes.

3.2.3.60 unsigned int LAYOUTMST::getWorldSize () const

Set the total number of processes.

3.2.4 Member Data Documentation

3.2.4.1 bool LAYOUTMST::debug_flag `[private]`

Set to true if debug output is required; false otherwise.

3.2.4.2 bool LAYOUTMST::verbose_flag `[private]`

Set to true if verbose output is required; false otherwise.

3.2.4.3 bool LAYOUTMST::preview_flag `[private]`

Set to true if smaller, preview images are required; false otherwise.

3.2.4.4 bool LAYOUTMST::fixed_pos `[private]`

Set to true if each MSTs initial node position is fixed using the previous MST (as a result, if MPI is in use, this value is forced to false).

3.2.4.5 string LAYOUTMST::path `[private]`

Input and output path.

3.2.4.6 string LAYOUTMST::url `[private]`

URL for image maps.

3.2.4.7 double LAYOUTMST::width `[private]`

Width of the images.

3.2.4.8 double LAYOUTMST::height `[private]`

Height of the images.

3.2.4.9 double LAYOUTMST::pwidth [private]

Width of the preview images.

3.2.4.10 double LAYOUTMST::pheight [private]

Height of the preview images.

3.2.4.11 enum FILETYPE LAYOUTMST::outtype [private]

Type of output file.

3.2.4.12 unsigned int LAYOUTMST::percent [private]

Percentage of images to generate.

3.2.4.13 unsigned int LAYOUTMST::dpi [private]

The resolution (dots per inch) to use in the MST.

3.2.4.14 bool LAYOUTMST::spline_flag [private]

Set to true if lines should not cross in the MST (takes more time).

3.2.4.15 unsigned int LAYOUTMST::fontsize [private]

Size of the fonts to use.

3.2.4.16 vector<VERTEX> LAYOUTMST::vertices [private]

Record the vertex attributes of the current workunit.

3.2.4.17 vector<EDGE> LAYOUTMST::edges [private]

Record the edges of the current workunit.

3.2.4.18 unsigned int LAYOUTMST::total_iter [private]

Total number of iterations.

3.2.4.19 string LAYOUTMST::scores_fn [private]

Scores filename.

3.2.4.20 vector<SCORE> LAYOUTMST::scores [private]

The vector of scores.

3.2.4.21 vector< vector<unsigned int> > LAYOUTMST::all_workunits [private]

All of the workunits as stored by the main processor only (not used by child processors).

3.2.4.22 vector< unsigned int > LAYOUTMST::my_workunit [private]

The workunit for processing by the current processor.

3.2.4.23 unsigned int LAYOUTMST::rank [private]

Rank of this process.

3.2.4.24 unsigned int LAYOUTMST::world_size [private]

Total number of processes.

The documentation for this class was generated from the following files:

- layout_mst.h
- graphviz.cpp
- io.cpp
- layout_mst.cpp
- parameters.cpp
- process_scores.cpp
- run.cpp
- transmit.cpp

3.3 PACKET Class Reference

```
#include <packet.h>
```

Public Member Functions

- PACKET ()

  Default constructor that takes no arguments.

- void setDebug (bool arg)

  Set whether or not debugging output is required.

- bool getDebug () const

  Get the debug setting.

- void setVerbose (bool arg)

  Set whether or not verbose output is required.

- bool getVerbose () const

  Get the verbose setting.

- void setPreview (bool arg)

  Set whether or not verbose output is required.

- bool getPreview () const

  Get the verbose setting.

- void setFixedPos (bool arg)

  Set whether or not node positions are fixed using the previous MST.

- bool getFixedPos () const

  Get the fixed node position setting.

- void setPath (string arg)

  Set the path to the files.

- string getPath () const

  Get the path.

- void setURL (string arg)

  Set the URL for image maps.

- string getURL () const

  Get the URL.

- void setWidth (double arg)

  Set the width of the images.

- double getWidth () const

Get the width of the images.

- void setHeight (double arg)

    Set the height of the images.

- double getHeight () const

    Get the height of the images.

- void setPWidth (double arg)

    Set the width of the preview images.

- double getPWidth () const

    Get the width of the preview images.

- void setPHeight (double arg)

    Set the height of the preview images.

- double getPHeight () const

    Get the height of the preview images.

- void setOuttype (enum FILETYPE)

    Set the type of output image.

- enum FILETYPE getOuttype () const

    Get the type of output image.

- void setPercent (unsigned int arg)

    Set the percent.

- unsigned int getPercent () const

    Get the percent.

- void setDPI (unsigned int arg)

    Set the DPI.

- unsigned int getDPI () const

    Get the DPI.

- void setSpline (bool arg)

    Set the spline flag.

- bool getSpline () const

    Get the spline flag.

- void setFontsize (unsigned int arg)

    Set the font size.

- unsigned int getFontsize () const

    Get the font size.

- void setMyWorkunit (vector< unsigned int > arg)

  Set the workunits for the current process to do.

- vector< unsigned int > getMyWorkunit () const

  Get the workunits for the current process to do.

Private Attributes

- bool debug_flag

  Set to true if debug output is required; false otherwise.

- bool verbose_flag

  Set to true if verbose output is required; false otherwise.

- bool preview_flag

  Set to true if smaller, preview images are required; false otherwise.

- bool fixed_pos

  Set to true if each MSTs initial node position is fixed using the previous MST (as a result, if MPI is in use, this value is forced to false).

- string path

  Path to files.

- string url

  URL for image maps.

- double width

  Width of the images.

- double height

  Height of the images.

- double pwidth

  Width of the preview images.

- double pheight

  Height of the preview images.

- enum FILETYPE outtype

  Type of output file.

- unsigned int percent

  Percent of images to generate.

- unsigned int dpi

  The resolution (dots per inch) to use in the MST.

- bool spline_flag

Set to true if lines should not cross in the MST (takes more time).

- unsigned int fontsize

  Size of the fonts to use.

- vector< unsigned int > my_workunit

  The workunit that the current process has to worry about.

3.3.1 Detailed Description

A PACKET represents the object with all of the variables that each child process needs from the main, primary process to do its work. They have been collected together into a class to facilitate the Boost Serialization class.

Many of the variables are duplicated from the LAYOUTMST class.

3.3.2 Constructor & Destructor Documentation

3.3.2.1 PACKET::PACKET ()

Default constructor that takes no arguments.

3.3.3 Member Function Documentation

3.3.3.1 void PACKET::setDebug (bool *arg*)

Set whether or not debugging output is required.

3.3.3.2 bool PACKET::getDebug () const

Get the debug setting.

3.3.3.3 void PACKET::setVerbose (bool *arg*)

Set whether or not verbose output is required.

3.3.3.4 bool PACKET::getVerbose () const

Get the verbose setting.

3.3.3.5 void PACKET::setPreview (bool *arg*)

Set whether or not verbose output is required.

3.3.3.6 bool PACKET::getPreview () const

Get the verbose setting.

3.3.3.7 void PACKET::setFixedPos (bool *arg*)

Set whether or not node positions are fixed using the previous MST.

3.3.3.8 bool PACKET::getFixedPos () const

Get the fixed node position setting.

3.3.3.9 void PACKET::setPath (string *arg*)

Set the path to the files.

3.3.3.10 string PACKET::getPath () const

Get the path.

3.3.3.11 void PACKET::setURL (string *arg*)

Set the URL for image maps.

3.3.3.12 string PACKET::getURL () const

Get the URL.

3.3.3.13 void PACKET::setWidth (double *arg*)

Set the width of the images.

3.3.3.14 double PACKET::getWidth () const

Get the width of the images.

3.3.3.15 void PACKET::setHeight (double *arg*)

Set the height of the images.

3.3.3.16 double PACKET::getHeight () const

Get the height of the images.

3.3.3.17 void PACKET::setPWidth (double *arg*)

Set the width of the preview images.

3.3.3.18 double PACKET::getPWidth () const

Get the width of the preview images.

3.3.3.19 void PACKET::setPHeight (double *arg*)

Set the height of the preview images.

3.3.3.20 double PACKET::getPHeight () const

Get the height of the preview images.

3.3.3.21 void PACKET::setOuttype (enum FILETYPE *arg*)

Set the type of output image.

3.3.3.22 enum FILETYPE PACKET::getOuttype () const

Get the type of output image.

3.3.3.23 void PACKET::setPercent (unsigned int *arg*)

Set the percent.

3.3.3.24 unsigned int PACKET::getPercent () const

Get the percent.

3.3.3.25 void PACKET::setDPI (unsigned int *arg*)

Set the DPI.

3.3.3.26 unsigned int PACKET::getDPI () const

Get the DPI.

3.3.3.27 void PACKET::setSpline (bool *arg*)

Set the spline flag.

3.3.3.28 bool PACKET::getSpline () const

Get the spline flag.

3.3.3.29 void PACKET::setFontsize (unsigned int *arg*)

Set the font size.

3.3.3.30 unsigned int PACKET::getFontsize () const

Get the font size.

3.3.3.31 void PACKET::setMyWorkunit (vector< unsigned int > *arg*)

Set the workunits for the current process to do.

3.3.3.32 vector< unsigned int > PACKET::getMyWorkunit () const

Get the workunits for the current process to do.

3.3.4 Member Data Documentation

3.3.4.1 bool PACKET::debug_flag `[private]`

Set to true if debug output is required; false otherwise.

3.3.4.2 bool PACKET::verbose_flag `[private]`

Set to true if verbose output is required; false otherwise.

3.3.4.3 bool PACKET::preview_flag `[private]`

Set to true if smaller, preview images are required; false otherwise.

3.3.4.4 bool PACKET::fixed_pos `[private]`

Set to true if each MSTs initial node position is fixed using the previous MST (as a result, if MPI is in use, this value is forced to false).

3.3.4.5 string PACKET::path `[private]`

Path to files.

3.3.4.6 string PACKET::url `[private]`

URL for image maps.

3.3.4.7 double PACKET::width `[private]`

Width of the images.

3.3.4.8 double PACKET::height [private]

Height of the images.

3.3.4.9 double PACKET::pwidth [private]

Width of the preview images.

3.3.4.10 double PACKET::pheight [private]

Height of the preview images.

3.3.4.11 enum FILETYPE PACKET::outtype [private]

Type of output file.

3.3.4.12 unsigned int PACKET::percent [private]

Percent of images to generate.

3.3.4.13 unsigned int PACKET::dpi [private]

The resolution (dots per inch) to use in the MST.

3.3.4.14 bool PACKET::spline_flag [private]

Set to true if lines should not cross in the MST (takes more time).

3.3.4.15 unsigned int PACKET::fontsize [private]

Size of the fonts to use.

3.3.4.16 vector< unsigned int > PACKET::my_workunit [private]

The workunit that the current process has to worry about.

The documentation for this class was generated from the following files:

- packet.h
- packet.cpp

3.4 SCORE Class Reference

`#include <score.h>`

Public Member Functions

- SCORE ()
  Constructor that takes no arguments.
- SCORE (unsigned int arg1, unsigned int arg2, unsigned int arg3, double arg4, double arg5, double arg6)
  Constructor that takes five arguments.
- void setID (unsigned int arg)
  Set the ID.
- void setLeft (unsigned int arg)
  Set the ID of the left cluster.
- void setRight (unsigned int arg)
  Set the ID of the right cluster associated with this score.
- void setScore1 (double arg)
  Set score 1.
- void setScore2 (double arg)
  Set score 2.
- void setCombinedScore (double arg)
  Set combined score.
- unsigned int getID () const
  Get the ID.
- unsigned int getLeft () const
  Get the ID of the left cluster.
- unsigned int getRight () const
  Get the ID of the right cluster.
- double getScore1 () const
  Get score 1.
- double getScore2 () const
  Get score 2.
- double getCombinedScore () const
  Get combined score.

- bool operator< (const SCORE &arg) const

  Overloaded operator for SCORE nodes (less than).

- bool operator> (const SCORE &arg) const

  Overloaded operator for SCORE nodes (greater than).

Private Attributes

- unsigned int id

  The merge ID, numbered from 0.

- unsigned int left

  The left cluster in the merge.

- unsigned int right

  The right cluster in the merge.

- double score1

  The intra-cluster score (within-cluster) or the mean square for groups.

- double score2

  The inter-cluster score (between-cluster) or the mean square error.

- double combined

  The combined score calculated by either subtracting or dividing score1 and score2.

3.4.1 Detailed Description

A SCORE node keeps track of the intra and inter-cluster scores for a particular merge. In addition to these scores, information about the merge (its unique integral ID and the IDs of the two clusters that were merged) are also kept track of

3.4.2 Constructor & Destructor Documentation

3.4.2.1 SCORE::SCORE ()

Constructor that takes no arguments.

3.4.2.2 SCORE::SCORE (unsigned int *arg1*, unsigned int *arg2*, unsigned int *arg3*, double *arg4*, double *arg5*, double *arg6*)

Constructor that takes five arguments.

Parameters:

arg1 ID of the node

arg2 ID of the left cluster

arg3 ID of the right cluster
arg4 Score 1
arg5 Score 2
arg6 Combined score

3.4.3 Member Function Documentation

3.4.3.1 void SCORE::setID (unsigned int *arg*)

Set the ID.

3.4.3.2 void SCORE::setLeft (unsigned int *arg*)

Set the ID of the left cluster.

3.4.3.3 void SCORE::setRight (unsigned int *arg*)

Set the ID of the right cluster associated with this score.

3.4.3.4 void SCORE::setScore1 (double *arg*)

Set score 1.

3.4.3.5 void SCORE::setScore2 (double *arg*)

Set score 2.

3.4.3.6 void SCORE::setCombinedScore (double *arg*)

Set combined score.

3.4.3.7 unsigned int SCORE::getID () const

Get the ID.

3.4.3.8 unsigned int SCORE::getLeft () const

Get the ID of the left cluster.

3.4.3.9 unsigned int SCORE::getRight () const

Get the ID of the right cluster.

3.4.3.10 double SCORE::getScore1 () const

Get score 1.

3.4.3.11 double SCORE::getScore2 () const

Get score 2.

3.4.3.12 double SCORE::getCombinedScore () const

Get combined score.

3.4.3.13 bool SCORE::operator< (const SCORE & *arg*) const

Overloaded operator for SCORE nodes (less than).

3.4.3.14 bool SCORE::operator> (const SCORE & *arg*) const

Overloaded operator for SCORE nodes (greater than).

3.4.4 Member Data Documentation

3.4.4.1 unsigned int SCORE::id `[private]`

The merge ID, numbered from 0.

3.4.4.2 unsigned int SCORE::left `[private]`

The left cluster in the merge.

3.4.4.3 unsigned int SCORE::right `[private]`

The right cluster in the merge.

3.4.4.4 double SCORE::score1 `[private]`

The intra-cluster score (within-cluster) or the mean square for groups.

3.4.4.5 double SCORE::score2 `[private]`

The inter-cluster score (between-cluster) or the mean square error.

3.4.4.6 double SCORE::combined `[private]`

The combined score calculated by either subtracting or dividing score1 and score2.

The documentation for this class was generated from the following files:

- score.h
- score.cpp

3.5 VERTEX Class Reference

```
#include <vertex.h>
```

Public Member Functions

- VERTEX ()

  Default constructor that takes no arguments.
- VERTEX (string arg1, string arg2, string arg3, unsigned int arg4)

  Default constructor that takes four arguments.
- void setName (string arg)

  Set the vertex name.
- string getName () const

  Get the vertex name.
- void setColour (string arg)

  Set the vertex colour.
- string getColour () const

  Get the vertex colour.
- void setShape (string arg)

  Set the vertex shape.
- string getShape () const

  Get the vertex shape.
- void setComponents (unsigned int arg)

  Set the number of components.
- unsigned int getComponents () const

  Get the number of components.
- void setUpdated (bool arg)

  Change the update status of the object.
- bool getUpdated () const

  Check if the object has its coordinates updated.
- void setX (unsigned int arg)

  Set the x coordinate.
- unsigned int getX () const

  Get the x coordinate.
- void setY (unsigned int arg)

Set the y coordinate.

- unsigned int getY () const

  Get the y coordinate.

- void setWidth (float arg)

  Set the node width.

- float getWidth () const

  Get the node width.

- void setHeight (float arg)

  Set the node height.

- float getHeight () const

  Get the node height.

Private Attributes

- string name

  Vertex name.

- string colour

  Vertex colour.

- string shape

  Vertex shape.

- bool updated

  Updated with node positions?

- unsigned int components

  Number of components.

- unsigned int x

  x position

- unsigned int y

  y position

- float width

  Node width.

- float height

  Node height.

3.5.1 Detailed Description

A VERTEX contains three attributes: name, colour, and shape.

3.5.2 Constructor & Destructor Documentation

3.5.2.1 VERTEX::VERTEX ()

Default constructor that takes no arguments.

3.5.2.2 VERTEX::VERTEX (string *arg1*, string *arg2*, string *arg3*, unsigned int *arg4*)

Default constructor that takes four arguments.

Parameters:

arg1 Vertex name

arg2 Vertex colour

arg3 Vertex shape

arg4 Number of components in node

3.5.3 Member Function Documentation

3.5.3.1 void VERTEX::setName (string *arg*)

Set the vertex name.

3.5.3.2 string VERTEX::getName () const

Get the vertex name.

3.5.3.3 void VERTEX::setColour (string *arg*)

Set the vertex colour.

3.5.3.4 string VERTEX::getColour () const

Get the vertex colour.

3.5.3.5 void VERTEX::setShape (string *arg*)

Set the vertex shape.

3.5.3.6 string VERTEX::getShape () const

Get the vertex shape.

3.5.3.7 void VERTEX::setComponents (unsigned int *arg*)

Set the number of components.

3.5.3.8 unsigned int VERTEX::getComponents () const

Get the number of components.

3.5.3.9 void VERTEX::setUpdated (bool *arg*)

Change the update status of the object.

3.5.3.10 bool VERTEX::getUpdated () const

Check if the object has its coordinates updated.

3.5.3.11 void VERTEX::setX (unsigned int *arg*)

Set the x coordinate.

3.5.3.12 unsigned int VERTEX::getX () const

Get the x coordinate.

3.5.3.13 void VERTEX::setY (unsigned int *arg*)

Set the y coordinate.

3.5.3.14 unsigned int VERTEX::getY () const

Get the y coordinate.

3.5.3.15 void VERTEX::setWidth (float *arg*)

Set the node width.

3.5.3.16 float VERTEX::getWidth () const

Get the node width.

3.5.3.17 void VERTEX::setHeight (float *arg*)

Set the node height.

3.5.3.18 float VERTEX::getHeight () const

Get the node height.

3.5.4 Member Data Documentation

3.5.4.1 string VERTEX::name [private]

Vertex name.

3.5.4.2 string VERTEX::colour [private]

Vertex colour.

3.5.4.3 string VERTEX::shape [private]

Vertex shape.

3.5.4.4 bool VERTEX::updated [private]

Updated with node positions?

3.5.4.5 unsigned int VERTEX::components [private]

Number of components.

3.5.4.6 unsigned int VERTEX::x [private]

x position

3.5.4.7 unsigned int VERTEX::y [private]

y position

3.5.4.8 float VERTEX::width [private]

Node width.

3.5.4.9 float VERTEX::height [private]

Node height.

The documentation for this class was generated from the following files:

- vertex.h
- vertex.cpp

Chapter 4

File Documentation

4.1 check.cpp File Reference

```
#include <string>
#include <cctype>
#include "check.h"
```

Include dependency graph for check.cpp:

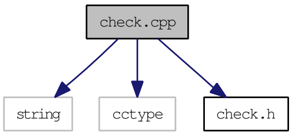

Functions

- string sanitizeFilename (string arg)

  Sanitize a filename by disallowing [/\].

- string sanitizePath (string arg)

  Sanitize a path by allowing alphanumeric characters and [./].

- string sanitizeURL (string arg)

  Sanitize a URL by allowing alphanumeric characters and [/:.].

4.1.1 Function Documentation

4.1.1.1 string sanitizeFilename (string *arg*)

Sanitize a filename by disallowing [/\].

We allow all characters since the filename is not as dangerous. However, we do not allow the slash or backslash characters to prevent a change in path and any form of escaping.

4.1.1.2 string sanitizePath (string *arg*)

Sanitize a path by allowing alphanumeric characters and [./].

Note that the backslash character has been purposely excluded to prevent any escaping. This will cause problems to the Windows' family of operating systems and should be added in, if required.

4.1.1.3 string sanitizeURL (string *arg*)

Sanitize a URL by allowing alphanumeric characters and [/:.].

Note that the backslash character has been purposely excluded to prevent any escaping.

4.2 check.h File Reference

This graph shows which files directly or indirectly include this file:

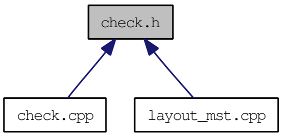

Functions

- string sanitizeFilename (string arg)

  Sanitize a filename by disallowing [/\].

- string sanitizePath (string arg)

  Sanitize a path by allowing alphanumeric characters and [./].

- string sanitizeURL (string arg)

  Sanitize a URL by allowing alphanumeric characters and [/:.].

4.2.1 Function Documentation

4.2.1.1 string sanitizeFilename (string *arg*)

Sanitize a filename by disallowing [/\].

We allow all characters since the filename is not as dangerous. However, we do not allow the slash or backslash characters to prevent a change in path and any form of escaping.

4.2.1.2 string sanitizePath (string *arg*)

Sanitize a path by allowing alphanumeric characters and [./].

Note that the backslash character has been purposely excluded to prevent any escaping. This will cause problems to the Windows' family of operating systems and should be added in, if required.

4.2.1.3 string sanitizeURL (string *arg*)

Sanitize a URL by allowing alphanumeric characters and [/:.].

Note that the backslash character has been purposely excluded to prevent any escaping.

4.3 edge.cpp File Reference

#include <string>

#include "edge.h"

Include dependency graph for edge.cpp:

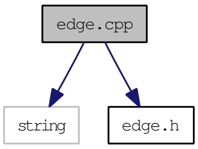

4.4 edge.h File Reference

This graph shows which files directly or indirectly include this file:

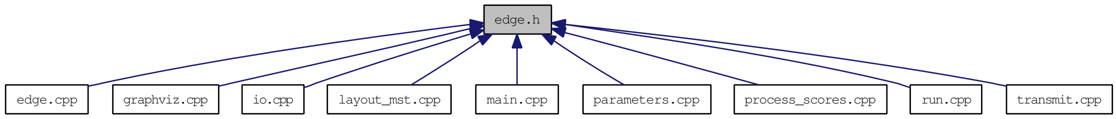

Classes

- class EDGE

4.5 global_defn.h File Reference

This graph shows which files directly or indirectly include this file:

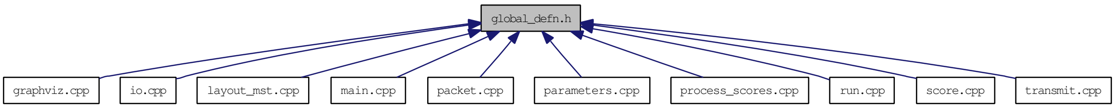

Defines

- #define VERBOSE_WIDTH 45

  Spacing for aligning the verbose output (in characters).

- #define CFG_FILENAME "layout-mst.cfg"

  The default filename for the configuration file.

- #define SCORES_FIELDS 6

  The number of fields in the file of scores.

- #define ENABLE_LARGE_NODE 0

  Set to 1 to enable large nodes (set LARGE_NODE_INCREASE and LARGE_NODE_THRESH); OFF by default!

- #define LARGE_NODE_INCREASE 2

  Size of node increase (as a multiplier).

- #define LARGE_NODE_THRESH 0.25

  Threshold for when a node increases by LARGE_NODE_INCREASE; 0.25 means that if a node has 25% of the total number of experiments, enlarge it.

- #define EDGES_FILE_EXTENSION ".edges"

  File extension for the file of edges.

- #define NODES_FILE_EXTENSION ".nodes"

  File extension for the file of nodes.

- #define DOT_FILE_EXTENSION ".dot"

  File extension for the intermediate GraphViz file.

- #define GV_FILE_EXTENSION ".graphviz"

  File extension for the GraphViz file.

- #define GVPV_FILE_EXTENSION "-pv.graphviz"

  File extension for the preview GraphViz file.

- #define DEFAULT_URL "http://localhost/"

  Default base URL.

- #define DEFAULT_WIDTH 21.0

  Default image width (cm); A4.

- #define DEFAULT_HEIGHT 29.7

  Default image height (cm); A4.

- #define DEFAULT_PWIDTH 2.6

  Default preview image width (cm); A10.

- #define DEFAULT_PHEIGHT 3.7

  Default preview image height (cm); A10.

- #define MIN_DIM 2

  Minimum dimension (cm).

- #define MAX_DIM 30

  Maximum dimension (cm).

- #define DEFAULT_PERCENT 100

  Default percentage is everything (100); this is an unsigned int.

- #define DEFAULT_DPI 96

  Default resolution (DPI).

- #define MIN_DPI 48

  Minimum resolution (DPI).

- #define MAX_DPI 900

  Maximum resolution (DPI).

- #define DEFAULT_FONTSIZE 12

  Default fontsize.

- #define MIN_FONTSIZE 8

  Minimum fontsize.

- #define MAX_FONTSIZE 24

  Maximum fontsize.

- #define SCALE_FACTOR 2.54

  Conversion from cm to inches, which is used by GraphViz.

Enumerations

- enum FILETYPE {
  FILETYPE_UNSET, FILETYPE_GV, FILETYPE_DOT, FILETYPE_PNG,
  FILETYPE_SVG, FILETYPE_CMAP, FILETYPE_PS }

  The type of output file.

4.5.1 Define Documentation

4.5.1.1 #define CFG_FILENAME "layout-mst.cfg"

The default filename for the configuration file.

4.5.1.2 #define DEFAULT_DPI 96

Default resolution (DPI).

4.5.1.3 #define DEFAULT_FONTSIZE 12

Default fontsize.

4.5.1.4 #define DEFAULT_HEIGHT 29.7

Default image height (cm); A4.

4.5.1.5 #define DEFAULT_PERCENT 100

Default percentage is everything (100); this is an unsigned int.

4.5.1.6 #define DEFAULT_PHEIGHT 3.7

Default preview image height (cm); A10.

4.5.1.7 #define DEFAULT_PWIDTH 2.6

Default preview image width (cm); A10.

4.5.1.8 #define DEFAULT_URL "http://localhost/"

Default base URL.

4.5.1.9 #define DEFAULT_WIDTH 21.0

Default image width (cm); A4.

4.5.1.10 #define DOT_FILE_EXTENSION ".dot"

File extension for the intermediate GraphViz file.

4.5.1.11 #define EDGES_FILE_EXTENSION ".edges"

File extension for the file of edges.

4.5.1.12 #define ENABLE_LARGE_NODE 0

Set to 1 to enable large nodes (set LARGE_NODE_INCREASE and LARGE_NODE_THRESH); OFF by default!

4.5.1.13 #define GV_FILE_EXTENSION ".graphviz"

File extension for the GraphViz file.

4.5.1.14 #define GVPV_FILE_EXTENSION "-pv.graphviz"

File extension for the preview GraphViz file.

4.5.1.15 #define LARGE_NODE_INCREASE 2

Size of node increase (as a multiplier).

4.5.1.16 #define LARGE_NODE_THRESH 0.25

Threshold for when a node increases by LARGE_NODE_INCREASE; 0.25 means that if a node has 25% of the total number of experiments, enlarge it.

4.5.1.17 #define MAX_DIM 30

Maximum dimension (cm).

4.5.1.18 #define MAX_DPI 900

Maximum resolution (DPI).

4.5.1.19 #define MAX_FONTSIZE 24

Maximum fontsize.

4.5.1.20 #define MIN_DIM 2

Minimum dimension (cm).

4.5.1.21 #define MIN_DPI 48

Minimum resolution (DPI).

4.5.1.22 #define MIN_FONTSIZE 8

Minimum fontsize.

4.5.1.23 #define NODES_FILE_EXTENSION ".nodes"

File extension for the file of nodes.

4.5.1.24 #define SCALE_FACTOR 2.54

Conversion from cm to inches, which is used by GraphViz.

4.5.1.25 #define SCORES_FIELDS 6

The number of fields in the file of scores.

4.5.1.26 #define VERBOSE_WIDTH 45

Spacing for aligning the verbose output (in characters).

4.5.2 Enumeration Type Documentation

4.5.2.1 enum FILETYPE

The type of output file.

Enumerator:

FILETYPE_UNSET File type not yet set

FILETYPE_GV Final Graphviz file

FILETYPE_DOT Intermediate Graphviz/DOT file

FILETYPE_PNG PNG file

FILETYPE_SVG SVG file

FILETYPE_CMAP Client-side map file

FILETYPE_PS Postscript file

4.6 graphviz.cpp File Reference

```
#include <iostream>
#include <fstream>
#include <iomanip>
#include <string>
#include <vector>
#include <cfloat>
#include "config.h"
#include <boost/tokenizer.hpp>
#include <boost/lexical_cast.hpp>
#include "global_defn.h"
#include "packet.h"
#include "score.h"
#include "vertex.h"
#include "edge.h"
#include "layout_mst.h"
```

Include dependency graph for graphviz.cpp:

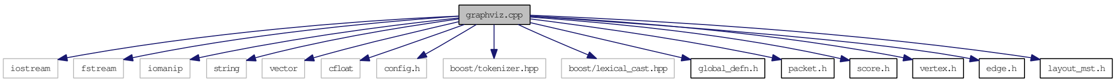

4.7 io.cpp File Reference

```
#include <iostream>
#include <fstream>
#include <iomanip>
#include <string>
#include <vector>
#include <cfloat>
#include "config.h"
#include <boost/tokenizer.hpp>
#include <boost/lexical_cast.hpp>
#include <boost/regex.hpp>
#include "global_defn.h"
#include "packet.h"
#include "score.h"
#include "vertex.h"
#include "edge.h"
#include "layout_mst.h"
```

Include dependency graph for io.cpp:

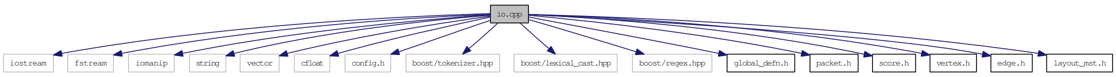

4.8 layout_mst.cpp File Reference

```
#include <string>
#include <vector>
#include <cstdlib>
#include "config.h"
#include "global_defn.h"
#include "check.h"
#include "packet.h"
#include "score.h"
#include "vertex.h"
#include "edge.h"
#include "layout_mst.h"
```

Include dependency graph for layout_mst.cpp:

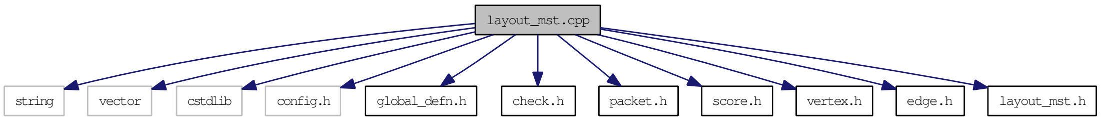

4.9 layout_mst.h File Reference

This graph shows which files directly or indirectly include this file:

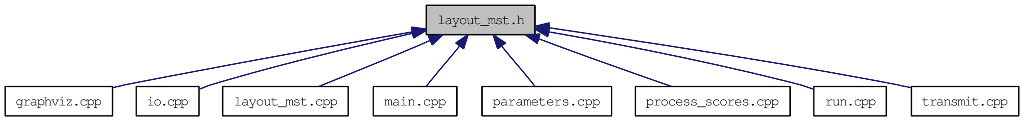

Classes

- class LAYOUTMST

4.10 main.cpp File Reference

```
#include <iostream>
#include <string>
#include <vector>
#include <cstdlib>
#include "config.h"
#include "global_defn.h"
#include "packet.h"
#include "score.h"
#include "vertex.h"
#include "edge.h"
#include "layout_mst.h"
```

Include dependency graph for main.cpp:

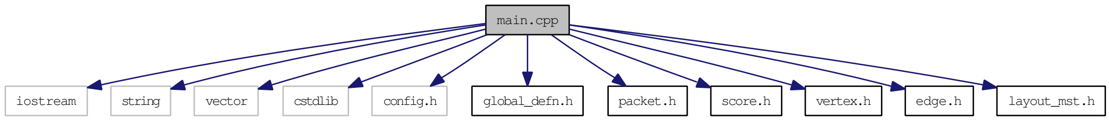

Functions

- int main (int argc, char *argv[ ])

    The main () function of the program.

4.10.1 Function Documentation

4.10.1.1 int main (int *argc*, char * *argv*[ ])

The main () function of the program.

Create a LAYOUTMST object and then uses it to read in the parameters from the file and the command line. If all the settings check out, then run the main program.

4.11 packet.cpp File Reference

```
#include <string>
#include <vector>
#include "config.h"
#include <boost/serialization/string.hpp>
#include <boost/serialization/vector.hpp>
#include "global_defn.h"
#include "packet.h"
```

Include dependency graph for packet.cpp:

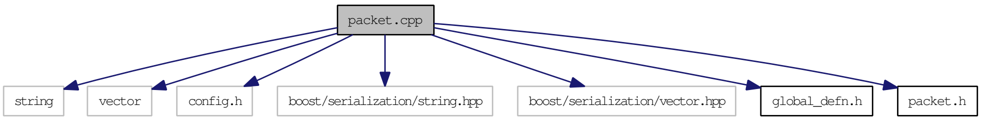

4.12 packet.h File Reference

This graph shows which files directly or indirectly include this file:

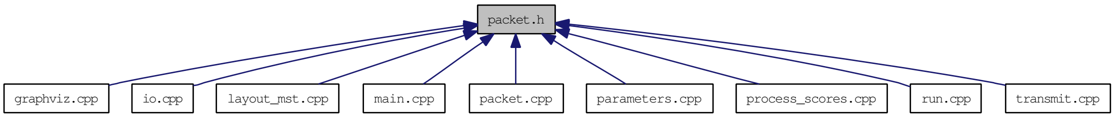

Classes

- class PACKET

4.13 parameters.cpp File Reference

```
#include <iostream>
#include <iomanip>
#include <fstream>
#include <string>
#include <vector>
#include "config.h"
#include <boost/program_options.hpp>
#include "global_defn.h"
#include "packet.h"
#include "score.h"
#include "vertex.h"
#include "edge.h"
#include "layout_mst.h"
```

Include dependency graph for parameters.cpp:

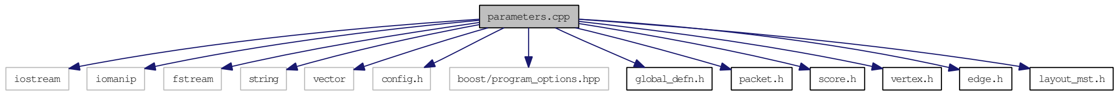

4.14 process_scores.cpp File Reference

```
#include <iostream>
#include <fstream>
#include <string>
#include "config.h"
#include <boost/tokenizer.hpp>
#include <boost/lexical_cast.hpp>
#include "global_defn.h"
#include "packet.h"
#include "score.h"
#include "vertex.h"
#include "edge.h"
#include "layout_mst.h"
```

Include dependency graph for process_scores.cpp:

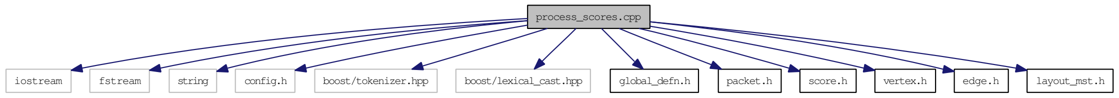

Functions

- bool scoresGreaterCmp (SCORE x, SCORE y)

  Comparison function for sorting scores into decreasing order by combined score.

- bool scoresGreaterIDCmp (SCORE x, SCORE y)

  Comparison function for sorting scores into increasing order by ID.

4.14.1 Function Documentation

4.14.1.1 bool scoresGreaterCmp (SCORE *x*, SCORE *y*)

Comparison function for sorting scores into decreasing order by combined score.

Here is the call graph for this function:

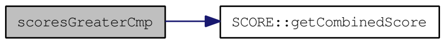

4.14.1.2 bool scoresGreaterIDCmp (SCORE *x*, SCORE *y*)

Comparison function for sorting scores into increasing order by ID.

Here is the call graph for this function:

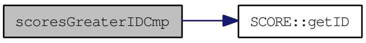

4.15 run.cpp File Reference

```
#include <iostream>
#include <string>
#include <vector>
#include "config.h"
#include "global_defn.h"
#include "packet.h"
#include "score.h"
#include "vertex.h"
#include "edge.h"
#include "layout_mst.h"
```

Include dependency graph for run.cpp:

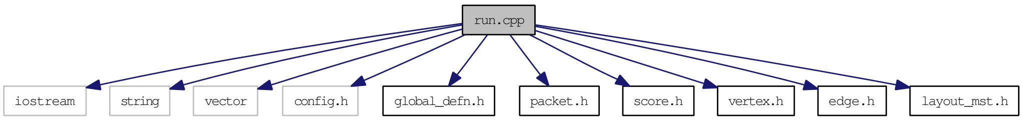

4.16 score.cpp File Reference

```
#include <string>
#include <vector>
#include <climits>
#include <cmath>
#include "global_defn.h"
#include "score.h"
```

Include dependency graph for score.cpp:

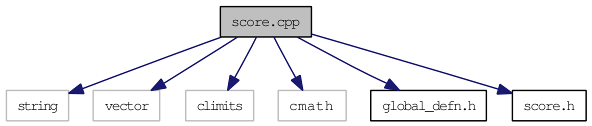

4.17 score.h File Reference

This graph shows which files directly or indirectly include this file:

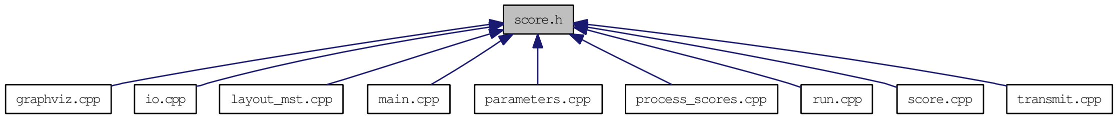

Classes

- class SCORE

4.18 transmit.cpp File Reference

#include <string>

#include <vector>

#include "config.h"

#include <boost/serialization/vector.hpp>

#include "global_defn.h"

#include "score.h"

#include "packet.h"

#include "vertex.h"

#include "edge.h"

#include "layout_mst.h"

Include dependency graph for transmit.cpp:

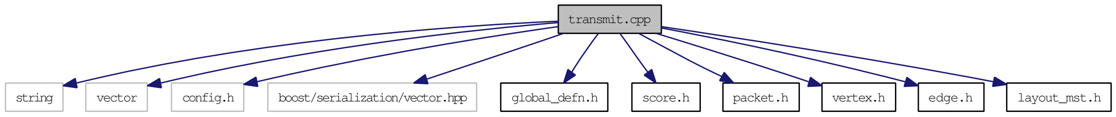

4.19 vertex.cpp File Reference

`#include <string>`

`#include "vertex.h"`

Include dependency graph for vertex.cpp:

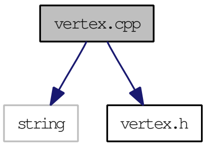

4.20 vertex.h File Reference

This graph shows which files directly or indirectly include this file:

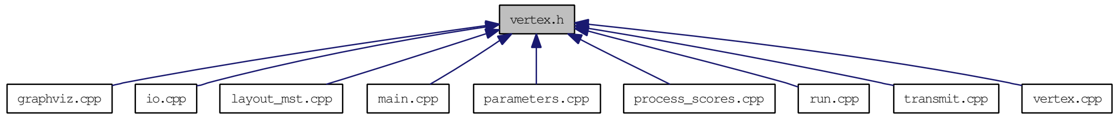

Classes

- class VERTEX

Index

all_workunits
    LAYOUTMST, 35

broadcastData
    LAYOUTMST, 22

callGraphViz
    LAYOUTMST, 25
CFG_FILENAME
    global_defn.h, 60
check.cpp, 53
    sanitizeFilename, 53
    sanitizePath, 53
    sanitizeURL, 54
check.h, 55
    sanitizeFilename, 55
    sanitizePath, 55
    sanitizeURL, 55
checkSettings
    LAYOUTMST, 14
colour
    VERTEX, 52
combined
    SCORE, 47
components
    VERTEX, 52

debug_flag
    LAYOUTMST, 33
    PACKET, 42
DEFAULT_DPI
    global_defn.h, 60
DEFAULT_FONTSIZE
    global_defn.h, 60
DEFAULT_HEIGHT
    global_defn.h, 60
DEFAULT_PERCENT
    global_defn.h, 60
DEFAULT_PHEIGHT
    global_defn.h, 60
DEFAULT_PWIDTH
    global_defn.h, 60
DEFAULT_URL
    global_defn.h, 60
DEFAULT_WIDTH
    global_defn.h, 60
DOT_FILE_EXTENSION
    global_defn.h, 60
dpi
    LAYOUTMST, 34
    PACKET, 43

EDGE, 5
    EDGE, 6
    end, 7
    getEnd, 6
    getStart, 6
    getWeight, 7
    setEnd, 6
    setStart, 6
    setWeight, 7
    start, 7
    weight, 7
edge.cpp, 56
edge.h, 57
edges
    LAYOUTMST, 34
EDGES_FILE_EXTENSION
    global_defn.h, 60
ENABLE_LARGE_NODE
    global_defn.h, 60
end
    EDGE, 7
expandPacket
    LAYOUTMST, 20

FILETYPE
    global_defn.h, 62
FILETYPE_CMAP
    global_defn.h, 62
FILETYPE_DOT
    global_defn.h, 62
FILETYPE_GV
    global_defn.h, 62
FILETYPE_PNG
    global_defn.h, 62
FILETYPE_PS
    global_defn.h, 62
FILETYPE_SVG
    global_defn.h, 62

FILETYPE_UNSET
global_defn.h, 62
fixed_pos
LAYOUTMST, 33
PACKET, 42
fontsize
LAYOUTMST, 34
PACKET, 43

getAllWorkunits
LAYOUTMST, 32
getColour
VERTEX, 50
getCombinedScore
SCORE, 47
getComponents
VERTEX, 51
getDebug
LAYOUTMST, 29
PACKET, 39
getDPI
LAYOUTMST, 31
PACKET, 41
getEnd
EDGE, 6
getFixedPos
LAYOUTMST, 29
PACKET, 40
getFontsize
LAYOUTMST, 31
PACKET, 42
getHeight
LAYOUTMST, 30
PACKET, 40
VERTEX, 51
getID
SCORE, 46
getLeft
SCORE, 46
getMyWorkunit
LAYOUTMST, 32
PACKET, 42
getName
VERTEX, 50
getOuttype
LAYOUTMST, 31
PACKET, 41
getPath
LAYOUTMST, 29
PACKET, 40
getPercent
LAYOUTMST, 31
PACKET, 41
getPHeight
LAYOUTMST, 30
PACKET, 41
getPreview
LAYOUTMST, 29
PACKET, 39
getPWidth
LAYOUTMST, 30
PACKET, 40
getRank
LAYOUTMST, 32
getRight
SCORE, 46
getScore1
SCORE, 46
getScore2
SCORE, 46
getScoresFn
LAYOUTMST, 32
getShape
VERTEX, 50
getSpline
LAYOUTMST, 31
PACKET, 41
getStart
EDGE, 6
getTotalIter
LAYOUTMST, 31
getUpdated
VERTEX, 51
getURL
LAYOUTMST, 30
PACKET, 40
getVerbose
LAYOUTMST, 29
PACKET, 39
getWeight
EDGE, 7
getWidth
LAYOUTMST, 30
PACKET, 40
VERTEX, 51
getWorldSize
LAYOUTMST, 33
getX
VERTEX, 51
getY
VERTEX, 51
global_defn.h
FILETYPE_CMAP, 62
FILETYPE_DOT, 62
FILETYPE_GV, 62
FILETYPE_PNG, 62
FILETYPE_PS, 62
FILETYPE_SVG, 62

FILETYPE_UNSET, 62
global_defn.h, 58
CFG_FILENAME, 60
DEFAULT_DPI, 60
DEFAULT_FONTSIZE, 60
DEFAULT_HEIGHT, 60
DEFAULT_PERCENT, 60
DEFAULT_PHEIGHT, 60
DEFAULT_PWIDTH, 60
DEFAULT_URL, 60
DEFAULT_WIDTH, 60
DOT_FILE_EXTENSION, 60
EDGES_FILE_EXTENSION, 60
ENABLE_LARGE_NODE, 60
FILETYPE, 62
GV_FILE_EXTENSION, 61
GVPV_FILE_EXTENSION, 61
LARGE_NODE_INCREASE, 61
LARGE_NODE_THRESH, 61
MAX_DIM, 61
MAX_DPI, 61
MAX_FONTSIZE, 61
MIN_DIM, 61
MIN_DPI, 61
MIN_FONTSIZE, 61
NODES_FILE_EXTENSION, 61
SCALE_FACTOR, 62
SCORES_FIELDS, 62
VERBOSE_WIDTH, 62
graphviz.cpp, 63
GV_FILE_EXTENSION
global_defn.h, 61
GVPV_FILE_EXTENSION
global_defn.h, 61

height
LAYOUTMST, 33
PACKET, 42
VERTEX, 52

id
SCORE, 47
initSettings
LAYOUTMST, 14
io.cpp, 64

LARGE_NODE_INCREASE
global_defn.h, 61
LARGE_NODE_THRESH
global_defn.h, 61
layout_mst.cpp, 65
layout_mst.h, 66
LAYOUTMST, 8
all_workunits, 35
broadcastData, 22
callGraphViz, 25
checkSettings, 14
debug_flag, 33
dpi, 34
edges, 34
expandPacket, 20
fixed_pos, 33
fontsize, 34
getAllWorkunits, 32
getDebug, 29
getDPI, 31
getFixedPos, 29
getFontsize, 31
getHeight, 30
getMyWorkunit, 32
getOuttype, 31
getPath, 29
getPercent, 31
getPHeight, 30
getPreview, 29
getPWidth, 30
getRank, 32
getScoresFn, 32
getSpline, 31
getTotalIter, 31
getURL, 30
getVerbose, 29
getWidth, 30
getWorldSize, 33
height, 33
initSettings, 14
LAYOUTMST, 13
my_workunit, 35
outtype, 34
path, 33
percent, 34
pheight, 34
preview_flag, 33
printGraphViz, 27
process, 26
processOptions, 13
processScores, 20
pwidth, 33
rank, 35
readEdges, 28
readNodes, 27
readScores, 19
receiveData, 24
recvOKFail, 15
runAllProcessors, 18
runPrimary, 16
scores, 34
scores_fn, 34

sendOKFail, 15
setAllWorkunits, 32
setDebug, 28
setDPI, 31
setFixedPos, 29
setFontsize, 31
setHeight, 30
setMyWorkunit, 32
setOuttype, 30
setPath, 29
setPercent, 31
setPHeight, 30
setPreview, 29
setPWidth, 30
setRank, 32
setScoresFn, 32
setSpline, 31
setTotalIter, 31
setURL, 29
setVerbose, 29
setWidth, 30
setWorldSize, 33
spline_flag, 34
total_iter, 34
updateNodePositions, 28
url, 33
verbose_flag, 33
vertices, 34
width, 33
world_size, 35
left
SCORE, 47

main
main.cpp, 67
main.cpp, 67
main, 67
MAX_DIM
global_defn.h, 61
MAX_DPI
global_defn.h, 61
MAX_FONTSIZE
global_defn.h, 61
MIN_DIM
global_defn.h, 61
MIN_DPI
global_defn.h, 61
MIN_FONTSIZE
global_defn.h, 61
my_workunit
LAYOUTMST, 35
PACKET, 43

name
VERTEX, 52
NODES_FILE_EXTENSION
global_defn.h, 61

operator<
SCORE, 47
operator>
SCORE, 47
outtype
LAYOUTMST, 34
PACKET, 43

PACKET, 36
debug_flag, 42
dpi, 43
fixed_pos, 42
fontsize, 43
getDebug, 39
getDPI, 41
getFixedPos, 40
getFontsize, 42
getHeight, 40
getMyWorkunit, 42
getOuttype, 41
getPath, 40
getPercent, 41
getPHeight, 41
getPreview, 39
getPWidth, 40
getSpline, 41
getURL, 40
getVerbose, 39
getWidth, 40
height, 42
my_workunit, 43
outtype, 43
PACKET, 39
path, 42
percent, 43
pheight, 43
preview_flag, 42
pwidth, 43
setDebug, 39
setDPI, 41
setFixedPos, 39
setFontsize, 41
setHeight, 40
setMyWorkunit, 42
setOuttype, 41
setPath, 40
setPercent, 41
setPHeight, 41
setPreview, 39
setPWidth, 40

setSpline, 41
setURL, 40
setVerbose, 39
setWidth, 40
spline_flag, 43
url, 42
verbose_flag, 42
width, 42
packet.cpp, 68
packet.h, 69
parameters.cpp, 70
path
LAYOUTMST, 33
PACKET, 42
percent
LAYOUTMST, 34
PACKET, 43
pheight
LAYOUTMST, 34
PACKET, 43
preview_flag
LAYOUTMST, 33
PACKET, 42
printGraphViz
LAYOUTMST, 27
process
LAYOUTMST, 26
process_scores.cpp, 71
scoresGreaterCmp, 71
scoresGreaterIDCmp, 71
processOptions
LAYOUTMST, 13
processScores
LAYOUTMST, 20
pwidth
LAYOUTMST, 33
PACKET, 43

rank
LAYOUTMST, 35
readEdges
LAYOUTMST, 28
readNodes
LAYOUTMST, 27
readScores
LAYOUTMST, 19
receiveData
LAYOUTMST, 24
recvOKFail
LAYOUTMST, 15
right
SCORE, 47
run.cpp, 73
runAllProcessors
LAYOUTMST, 18
runPrimary
LAYOUTMST, 16

sanitizeFilename
check.cpp, 53
check.h, 55
sanitizePath
check.cpp, 53
check.h, 55
sanitizeURL
check.cpp, 54
check.h, 55
SCALE_FACTOR
global_defn.h, 62
SCORE, 44
combined, 47
getCombinedScore, 47
getID, 46
getLeft, 46
getRight, 46
getScore1, 46
getScore2, 46
id, 47
left, 47
operator<, 47
operator>, 47
right, 47
SCORE, 45
score1, 47
score2, 47
setCombinedScore, 46
setID, 46
setLeft, 46
setRight, 46
setScore1, 46
setScore2, 46
score.cpp, 74
score.h, 75
score1
SCORE, 47
score2
SCORE, 47
scores
LAYOUTMST, 34
SCORES_FIELDS
global_defn.h, 62
scores_fn
LAYOUTMST, 34
scoresGreaterCmp
process_scores.cpp, 71
scoresGreaterIDCmp
process_scores.cpp, 71
sendOKFail

LAYOUTMST, 15
setAllWorkunits
LAYOUTMST, 32
setColour
VERTEX, 50
setCombinedScore
SCORE, 46
setComponents
VERTEX, 50
setDebug
LAYOUTMST, 28
PACKET, 39
setDPI
LAYOUTMST, 31
PACKET, 41
setEnd
EDGE, 6
setFixedPos
LAYOUTMST, 29
PACKET, 39
setFontsize
LAYOUTMST, 31
PACKET, 41
setHeight
LAYOUTMST, 30
PACKET, 40
VERTEX, 51
setID
SCORE, 46
setLeft
SCORE, 46
setMyWorkunit
LAYOUTMST, 32
PACKET, 42
setName
VERTEX, 50
setOuttype
LAYOUTMST, 30
PACKET, 41
setPath
LAYOUTMST, 29
PACKET, 40
setPercent
LAYOUTMST, 31
PACKET, 41
setPHeight
LAYOUTMST, 30
PACKET, 41
setPreview
LAYOUTMST, 29
PACKET, 39
setPWidth
LAYOUTMST, 30
PACKET, 40
setRank
LAYOUTMST, 32
setRight
SCORE, 46
setScore1
SCORE, 46
setScore2
SCORE, 46
setScoresFn
LAYOUTMST, 32
setShape
VERTEX, 50
setSpline
LAYOUTMST, 31
PACKET, 41
setStart
EDGE, 6
setTotalIter
LAYOUTMST, 31
setUpdated
VERTEX, 51
setURL
LAYOUTMST, 29
PACKET, 40
setVerbose
LAYOUTMST, 29
PACKET, 39
setWeight
EDGE, 7
setWidth
LAYOUTMST, 30
PACKET, 40
VERTEX, 51
setWorldSize
LAYOUTMST, 33
setX
VERTEX, 51
setY
VERTEX, 51
shape
VERTEX, 52
spline_flag
LAYOUTMST, 34
PACKET, 43
start
EDGE, 7

total_iter
LAYOUTMST, 34
transmit.cpp, 76

updated
VERTEX, 52
updateNodePositions

LAYOUTMST, 28
url
LAYOUTMST, 33
PACKET, 42

verbose_flag
LAYOUTMST, 33
PACKET, 42
VERBOSE_WIDTH
global_defn.h, 62
VERTEX, 48
colour, 52
components, 52
getColour, 50
getComponents, 51
getHeight, 51
getName, 50
getShape, 50
getUpdated, 51
getWidth, 51
getX, 51
getY, 51
height, 52
name, 52
setColour, 50
setComponents, 50
setHeight, 51
setName, 50
setShape, 50
setUpdated, 51
setWidth, 51
setX, 51
setY, 51
shape, 52
updated, 52
VERTEX, 50
width, 52
x, 52
y, 52
vertex.cpp, 77
vertex.h, 78
vertices
LAYOUTMST, 34

weight
EDGE, 7
width
LAYOUTMST, 33
PACKET, 42
VERTEX, 52
world_size
LAYOUTMST, 35

x
VERTEX, 52

y
VERTEX, 52
